# Remote monitoring technologies for measuring cardiovascular functions in community-dwelling adults: a systematic review

**DOI:** 10.1007/s11357-023-00815-4

**Published:** 2023-05-18

**Authors:** Jessica K. Lu, Marcella Sijm, Georges E. Janssens, Jorming Goh, Andrea B. Maier

**Affiliations:** 1 Centre for Healthy Longevity, National University Health System, Singapore, Singapore; 2 Healthy Longevity Translational Research Program, Yong Loo Lin School of Medicine, National University of Singapore, Singapore, Singapore; 3 University of Amsterdam, Amsterdam, The Netherlands; 4 Laboratory Genetic Metabolic Diseases, Amsterdam University Medical Centers – location Academic Medical Center, University of Amsterdam, Amsterdam, The Netherlands; 5 Department of Physiology, Yong Loo Lin School of Medicine, National University of Singapore, Singapore, Singapore; 6 Department of Human Movement Sciences, Faculty of Behavioural and Movement Sciences, Vrije Universiteit Amsterdam, Amsterdam Movement Sciences, Van der Boechorstsraat 7, 1081 BT Amsterdam, The Netherlands

**Keywords:** Digital technology, Monitoring, Physiologic, Wearable electronic devices, Cardiovascular physiological phenomena, Aging

## Abstract

Remote monitoring technologies (RMTs) allow continuous, unobtrusive, and real-time monitoring of the cardiovascular system. An overview of existing RMTs measuring cardiovascular physiological variables is lacking. This systematic review aimed to describe RMTs measuring cardiovascular functions in community-dwelling adults. An electronic search was conducted via PubMed, EMBASE, and Cochrane Library from January 1, 2020, to April 7, 2022. Articles reporting on non-invasive RMTs used unsupervised in community-dwelling adults were included. Reviews and studies in institutionalized populations were excluded. Two reviewers independently assessed the studies and extracted the technologies used, cardiovascular variables measured, and wearing locations of RMTs. Validation of the RMTs was examined based on the COSMIN tool, and accuracy and precision were presented. This systematic review was registered with PROSPERO (CRD42022320082). A total of 272 articles were included representing 322,886 individuals with a mean or median age from 19.0 to 88.9 years (48.7% female). Of all 335 reported RMTs containing 216 distinct devices, photoplethysmography was used in 50.3% of RMTs. Heart rate was measured in 47.0% of measurements, and the RMT was worn on the wrist in 41.8% of devices. Nine devices were reported in more than three articles, of which all were sufficiently accurate, six were sufficiently precise, and four were commercially available in December 2022. The top four most reported technologies were AliveCor KardiaMobile®, Fitbit Charge 2, and Polar H7 and H10 Heart Rate Sensors. With over 200 distinct RMTs reported, this review provides healthcare professionals and researchers an overview of available RMTs for monitoring the cardiovascular system.

**Supplementary Information:**

The online version contains supplementary material available at 10.1007/s11357-023-00815-4.

## Introduction

Optimal functioning of the cardiovascular system enables the human body to meet demands for exercise, recuperation, and combatting stress [1]. By tracking the cardiovascular physiological variables affected by daily activities, the level of decline during the ageing process and improvement due to interventions of the cardiovascular system can be monitored longitudinally. In addition to knowing the real-time status, continuous monitoring of cardiovascular variables helps identify indicators of current or future health problems [2].

Remote monitoring technologies (RMTs) are devices that measure, analyse, and/or transmit data about the physiological status of an individual [3, 4]. They can do so consistently, even outside clinicians’ offices and hospitals [2]. Blood pressure monitors, mobile electrocardiograms, and other wearable devices, such as smartwatches and fitness trackers, are examples of RMTs, and the number of offerings has burgeoned in the past five years [4]. However, not all devices are validated and accurate [3] and consumer devices are not required to undergo regulatory approval by the US Food and Drug Administration (US FDA). Of 1291 articles on PubMed citing 39 common wearable devices measuring cardiovascular variables, only 14 devices are FDA-cleared as of October 2020 [5]. The measurement accuracy can also be affected by different activities (e.g., rest versus exercise), leading to errors in data collection and subsequent analyses [6]. The myriad devices available on the market vary in sensor technology, what they measure, wearing location, and accuracy; however, an overview of existing RMTs measuring cardiovascular variables is lacking.

This systematic review aimed to describe RMTs measuring cardiovascular functions in community-dwelling adults. Subsequently, the accuracy and precision of the RMTs reported in more than three studies were examined.

## Methods

The protocol for this systematic review was registered in the PROSPERO International Prospective Register of Systematic Reviews (registration number: CRD42022320082). The Preferred Reporting Items for Systematic Reviews and Meta-Analysis (PRISMA) [7] statement and Synthesis Without Meta-Analysis (SWiM) [8] guidelines were used to guide the reporting of this review.

### Search strategy and selection criteria

The systematic search was conducted from January 1, 2020, to April 7, 2022, using three electronic databases: PubMed, EMBASE, and Cochrane Library. The search strategy was developed with a librarian from the National University of Singapore, Annelissa Chin, and included keywords related to digital health, monitoring, physiological variables and cardiovascular system, and population. The search strategy is provided (appendix p 3). The articles were organized and managed using the Covidence systematic review software (Veritas Health Innovation, Melbourne, Australia) and EndNote™ citation manager (Clarivate Analytics, version X9.3.3).

Articles were included if they described original studies independent of the study design, published in English, and the full text could be obtained. Studies could have been conducted in any environment (e.g., clinics, controlled laboratory environments, at home), but human individuals needed to be community-dwelling. All sexes and health statuses were included. The article needed to report the unsupervised use of a non-invasive RMT (including brand and model) that measured cardiovascular variables, which were defined as physiological measures of the cardiovascular system, such as heart rate (HR), heart rate variability, and blood pressure (BP). The measurements needed to be directly accessible by the user, whether shown on the device display, a connected device (e.g., a smartphone), or wirelessly sent to a user-accessible electronic health record.

Reviews, opinion articles, clinical trial registries, study protocols, and animal and in vitro studies were excluded. Articles reporting on non-community-dwelling individuals (e.g., people in nursing homes, inpatients), pregnant women from whom measurements only pertaining to the foetus were taken, and cohorts with a mean or median age under 18 years old were excluded. Articles were excluded if cardiovascular variables measured were not reported and if an external party (e.g., laboratory or medical professional) was required for measurements and/or retrieval of the results measured by the technology. Mobile applications taking input of measurements by other technologies (e.g., the user manually enters the BP reading measured by a nondescript (i.e., no brand or model) sphygmomanometer in a mobile application), invasive technologies (e.g., implantable cardiac defibrillators), and contact-free devices installed in the surrounding environment were excluded.

Covidence was used during the selection process. Articles were independently screened for eligibility based on their titles and abstracts by two reviewers (JKL, MS). Full texts of eligible articles were screened independently by the same reviewers to obtain articles for full-text inclusion. Potential disagreement on the eligibility of the articles was solved by an additional reviewer (JG).

Data were independently extracted by two reviewers (JKL, MS) for 50 (30 of the most recently published and 20 randomly selected) articles to test consistency in data extraction. Data from the remaining included articles were extracted by JKL. The extracted variables were study characteristics (first author, publication year, country, and study design); participant characteristics (mean or median age, sample size, percentage of females, and population characteristics); and characteristics of the RMT (model (company, city, province/state/country, hardware version), software/mobile application(s) (version), measurement technology and processing/analysis algorithm(s) (version), cardiovascular physiological variable(s) measured, wearing location, and method(s) used to assess the accuracy and precision of the RMT and the validation outcomes). Disagreement in data extraction was resolved by a third reviewer (JG). Microsoft Excel (Version 16.67) was used for data extraction.

The quality of 50 included articles (30 of the most recently published and 20 randomly selected) were critically appraised independently by two reviewers (JKL, MS). The Newcastle–Ottawa Scale (NOS) checklist was adapted to assess the quality of observational studies (appendix p 4) [9]. The Cochrane Risk of Bias (RoB) Tool (version 2.0) was used to assess the quality of randomized controlled trials (RCTs) [10] and the Risk Of Bias In Non-randomised Studies-of Interventions (ROBINS-I) Tool was used to assess the quality of all other interventional studies [11].

Validation refers to confirming via objective evidence that a particular device can consistently fulfill the requirements for a specific intended use (FDA 21CFR§820.3(z)) with sufficient accuracy and precision (European Union Medical Device Regulations 2017/745, L117). Under this Regulation, accuracy is defined as the trueness (the true value) and precision (repeatability and reproducibility) of the measurement.

For RMTs that were reported in more than three studies, accuracy and precision (compared against a reference standard) were extracted from the included articles and the references of included articles. If articles and their references did not describe accuracy, the website of the brand/RMT was searched. The user manual of the COSMIN Risk of Bias tool was used to guide the reporting of validation outcomes [12]. For accuracy, the reported measures were the standard error of measurement (SEM), limits of agreement (LoA), or the coefficient of variation for continuous scores; sensitivity (SE)/specificity (SP)/positive predictive value (PPV)/negative predictive value (NPV) for dichotomous/nominal/ordinal scores. For precision, the reported measures were the intraclass correlation coefficient (ICC), Lin’s concordance correlation coefficient (CCC), Pearson’s correlation coefficient ($r$), or Spearman’s correlation coefficient ($\rho$) for continuous scores; Cohen’s (weighted) kappa ($\kappa$) for dichotomous/nominal/ordinal scores. The reference standard, conclusions of the validation studies, population studied, sample size, and whether the RMT was commercially available in December 2022 were presented. Sufficient accuracy was determined using the conclusions from the validation studies. Sufficient precision was defined as the correlation coefficient or kappa equal to or greater than 0.70 [12].

### Data analysis

The retrieved data was reported following the PRISMA statement [7] and SWiM guidelines [8]. The PRISMA flow diagram was used to present the selection procedure [7]. Descriptive statistics were used to summarize the participants’ characteristics. Age in years was stated in mean (standard deviation), median [interquartile range], or (mean or median*) {range}. Each RMT characteristic was tabulated once per device: e.g., a RMT using photoplethysmography and oscillometry that measures HR, blood oxygenation, and BP was tabulated as two technologies and three cardiovascular variables. Chi-square tests were conducted to test whether there is an association between the characteristics of the RMTs used and the study quality. High-quality observational studies and interventional studies with low risk of bias were categorised as high-quality studies. Low-quality observational studies, RCTs with some concerns and high risk of bias, and other interventional studies with moderate and serious risk of bias were categorized as low-quality studies. Microsoft Excel (Version 16.67) was used to calculate the chi-square value. Microsoft PowerPoint (Version 16.67) was used to create the figures.

## Results

**Fig. 1:**
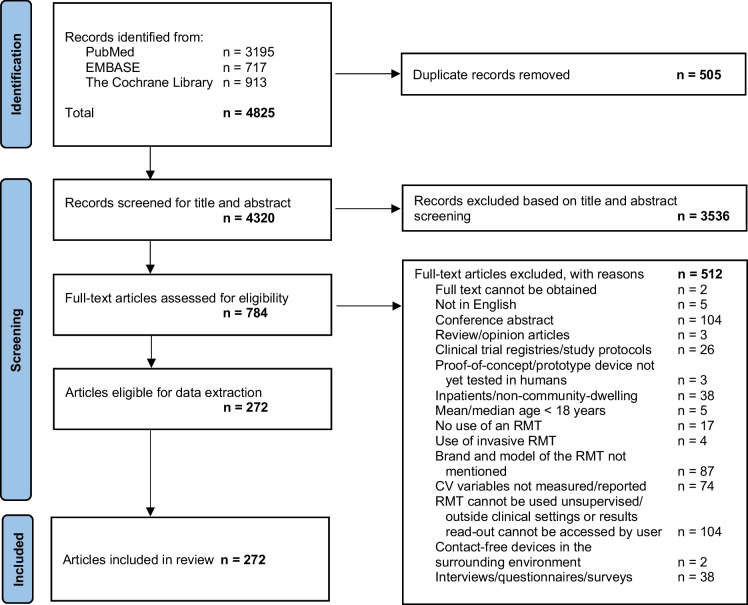
PRISMA diagram illustrating the study selection process. CV, cardiovascular; RMT, remote monitoring technology

The article selection process is shown in the PRISMA diagram (Fig. 1). A total of 4,825 records were identified from three electronic databases, of which 4,320 articles remained after duplicates were removed. These articles were screened for title and abstract, of which 784 full-text articles were assessed for eligibility. A total of 272 articles were included in this systematic review.

The characteristics of the included articles are presented (appendix pp 5–11). There were 68 (25.0%) studies conducted in the USA with the remainder in 47 other countries. Of the 236 observational and 38 interventional studies, two articles reported an observational study followed by an interventional study [13, 14]. A total of 322,886 individuals (48.7% females) were represented (range: 1 to 92,457 individuals per article). The mean or median age of each study population ranged from 19.0 to 88.9 years. The studied populations in 54 (19.9%) articles were healthy.

**Fig. 2:**
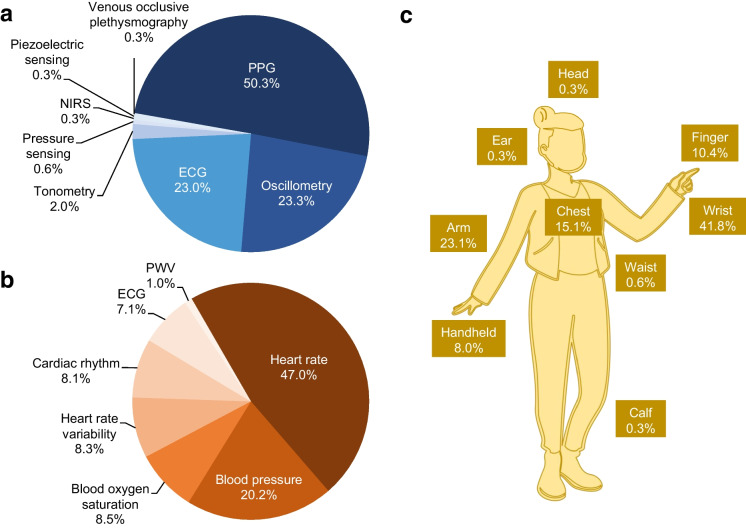
Frequencies of the technologies, cardiovascular physiological variables measured, and wearing locations of the remote monitoring technologies (RMTs). Frequency is the count of technologies, measurements, and locations that are examined in the 272 included studies. A) Percentages represent the proportion of the indicated technology over all technologies used (*n* = 344). ECG, electrocardiography; NIRS, near-infrared spectroscopy; PPG, photoplethysmography. B) Percentages represent the proportion of the indicated variable over all cardiovascular physiological variables measured (*n* = 496). ECG, electrocardiogram; PWV, pulse wave velocity. C) Percentages represent the proportion of the indicated wearing location over all wearing locations of the RMTs (*n* = 337)

The total number of devices reported was 335, representing 216 distinct devices and 57 devices reported in a minimum of two articles. The characteristics of the RMTs used in each article are presented (appendix pp 12–24). Technologies used include photoplethysmography (PPG), oscillometry, electrocardiography (ECG), tonometry, pressure sensing, near-infrared spectroscopy, piezoelectric sensing, and venous occlusive plethysmography. The cardiovascular variables measured include heart rate (HR); systolic, diastolic, and mean arterial blood pressure (BP); blood oxygen saturation; heart rate variability (HRV); cardiac rhythm; electrical heart activity (ECG); and pulse wave velocity. The reported wearing locations include the wrist, arm, chest, finger, waist, head, ear, and calf. Handheld RMTs were also reported. Figure 2 presents a summary of the technologies, cardiovascular variables measured, and wearing locations of the RMTs. PPG was used in 173 (50.3%) of the total number of technologies and HR was measured in 233 (47.0%) of all reported measurements. Wrist-based devices comprised 141 (41.8%) of all reported RMTs. A summary of the measured variables reported for the 216 distinct devices is presented (appendix pp 25–30). The AliveCor KardiaMobile® handheld ECG was reported in 11 studies, Fitbit Charge 2 in ten, Polar H7 and H10 Heart Rate Sensors each in nine, Spacelabs 90,207 BP monitor (BPM) in eight, Fitbit Charge HR in seven, Fitbit Charge 3 and Itamar Watch-PAT 200 each in five, Omron HEM-9200 T BPM in four, and the remaining RMTs in three or fewer studies. Regarding the 102 distinct brands of the RMTs, Fitbit was reported in 42 studies, Polar in 31, Omron in 23, Apple in 22, AliveCor and Spacelabs each in 14, Garmin and Samsung each in 13, and the remaining brands were reported in ten or fewer studies.

The study quality and risk of bias of 50 included articles is presented. The overall quality of the 41 observational studies was high (appendix p 31). The four randomized controlled trials (appendix p 32) and six other interventional studies (appendix p 33) had an overall moderate risk of bias. No statistically significant association between the characteristics of the RMTs used and the study quality was detected $(p>0.050)$ (appendix p 34).

The validation outcomes of RMTs reported in more than three studies (nine devices in total) are provided in Table 1. Reference standards include multi-lead ECGs (3-, 4-, 5-, and 12-leads), mercury sphygmomanometers (with auscultation), and finger pulse oximeters. Accuracy was reported using SEM, LoA, and SE/SP/PPV/NPV, and precision using ICC, CCC, $r$, $\rho$, and $\kappa$. Accuracy outcomes were extracted for the nine RMTs, and all were sufficiently accurate. Precision outcomes were available for six of the nine RMTs, and the six RMTs were sufficiently precise for measuring select cardiovascular variables (KardiaMobile® for cardiac rhythm, Fitbit Charge 2 for HR, Polar H7 for HR and HRV, Polar H10 for HRV, Fitbit Charge HR for HR, and Watch-Pat 200 for blood oxygen saturation). Two different algorithms for the Polar H10 produced different accuracy and precision outcomes [15]. Four of the nine RMTs were commercially available (KardiaMobile®, Polar H10, Spacelabs 90207 BPM, and Omron HEM-9200 T BPM) in December 2022 (Table 2). These four RMTs were sufficiently accurate to measure the indicated cardiovascular variables in community-dwelling populations.

**Table 1:** Validation of the nine remote monitoring technologies that were reported in more than three studies

No. of studies	Remote monitoring technology	Reference standard	Cardiovascular variable	Accuracy (measurement error)	Precision (reliability)	Population studied (age in years), Sample size	Website accuracy/precision
11	AliveCor KardiaMobile®	12-lead ECG [16] leads I and II	HR (bpm) Cardiac rhythm: – QRS (ms) – QT (ms) – QTcB (ms)	Bias ± SEM: [17] 0·79 ± 5·36 1·55 ± 11·4 0·13 ± 19·1 1·29 ± 21·3	—	Routine primary care (mean 61), 125	—
		Professional evaluation of ECG recording	ECG classification: identification of AF or SR	SE: 95·3% SP: 97·5% PPV: 76·5% NPV: 99·6% [18]	—	Post-AF ablation (median 64·0), 115	SE: 96·6% SP: 94·0% $\kappa =0\cdot 89$ [19]
10	Fitbit Charge 2	12-lead ECG [16]	HR (bpm)	Bias (LoA): [20] –1·26 (–12·4–9·90)	CCC = 0·89 (0·84–0·92)	Healthy males during conventional walking (mean 23·7), 15	—
9	Polar H7 Heart Rate Sensor	12-lead ECG [16]	HR (bpm)	Bias (LoA): [20] –0·06 (–4·93–4·81)	CCC = 0·98 (0·97–0·99)	Healthy males during conventional walking (mean 23·7), 15	—
		3-lead ECG [21] leads I–III	HR variability: - RRi (ms) - SDNN (ms) - RMSSD (ms) - pNN50 (%) - LF (nu) - HF (nu) - LF/HF ratio	Bias (LoA): [22] 0·59 (–5·83–7·00) –2·07 (–12·6–8·42) –3·91 (–20·4–12·5) –0·87 (–4·23–2·49) –0·29 (–4·02–3·44) 0·33 (–4·07–4·74) –0·18 (–1·08–0·72)	$r=1\cdot 00$ $r=0\cdot 99$ $r=0\cdot 99$ $r=1\cdot 00$ $r=1\cdot 00$ $r=0\cdot 99$ $r=1\cdot 00$	Asymptomatic males with full function in performing ADL (mean 24·9), 8	—
9	Polar H10 Heart Rate Sensor	5-lead ECG [21]	HR (bpm)† HR variability (RMSSD (ms))†	Bias (LoA): [15] –0·32 (–1·92–1·29) –0·97 (–3·13–1·19) –6·98 (–34·7–20·8) –0·74 (–9·55–8·06)	— CCC = 0·77 CCC = 0·98	Healthy (mean 20·0), 5	—
		3-lead ECG [21]	RR interval (ms)	Bias (LoA): [23] 0·23 (26·6–27·1)	$\rho =1\cdot 00$	Healthy, lean, and physically fit (mean 24·7), 10	Within 2 ms accuracy 95·6% of the time [24]
8	Spacelabs 90207 Blood Pressure Monitor	Mercury sphygmo-manometer	SBP (mmHg) DBP (mmHg)	Bias ± SEM: [25] –1 ± 7 –3 ± 6	—	According to the BHS (range 15–80), 86	—; Passed AAMI, Good agreement (recommended) by the BHS [26]
7	Fitbit Charge HR	3-lead ECG [21]	HR (bpm) during SR	Bias (LoA): [27] < 1 (–11–11)	$r=0\cdot 87$	Holter monitoring (mean 68), 32	—
		3-lead ECG [21]	HR (bpm)	Bias ± SEM: [28] –0·66 ± 0·04	$r=0\cdot 93$	Under(graduate) students (mean 22·4), 25	—
5	Fitbit Charge 3	4-lead ECG [21]	HR (bpm)	Bias (LoA): [29] –11·4 (–53·8–30·9)	—	Community-dwelling (median 64), 20	—
5	Itamar Watch-PAT 200	Finger pulse oximetry during PSG	Oxygen desaturation	Bias ± SEM: [30] –1·6 ± 26·4 events per hour	ICC = 0·80	Suspected OSA (mean 43·2), 30	*r* (95% CI) = 0·88 (0·71–0·95) [31, 32]
4	Omron HEM-9200 T Blood Pressure Monitor	Auscultation, mercury sphygmo-manometer [21]	Blood pressure Pulse	—	—	—	$\pm 3 mmHg$ or 2% of reading $\pm 5\%$ of reading [33]

For data that were not reported, the notation “—” is used

^†^ Two entries are presented because the same device, but different processing algorithms were used

Abbreviations: *AAMI*, United States Association for the Advancement of Medical Instrumentation; *ADL*, activities of daily living; *AF*, atrial fibrillation; *BHS*, British Hypertension Society; *bpm*, beats per minute; *CI*, confidence interval; *ECG*, electrocardiogram; *HR*, heart rate; *mmHg*, millimeters of mercury; *ms*, milliseconds; *nu*, normalized units; *OSA*, obstructive sleep apnea; *PSG*, polysomnography; *QTcB*, corrected QT time by Bazett’s formula; *RMSSD*, root mean square of successive differences; *SR*, sinus rhythm

For accuracy (continuous scores): limit of agreement, LoA; standard error of measurement, SEM. For accuracy (dichotomous/nominal/ordinal scores): negative predictive value, NPV; positive predictive value, PPV; sensitivity, SE; specificity, SP

For precision (continuous scores): CCC, Lin’s concordance correlation coefficient; ICC, intraclass correlation coefficient; $r$, Pearson’s correlation coefficient; $\rho$, Spearman’s correlation coefficient. For precision (dichotomous/nominal/ordinal scores): $\kappa$, (weighted) Cohen’s kappa

**Table 2:** Summary of the validation of remote monitoring technologies reported in more than three studies and their commercial availability

Remote monitoring technology	Blood pressure		Cardiac rhythm		ECG recording		Heart rate		Heart rate variability		Blood oxygen saturation		Commercial availability
	Acc	Prec	Acc	Prec	Acc	Prec	Acc	Prec	Acc	Prec	Acc	Prec	
AliveCor KardiaMobile®			+	+	+	?	+	?					Yes
Fitbit Charge 2							+	+					Discontinued: outdated model
Polar H7 Heart Rate Sensor							+	+	+	+			Discontinued: outdated model
Polar H10 Heart Rate Sensor							+	?	+	+			Yes
Spacelabs 90207 Blood Pressure Monitor	+	?											Yes
Fitbit Charge HR							+	+					Discontinued: recalled
Fitbit Charge 3							+	?					Discontinued: outdated model
Itamar Watch-PAT 200											+	+	Discontinued: outdated model
Omron HEM-9200 T Blood Pressure Monitor	+	?					+	?					Yes

For use by the community-dwelling population: + : sufficient; ?: not reported; –: insufficient

Abbreviations: Acc, accuracy; ECG, electrocardiogram; Prec, precision

## Discussion

This systematic review identified 216 distinct RMTs for measuring cardiovascular functions with a quarter reported in a minimum of two studies. PPG was the technology used most often, and HR was the most frequently measured cardiovascular variable. Most devices were worn on the wrist. AliveCor KardiaMobile® was the most reported RMT, and Fitbit was the most reported brand. Of the nine RMTs reported in more than three studies, all were sufficiently accurate, six were sufficiently precise for measuring select cardiovascular variables, and four were commercially available in December 2022 (KardiaMobile®, Polar H10 HR Sensor, Spacelabs 90207 BPM, and Omron HEM-9200 T BPM).

The use of RMTs in clinical research has increased exponentially since the year 2000 [34]. Additionally, the use of wearable technology in daily life is on the rise: many consumers are wearing smartwatches and activity trackers outside of clinical and research contexts to continuously monitor their health [35], especially since the COVID-19 pandemic [36, 37]. Thus, the number of individuals using RMTs has skyrocketed and so has the number of studies incorporating the usage of RMTs [36, 37]. RMTs will become more integrated into daily life and in research as their multitude of uses (e.g., disease prevention, detection, treatment, and continual care and health information management) support the development and improvement of a technology-driven and accessible healthcare system [38].

Of the eight technologies used in the 335 total RMTs reported, half of the devices used PPG. The operation of PPG is simple, and it can be measured non-invasively [39]. The photoplethysmograph, the waveform signal output, provides information about the volumetric variations of blood circulation, and the processed second derivative of this waveform provides insights about different physiological measurements [40], such as HR and blood oxygenation. A major limitation is that PPG signals are extremely susceptible to interference, such as motion artifacts and environmental noise [41]; thus, estimation accuracy is lower during activities with more movement, such as physical exercise, compared to when measurements are taken at rest. Compared to oscillometry and ECG, the second and third most used technologies in the reported RMTs, the hardware of PPG sensors is easier to incorporate into a wearable device, has lower costs, and only a single sensor placed on the body is required for operation [39].

Among the seven different cardiovascular variables reported, HR was measured most often. HR is a critical indicator of health because it is interrelated with other cardiovascular variables and functions, such as BP, HR variability, cardiac rhythm, and electrical activity [21]. Furthermore, elevated resting HR has been associated with reduced lifespan [42] and increased risk of sudden cardiac death, coronary heart disease, heart failure, stroke, atrial fibrillation, cardiovascular disease, and all-cause mortality [43]. HR can be measured using a variety of technologies, three of which are also the most frequently used in the reported RMTs: PPG, oscillometry, and ECG. Hence, even if an RMT uses a different technology, the signal output can be processed to extract characteristics about HR. Thus, HR can be readily incorporated as an additional device measurement parameter and, consequently, monitored in healthcare and daily life settings.

Of the nine reported wearing locations, wearables worn at the wrist represent almost half of the RMTs reported, likely because they are highly portable, convenient and comfortable to wear, and relatively inexpensive [39]. Similar limitations arise, however: motion artifacts and environmental noise, especially during moderate and vigorous intensity physical activities, can interfere with the output signal and increase the inaccuracy of measurements [44]. While measurements from the arm and chest increase accuracy during activities requiring greater ranges of motion, wristband-like devices do not introduce discomfort and are increasingly popular for use in both medical grade and commercial consumer devices [45].

The nine RMTs reported in more than three studies were all sufficiently accurate for use in the community-dwelling population. Select cardiovascular variables measured by six of these RMTs were sufficiently precise. Due to lacking data, no precision outcomes were available for the three remaining RMTs (Spacelabs 90207 BPM, Fitbit Charge 3, and Omron HEM-9200 T BPM). Moreover, the accuracy and precision depend on the sensor and the algorithm that processes the raw signal [6]. Changes in the processing algorithm of the Polar H10 HR Sensor affected accuracy for HR from a bias of –0.32 beats per minute (bpm) to –0.97 bpm and for HRV from a bias of –6.98 ms (ms) to –0.74 ms [15]. For precision, the change in algorithm decreased the correlation to the reference standard HRV measurement from close to 1 to around 75 percent. The processing algorithms of many RMTs, both research-grade and commercial devices, are proprietary [46] (e.g., AliveCor, Omron, Polar). Rapid obsolescence is a characteristic of the technological lifecycle, and it is inevitable that hardware and software will constantly be upgraded and redesigned to new models [47]. The majority of the reported RMTs are commercial devices; thus, time and resources must be allocated for the validation of each iteration of the RMT (hardware and software) to ensure accuracy and precision of the device for use in the intended populations.

Over half of the included articles studied populations with a mean/median age over 50 years, indicating that RMTs are pertinent for ageing research. Ageing is one of the main risk factors for chronic diseases, such as cardiovascular disease, diabetes, and cancer [48]. The ageing population is growing rapidly in many developed countries. Consequently, the prevalence of age-related chronic diseases increases, accompanied by an exponential rise in healthcare costs [38]. These circumstances stress the importance of healthy ageing, which can be supported with effective disease prevention and monitoring methods that delay ageing and disease progression. As RMTs become increasingly advanced and comfortable to wear, they are valuable tools for monitoring health status continuously and consistently. This systematic review could be used to select RMTs for use not only in healthcare settings but also in ageing interventions and research.

A strength of this review is that it provides a broad overview of available devices for measuring cardiovascular variables to clinicians and researchers with various objectives. This systematic review focuses on the cardiovascular system regardless of health status or indication for monitoring. In contrast, previous reviews focused on a specific type of device [49], a specific disease or symptom [2, 5], or a specific cardiovascular function [50].

One limitation of this review is the inclusion of articles with a limited publication time window due to the exponential rise in publications relating to RMTs. Machine learning algorithms might be an option to carry out the screening process in the future [51]. Contrarily, technologies are evolving rapidly; therefore, RMTs in articles published before 2020 may not remain relevant. Another limitation is the complication of comparing the accuracy and precision between the RMTs due to the different validation methods used; thus, device accuracy and precision under one set of conditions used for validation may not remain the same under different conditions (e.g., change in study population, validation measure, etc.) [46].

This systematic review identified RMTs that aim to measure cardiovascular functions in community-dwelling adults. The presented usage, accuracy, and precision of the RMTs can aid healthcare professionals, researchers, and consumers in selecting a suitable device for their various purposes.

## Supplementary Information

Below is the link to the electronic supplementary material.

Supplementary file1 (PDF 851 KB)

## Data Availability

All data for this review were obtained from published primary articles. Data from brand/company websites are cited in references. Data extracted for this review and database search strategies will be made available on request. For access, please email the corresponding author.
