# Supplementary material for: Remote monitoring technologies for measuring cardiovascular functions in community-dwelling adults: a systematic review
Source: GeroScience. 2023 May 18;45(5):2939–50. doi: 10.1007/s11357-023-00815-4 (PMC10196312; doi:10.1007/s11357-023-00815-4)
Supplement: Supplementary file 1 — Supplementary file1 (PDF 851 KB) [file 11357_2023_815_MOESM1_ESM.pdf]

SUPPLEMENTARY MATERIAL

Remote monitoring technologies for measuring cardiovascular functions in community-dwelling adults: A Systematic Review

Jessica K. Lu[1,2], Marcella Sijm[3], Georges E. Janssens[4], Jorming Goh[1,2,5], Andrea B. Maier[1,2,6]

[1]Centre for Healthy Longevity, National University Health System, Singapore.

[2]Healthy Longevity Translational Research Program, Yong Loo Lin School of Medicine, National University of Singapore, Singapore.

[3]University of Amsterdam, Amsterdam, The Netherlands.

[4]Laboratory Genetic Metabolic Diseases, Amsterdam University Medical Centers – location Academic Medical Center, University of Amsterdam, Amsterdam, The Netherlands.

[5]Department of Physiology, Yong Loo Lin School of Medicine, National University of Singapore, Singapore.

[6]Department of Human Movement Sciences, Vrije Universiteit Amsterdam, Amsterdam, The Netherlands.

Corresponding author:

Prof Dr Dr Andrea B Maier

@Age, Department of Human Movement Sciences

Faculty of Behavioural and Movement Sciences

Vrije Universiteit Amsterdam

Amsterdam Movement Sciences

Van der Boechorstsraat 7, 1081 BT Amsterdam

The Netherlands

Email: **a.b.maier@vu.nl**

Contents

Appendix 1. Search strategy (PubMed example shown). **3**

Appendix 2. Adapted version of the Newcastle-Ottawa Scale for study quality assessment. **4**

Appendix 3. Study characteristics of the 272 included articles reporting the use of remote monitoring technologies (RMTs) measuring cardiovascular variables in community-dwelling adults. **5**

Appendix 4. Characteristics of the remote monitoring technologies (RMTs) measuring cardiovascular variables in community-dwelling adults described in the 272 included articles. **12**

Appendix 5. Summary of the cardiovascular variables measured by 216 distinct remote monitoring technologies reported in the 272 included articles. **25**

Appendix 6. Newcastle-Ottawa Scale quality assessment for observational studies. **31**

Appendix 7. Cochrane risk of bias summary per study for randomised controlled trials. **32**

Appendix 8. Risk Of Bias In Non-randomised Studies - of Interventions (ROBINS-I) summary per study. **33**

Appendix 9. Association between the characteristics of the remote monitoring technologies used and the study quality. **34**

References **35**

Appendix 1. Search strategy (PubMed example shown).

Concept	#	PUBMED Query	Results
Digital health	1	Telemedicine[MeSH] OR "Wearable Electronic Devices"[MesH] OR telemedicine[tiab] OR wearable*[tiab] OR (remote[tiab] AND (monitor*[tiab] OR technolog*[tiab])) OR "electronic health*"[tiab] OR ehealth[tiab] OR "e-health"[tiab] OR "mobile health*"[tiab] OR mhealth[tiab] OR "m-health"[tiab] OR "digital health*"[tiab] OR telehealth[tiab] OR smartphone*[tiab] OR "mobile phone*"[tiab] OR smartwatch*[tiab] OR watch*[tiab] OR "information technology"[tiab]	189,708
Monitoring	2	"Monitoring, Physiologic"[MeSH] OR "Monitoring, Ambulatory"[MeSH] OR "Biological Monitoring"[MeSH] OR monitor*[tiab] OR measur*[tiab] OR "health management"[tiab] OR telemonitor*[tiab] OR telemetr*[tiab] OR "self monitor*"[tiab] OR selfmonitor*[tiab] OR "self-monitor*"[tiab] OR "clinical alarm*"[tiab] OR biosens*[tiab] OR sensor*[tiab]	4,938,705
Physiological variables and cardiovascular system	3	"Cardiovascular Physiological Phenomena"[MeSH] OR cardiovascular[tiab] OR cardiac[tiab] OR cardio*[tiab] OR heart[tiab] OR vascular[tiab] OR ventric*[tiab] OR "blood vessel*"[tiab] OR "hemodynamic monitor*"[tiab] OR "blood pressure*"[tiab] OR sphygmomanometer*[tiab] OR "heart rate"[tiab] OR heartrate[tiab] OR electrocardio*[tiab] OR ECG[tiab] OR pulse*[tiab] OR oxygen[tiab] OR "stroke volume*"[tiab] OR "flow-mediated dilation"[tiab]	3,729,126
Population	4	Human*[tiab] OR independen*[tiab] OR “community-dwelling”[tiab] OR “community-based”[tiab] OR “home-based”[tiab] OR patient*[tiab] OR outpatient*[tiab] OR adult*[tiab] OR elder*[tiab] OR eldest[tiab] OR geriatri*[tiab] OR geroscience[tiab] OR "old age*"[tiab] OR "oldest old*"[tiab] OR senior*[tiab] OR senium[tiab] OR "very old*"[tiab] OR septuagenarian*[tiab] OR octagenarian*[tiab] OR octogenarian*[tiab] OR nonagenarian*[tiab] OR centarian*[tiab] OR centenarian*[tiab] OR supercentenarian*[tiab] OR "older people"[tiab] OR "older subject*"[tiab] OR "older patient*"[tiab] OR "older age*"[tiab] OR "older adult*"[tiab] OR "older man"[tiab] OR "older men"[tiab] OR "older male*"[tiab] OR "older woman"[tiab] OR "older women"[tiab] OR "older female*"[tiab] OR "older population*"[tiab] OR "older person*"[tiab]	11,692,318
TOTAL	5	#1 AND #2 AND #3 AND #4 including filters for text availability (full text), language (English), and results by year (2020–2022).	3,195

Appendix 2. Adapted version of the Newcastle-Ottawa Scale for study quality assessment.

Further adapted from Amaral Gomes ES, Ramsey KA, Rojer AGM, Reijnierse EM, Maier AB. The Association of Objectively Measured Physical Activity and Sedentary Behavior with (Instrumental) Activities of Daily Living in Community-Dwelling Older Adults: A Systematic Review. *Clin Interv Aging* 2021;**16**:1877–915.

Newcastle-Ottawa Scale (NOS)

The NOS was customized for cross-sectional and longitudinal studies in this systematic review using the same questions in each domain (selection, comparability, and outcome), with two additional outcome criteria for studies with a longitudinal study design. For cross-sectional studies (maximum of 7 stars), a score greater than or equal to four identified high-quality studies, whereas a score lower than four denoted low-quality studies. For longitudinal studies (maximum of 9 stars) a score greater than or equal to five identified studies of high quality, whereas a score lower than five denoted studies of low quality. (★ = 1 star).

Note: study quality was also assessed for articles that studied/used remote monitoring technologies (independent variable) that measured cardiovascular function(s) (dependent variable).

Selection (maximum of 3 stars)

1. Representativeness of study population
    a. Truly representative sample of which the age, sex distribution, countries, and health status of the community-dwelling adult population are reported ★
    b. Not representative based on aforementioned factors or no description
2. Ascertainment of exposure: remote monitoring technology to measure/for measuring cardiovascular function(s)
    a. Clearly described by name and model of device, device wearing location, and cardiovascular variable measured ★
    b. Methodological criteria of data capturing/processing are clearly described based on the following information: automatic upload of data to server(s) accessible by researcher(s) ★
    c. Non-automized upload of data
    d. No description

Comparability (maximum of 3 stars)

3. Comparability of cohorts based on study design and statistical analysis
    a. The study controls for the most important factors, age and sex, for at least one association ★
    b. The study adjusted for any other factors, such as population characteristics (e.g., health status) or lifestyle ★
    c. No adjustment or not described
4. Statistical analysis: method(s)/test(s) to quantify the association(s) of interest
    a. The performed statistical analysis was clearly described, and measurement of the association is clearly presented, including the effect size with (95%) confidence intervals, the probability level (*p-value(s)*), or standard error for at least 1 association ★
    b. The statistical test was incomplete or not described
    c. Not applicable (reduce one ★ for maximum number of stars)

Outcome (for cross-sectional studies, a maximum of 1 star; for longitudinal studies, a maximum of 3 stars)

5. Assessment of outcome: cardiovascular function measurements were collected and reported
    a. Clear description of established/validated method or algorithm that was used to assess cardiovascular function measurements ★
    b. No description
6. *(Only for longitudinal studies)* Was follow-up long enough for outcome change to occur?
    a. Yes ★
    b. No
7. *(Only for longitudinal studies)* Adequacy of follow-up
    a. Complete follow-up with all participants accounted for or only a small number lost (<20%) ★
    b. Large number of participants lost (≥20%)
    c. No description

Appendix 3. Study characteristics of the 272 included articles reporting the use of remote monitoring technologies (RMTs) measuring cardiovascular variables in community-dwelling adults.

First author (year)	Country	Study design	Age, years	Sample size, N	Females, n	Population characteristics
Agyün (2021)[1]	Turkey	O, CX	21.3 (2.2)	43	21	Healthy, physically active
Ahn (2021)[2]	South Korea	O, CX	66.9 (9.1)	56	23	Parkinson's disease
Akbar (2021)[3]	USA	O, L	43.8 (9.5)	47	32	Physicians without cardiac medications, pacemakers or defibrillators, and arrhythmias
Al Rajeh (2020)[4]	UK	I, RCT	70.6 (8.1)	83	43	COPD outpatients
Al-Kaisey (2020)[5]	Australia	O, L	68 (12)	32	12	24-hour Holter monitoring
Al-Naami (2021)[6]	Jordan	O, CX	{22–33}	13	—	Normal
Alexandrou (2020)[7]	Greece	O, L	62.2 (13.1)	114	45	Peritoneal dialysis; Hemodialysis
Aljuaid (2020)[8]	USA	O, R	66.2 (11)	90	36	Atrial fibrillation ablation
Amelard (2021)[9]	Canada	O, CX	26 (5)	22	9	Healthy
Angelucci (2021)[10]	Italy	O, L	43.7	18	11	Healthy
Antali (2021)[11]	Hungary	O, CX	39.8 (16.7)	45	26	Healthy; Type 2 diabetes
Atkins (2020)[12]	Italy	O, CX	72.3 (14.2)	85	51	Hypertensive; Normotensive
Atzmon (2020)[13]	Israel	O, CX	30* {21–42}	81	81	Healthy, singleton pregnancy
Avram (2021)[14]	USA	O, L	62.6 (11.6)	204	95	Self-reported atrial fibrillation diagnosis
Baek (2021)[15]	South Korea	O, CX	23.7 (3.0)	15	0	Healthy
Bartlett (2021)[16]	USA	O, L	64 [56, 71]<br>44 [39, 57]	59	34	Atrial fibrillation ablation<br>Bariatric surgery patients
Batalik (2021)[17]	Czech Republic	O, L	56.6 (7.3)	44	8	Coronary heart disease
Batta (2022)[18]	Hungary	O, CX, L	48.3 (13.2)	105	43	Hypertension
Beers (2021)[19]	Netherlands	O, CX	61 (14)	125	62	12-lead ECG monitoring
Beime (2021)[20]	Germany	O, CX	56.3 (16.3)	85	54	No arrhythmia
Benedetti (2021)[21]	Italy	O, CX	22.4 (3.0)	25	17	Undergraduate and graduate students
Berryhill (2020)[22]	USA	I, RCT	23.8 (5)	28	21	Healthy
Blok (2021)[23]	Netherlands	O, CX	60 (15)	180	80	Cardiac outpatients
Böhm (2020)[24]	Spain	O, R	58.3 (13.8)	56900	26585	Hypertension
Bolin (2022)[25]	USA	I, L	22.7 (4.3)	34	26	Overall excellent to good health
Bourassa (2020)[26]	Canada	O, CX	51.3 (5.3)	28	14	Manual wheelchair users
Browne (2021)[27]	USA	O, CX	—	12	8	Healthy, darkly pigmented skin
Browne (2021)[28]	USA	O, CX	46.1 (13.1)	250	59	Outpatients
Budig (2021)[29]	Germany	O, CX	36.1 (12.8)	36	16	Moderate endurance-trained
Burkhoff (2020)[30]	USA	O, L	58 (13)	1066	245	Heart failure (73% ischaemic)
Cao (2022)[31]	Finland	O, CX	32.3 (6.4)	35	19	Healthy
Capdevila (2021)[32]	Spain	O, CX	47.4 (1.5)	121	70	Healthy controls; Myalgic encephalomyelitis/ chronic fatigue syndrome outpatients
Carnagarin (2022)[33]	Australia	O, L	62.6 (10.2)	153	58	Resistant or uncontrolled hypertension
Castillo-Escario (2021)[34]	Spain	O, L	40 (15)	38	—	Healthy controls; Spinal cord injury patients
Chalmers (2021)[35]	Australia	O, CX	28.9 (8.8)	60	27	Medical students; General population
Chang (2020)[36]	Taiwan	O, CX	21.4 (1.8)	120	58	Athletes; Non-athletes; Sedentary; Exercise-habit
Chaudhury (2021)[37]	USA	O, L	{18–50}	18	—	Healthy
Chen (2020)[38]	China	O, CX	63.5 (14.7)	401	197	Normal controls; ECG-diagnosed AF
Chen (2020)[39]	Taiwan	O, CX	22.3 (3.0)	60	—	Healthy
Chesebro (2020)[40]	Venezuela	O, L	59.4 (12.9)	435	316	Hypertension; Normotension
Chiang (2021)[41]	USA	O, L	50.2 (14.3)	25	9	Elevated blood pressure or stage I hypertension

First author (year)	Country	Study design	Age, years	Sample size, N	Females, n	Population characteristics
		I, RCT	52.2 (13.3)	13	9	
Cho (2022)[42]	USA	O, L	—	117	—	Ambulatory ECG monitoring
Chorin (2021)[43]	Israel	O, L	59.4 (21.3)	18	5	Ventricular tachycardia/fibrillation induction or Transient ventricular asystole provocation
Chow (2020)[44]	Taiwan	O, CX	{20–26}, {>65}	40	—	No cardiovascular diseases, neurological disorders, and lower limb injuries
Christen (2020)[45]	USA	I, L	37 (12)	62	38	Healthy
Climstein (2020)[46]	Australia	O, CX	26.1 (3.4)	29	13	Healthy
Cooper (2020)[47]	USA	O, L	69.7 (7.2)	17	12	Moderate to severe COPD
Cos (2021)[48]	USA	O, L	63.2 (11.6)	48	29	Scheduled for pancreatectomy
Costa (2021)[49]	Italy	I, RCT	57.5 (5.7)	32	17	Newly diagnosed, never treated, mild hypertensive outpatients
Cuspidi (2020)[50]	Italy	O, R	61.8 (14.6)	7353	4463	Untreated/treated hypertensive outpatients
Dagel (2020)[51]	Turkey	O, CX	25.4 (5.0)	100	63	Healthy, normotension
Davidson (2020) [52]	Germany	O, L	29.4 (7.0)	12	0	Trained and untrained/recreational runners
de la Casa Pérez (2022)[53]	Spain	O, CX	38.7 (14.4)	46	19	No physical limitation, no treatment that disrupts cognitive and physical functions
de la Sierra (2021)[54]	Spain	O, R	61.8 (12.9)	38188	16274	Hypertension
Degott (2021)[55]	Switzerland	O, CX	52.9 (15.9)	91	49	Hypertensive outpatients
Dehghani Zahedani (2021)[56]	USA	O, L	37.7 (9.7)	665	190	Healthy; Pre-diabetes; Diabetes
Dinesen (2021) [57]	Denmark	O, L	70.6 (5.2)	20	7	Atrial fibrillation
Dörr (2021)[58]	Switzerland	O, CX	61.0 (18.9)	965	477	Outpatients
Dunietz (2020)[59]	Israel	O, L	33 (4)	148	148	Singleton pregnancy
Edwards (2020)[60]	Ireland, USA	O, L	62 {31–79}	27	13	Self-reported pulmonary fibrosis
Elzinga (2021)[61]	Netherlands	O, L	64.3 (6.6)	12	2	Parkinson’s disease
Fanget (2022)[62]	France	I, L	61.5 (8.7)	54	10	Acute coronary syndrome/myocardial infarction
Fouassier (2020)[63]	France	O, CX	29.5 (7.8)	30	9	Healthy
Fu (2022)[64]	Canada	O, L	43.6 (11.6)	38	24	—
Gambassi (2020)[65]	Brazil	O, CX	24.9 (3.4)	15	0	Asymptomatic
Garcia-Moreno (2022)[66]	Spain	O, CX	75 {65–90}	79	69	Older adults from community day centres
Gazit (2021)[67]	USA	O, L	51 [43, 58]	28189	9424	Hypertension
Georgianos (2021)[68]	Greece	O, L	62.8 (15.8)	108	38	End-stage renal disease, long-term peritoneal dialysis
Gielen (2021)[69]	USA	O, R	{24–49}	2	0	SARS-CoV-2-positive
Girerd (2022)[70]	France	O, L	56.3 (12.8)	2273	430	Persons living in the Paris urban area
Giudicessi (2021)[71]	USA	O, L	28.7 (18.5)	686	390	Patients referred to the Heart Rhythm Clinic
Gkikopoulos (2022)[72]	Switzerland	O, R	57	1	1	Giant cell arteritis
Gladstone (2021)[73]	Canada, Germany	I, RCT	80.0 (4.0)	856	487	Hypertension
Gognieva (2022)[74]	Russia	O, L	56 (12.8)	3249	2122	—
Golbus (2021)[75]	USA	O, L	48 [33, 61]	6454	3482	Hypertensive outpatients
Goldstein (2021)[76]	Belgium	O, L	40.2 (10.5)	116	67	Healthy, received novel influenza vaccine
Gordon (2021)[77]	USA	O, L	{18–90}	21923	4487	MyBPLab Google Playstore application users
Gordon (2020)[78]	USA	O, L	54 [41, 65]	225	114	RPM after COVID-19 hospital discharge
Goudman (2021)[79]	Belgium	O, CX	55.1 (7.6)	22	16	Failed back surgery syndrome patients treated with spinal cord stimulation
Gräfitsch (2020)[80]	Switzerland	O, L	48.8 (16.3)	16	5	Abdominal wall hernia
Gresham (2021)[81]	USA	I, L	67.0 (9.3)	16	9	Pancreatic ductal adenocarcinoma, cachexia
Hahnen (2020) [82]	USA	O, CX	53 (21)	85	36	Pre-hospital admission testing
Haveman (2022)[83]	Netherlands	O, CX	64* {20–74}	20	9	General patient population
Hermans (2021)[84]	Netherlands	O, L	64* {58.0–68.0}	115	35	Paroxysmal atrial fibrillation ablation

First author (year)	Country	Study design	Age, years	Sample size, N	Females, n	Population characteristics
Hillmann (2021)[85]	Germany	O, R	57.4 (15.3)	276	88	Heart failure, reduced ejection fraction
Hochstadt (2020)[86]	Israel	O, L	41.3 (18.6)	24	17	Ambulatory
Hofstede (2022)[87]	Netherlands	O, CX	32.6 (4.7)	63	63	Pregnant outpatients
Hojjatinia (2021)[88]	USA	O, L	42.6 (14)	45	22	Smokers
Holgado (2021)[89]	Spain	O, CX	23.5 (6.3)	30	6	Recreationally active, regular aerobic training
Holmes (2020)[90]	USA	O, L	23.9 (5.4)	31	9	Healthy, resistance-trained
Holmner (2020)[91]	Sweden	O, L	67 [59, 72]	13	8	Moderate to very severe COPD
Holyoke (2021)[92]	Canada	O, CX	43.5 (11.2)	62	49	>25 years old, working in an office building
Huang (2021)[93]	Taiwan	O, R	70.3 (13.8)	253	86	Chronic cardiovascular diseases
Huang (2020)[94]	Belgium	O, CX<br>I, RCT	29 {22–38}<br>46 {19–60}	18	0	Healthy
Huppertz (2020)[95]	UK	O, L	71.6 (10.9)	48	20	Sick sinus syndrome, AF or atrial high-rate episodes
Hutchings (2021)[96]	Australia	O, L	38* {11–79}	162	—	COVID-19 patients receiving virtual health care monitoring
Huynh (2021)[97]	USA	O, CX	66.0 (6.5)	20	3	Obstructive sleep apnea, high risk for AF
Ingvaldsen (2021)[98]	Norway	O, L	40.6 (9.8)	18	17	Headache clinic outpatients
Inui (2020)[99]	Japan	O, L	70.9 (11.1)	40	13	Cardiac surgery, diagnosed postoperative AF
Ioachimescu (2020)[100]	USA	O, CX	52.5 [41.8, 62.5]	500	100	Military veterans with obstructive sleep apnea
Ionov (2021)[101]	Russia	I, RCT	47 {21–41}	160	65	Patients with uncontrolled hypertension
Iqbal (2021)[102]	UK	O, L	34.9 (11.0)	14	7	Suspected COVID-19 infection, isolation in hotels
Jachymek (2021)[103]	Poland	O, CX	28 {18–70}	31	10	Healthy
Jen (2020) [104]	USA	O, L	63 (7)	33	13	Established COPD
Jones (2021) [105]	UK	O, CX	65.3 (13.6)	49	17	Major elective intra-abdominal surgery
Jortveit (2022) [106]	Norway	O, L	48 (14)	97	46	—
Kaile (2021)[107]	USA	O, CX	—	3	—	Controls without any known pre-existing conditions
Kalanadhabhatta (2021)[108]	USA	O, L	28* {20–42}	24	14	Shift workers; regular workers; graduate students
Kanady (2020)[109]	USA	O, CX	26.8 (3.4)	18	13	Clinically healthy, medication-free
Kańtoch (2021)[110]	Poland	O, R	57 (22.4)	5	3	Working; Octogenarians
Kario (2021)[111]	Japan	I, RCT	52.2 (7.9)	390	78	Diagnosed essential hypertension
Kario (2021)[112]	Japan	I, RCT	56.8 (9.2)	146	48	Patients with essential hypertension
Kario (2020)[113]	Japan	O, L	66.1 (10.8)	50	20	Hypertensive outpatients
Khattak (2021)[114]	Pakistan	O, CX	37.3 (14.8)	126	53	Healthy
Khushhal (2020)[115]	UK	O, L	67.0 (10.0)	30	5	Patients with a history of cardiac disease
Kilic (2020)[116]	Turkey	O, R	50.2 (14.6)	2108	1360	Patients with high risk of early vascular aging syndrome
Kim (2020)[117]	South Korea	I, RCT	58.1 (11.2)	60	21	Acute ischemic stroke patients
Kim (2021)[118]	Canada	O, L	66.8 (6.8)	12	7	Frail, non-frail
Kim (2020) [119]	Canada	O, L	82.3 (10.8)	37	28	Home care clients
Kim (2020)[120]	South Korea	I, RCT	25.0	25	15	Difficulty initiating or maintaining nighttime sleep
Kim (2021) [121]	South Korea	O, L	39.5 (8.3)	44	27	Total thyroidectomy and radioactive iodine therapy for differentiated thyroid cancer
Kim (2020) [122]	South Korea	O, L	37	1	—	Obese with a body mass index > 25 kg/m^2
Kim (2020) [123]	South Korea	O, L	56.7 (7.7)	31	5	Hepatocellular carcinoma
Kinnunen (2020)[124]	Finland	O, CX	31.6 (11.8)	60	40	Apparently healthy
Ko (2022)[125]	Taiwan	O, L	62.0 (9.3)	57	28	Healthy controls; Parkinson's disease
Koh (2021)[126]	Malaysia	I, RCT	65.5 (7.8)	203	51	Cryptogenic stroke or transient ischaemic attack
Kokubo (2020)[127]	Japan	O, L	61.7 (11.6)	94	20	65% hypertension; 33% sleep apnea syndrome
Kolkenbeck-Ruh (2022)[128]	South Africa	O, CX	29.0* {29.0–55.0}	85	85	1/3 of sample had hypertension with 82% on high cholesterol treatment

First author (year)	Country	Study design	Age, years	Sample size, N	Females, n	Population characteristics
Krhut (2021)[129]	Czech Republic	I, RCT	43.7	66	17	Spinal cord injury and multiple sclerosis-induced neurogenic detrusor overactivity
Kuula (2021)[130]	Finland	O, CX	24.5 (3.5)	20	10	Healthy (no diagnosed sleep disorder)
Kwon (2021)[131]	South Korea	O, L	55.1 (12.8)	29	15	Non-AF patients with pre-diagnosed arrythmias
Kwon (2021)[132]	USA	O, CX	32.5 (15.1)	20	12	Healthy Pacific Islanders
Lai (2021)[133]	USA	O, L	—	1	0	Asian-American student in college sophomore year
Lambert (2021)[134]	USA	I, RCT	64.0 (10.2)	99	29	Previous successful AF ablation
Lan (2020)[135]	Taiwan	O, CX	57.5 (17.4)	80	42	Normal (healthy); Wiry pulse (has liver disease)
Larsen (2022)[136]	Denmark	I, L	48 (13)	50	42	Inactive
Lauder (2020)[137]	Germany	O, R	66.7 (14.1)	1500	504	Cardiology outpatients, Holter monitoring, ABPM
Layton (2021)[138]	USA	I, L	33 (7)	11	5	Lung transplant candidates with cystic fibrosis
Li (2021)[139]	China	I, RCT	48.2 (10.4)	101	24	Type 2 diabetes without severe complications or comorbidities
Liu (2022)[140]	USA	I, RCT	47.1 {22–84}	51	31	Exposure to a COVID-19-infected individual
Liu (2020)[141]	Hong Kong	O, L	{61–91}	14	12	Hypertension, high cholesterol, and/or Type 2 diabetes
Liu (2022)[142]	China	O, L	52.6 (12.2)	366	159	Thrice weekly maintenance hemodialysis patients
Liu (2022)[143]	China	O, L	73.1 (7.6)	100	45	Severe aortic stenosis, elective transfemoral TAVR
Lo (2020)[144]	Taiwan	O, CX	33 [31, 40]	120	54	Manufacturing workers working 12-hour shifts
Low (2021)[145]	USA	O, L	65.7 {40–82}	44	18	Scheduled for pancreatic surgery
Lumikari (2020)[146]	Finland	O, L	59.5 (7.4)	15	5	Embolic stroke of unknown source
Luštrek (2021)[147]	Belgium, Italy	I, RCT	—	56	—	Healthy
Mach (2020)[148]	Austria	O, L	77.5 (5.1)	50	22	Undergoing transfemoral or transapical TAVR
Magnusson (2020)[149]	Sweden	O, L	67.6 (10.8)	100	40	Clinically diagnosed ischaemic (cryptogenic) stroke
Maille (2021)[150]	France	O, L	38.3 (12.2)	85	55	PCR-positive SARS-CoV-2 infection
Marcelli (2021)[151]	Italy	O, CX	39 (9)	5	3	Healthy
Marcus (2022)[152]	USA	I, RCT	58 (14)	499	210	Symptomatic patients with AF or increased risk(s) of AF events
Martini (2022)[153]	Italy	O, CX	51	1	0	Healthy, occasional premature heartbeats
Mason (2022)[154]	USA	O, L	51.0 (11.5)	1179	625	Received COVID-19 vaccination
McManus (2021)[155]	UK	I, RCT	66.0 (10.3)	623	288	Treated but poorly controlled hypertension
McNeil (2022)[156]	Canada	I, RCT	58.5 (9.3)	45	45	Histologically confirmed stage I–IIIc breast cancer
Mena (2020)[157]	Mexico	O, CX	61.3 (1.5)	3	2	No history of cardiovascular disease or hypertension
Meng (2020)[158]	USA	O, L	—	182	—	Stable ischemic heart disease
Mhajna (2020)[159]	Israel, USA	O, CX	31.8 (6.9)	147	147	Singleton pregnancies ≥ 32 weeks of gestation
Miller (2020)[160]	Australia	O, L	22.9 (3.4)	12	6	Healthy
Miranda Hurtado (2021)[161]	Chile	O, CX	38.9 (12.2)	33	19	Normotension; Hypertension
Mishra (2020)[162]	USA	O, CX	23 (3.2)	27	15	Undergraduate and graduate students
Mocny-Pachońska (2020)[163]	Poland	O, CX	—	60	—	Junior, mid-senior, and senior students of the Dental Division at the Silesian Medical University
Modi (2021)[164]	USA	O, L	69 [57, 76]	47	36	Outpatient pulmonary function test
Moitinho-Silva (2021)[165]	Germany	I, RCT	31.1 (8.4)	49	31.11	Controls, no exercise; Healthy, physically inactive
Montrivade (2020)[166]	Thailand	O, L	58 (12.7)	1184	695	Hypertension
Moon (2020)[167]	South Korea	O, CX	57.1 (17.9)	35	18	Outpatient clinic visit (nine has hypertension, four has dyslipidemia)
Morgado Areia (2021)[168]	UK	O, L	32.5 (10.5)	29	16	Healthy
Moshe (2021)[169]	Finland, Germany, USA	O, L	42.8 (11.60)	55	30	Owned an Oura Ring
Motta (2021)[170]	Brazil	O, L	37.7 (13.9)	24	17	Healthy controls; Asymptomatic to mild COVID-19 symptoms
Mugabirwe (2021)[171]	Uganda	O, L	58 (10.8)	37	28	Hypertension, receiving clinical care
Muggeridge (2021)[172]	UK	O, CX	40 (10)	20	9	Healthy
Murase (2022)[173]	Japan	O, L	58 (12)	6568	4394	Nagahama city residents

First author (year)	Country	Study design	Age, years	Sample size, N	Females, n	Population characteristics
Nabasny (2022)[174]	USA	O, L	42.4 (13.8)	64	44	With and without traumatic brain injury and/or loss of consciousness
Nissen (2022)[175]	Germany	O, CX	24.2 (4.6)	23	10	Healthy
Nolde (2021)[176]	Australia	O, L	56.7 (17.1)	222	92	Treated hypertension
Ocagli (2021)[177]	Italy	O, L	79 [78, 83]	17	10	Surgical/transcatheter aortic valve replacement
O'Driscoll (2020)[178]	UK	O, CX	44.4 (14.1)	59	41	Healthy
Oh (2022)[179]	South Korea	I, RCT	56.88 (6.66)	32	9	Obesity, hypertension, Type 2 diabetes mellitus
Ohtsuka (2021)[180]	Japan	I, L	52.7 (11.9)	32	11	Obstructive sleep apnea syndrome/mild/moderate sleep apnea
Olbers (2021)[181]	Sweden	O, L	68.4 (8.0)	98	22	Persistent atrial fibrillation
Omboni (2020)[182]	Argentina, Australia, Italy, Kazakhstan, Mexico, Portugal, Romania, Russia	O, L	51.6 (15.8)	646	297	Hypertension-mediated organ damage (cardiac, vascular, renal)
Ong (2021)[183]	Singapore	O, L	30.9 (4.6)	1824	941	Office workers in Singapore
Panula (2020)[184]	Finland	O, CX	32 {23–75}	33	8	4 on blood pressure medication
Park (2022) [185]	USA	O, CX	70.6 (7.2)	79	31	Stable chronic obstructive pulmonary disease
Pavic (2020)[186]	Switzerland	O, L	64 [53.0, 71.0]	31	9	Cancer patients in palliative care (estimated life expectancy >8 weeks to <12 months)
Persell (2020)[187]	USA	I, RCT	58.9 (12.8)	297	182	Uncontrolled hypertension
Pinheiro (2020)[188]	Brazil	O, CX	55.3 (13.8)	304	135	Suspected obstructive sleep apnea
Pipek (2021)[189]	Brazil	O, CX	60.0 (4.3)	100	65	COPD, interstitial lung disease outpatients
Polverino (2022)[190]	Italy	I, L	77.9 (5.2)	8	3	Idiopathic Parkinson's disease, orthostatic hypotension
Porumb (2020)[191]	UK	O, L	30.8 (3.5)	4	2	Healthy
Praus (2021)[192]	USA	O, L	66.8 (9.8)	43	20	AF-related emergency department/urgent care visits
Prigent (2021)[193]	Switzerland	O, CX	34 (10)	31	9	Healthy
Puhr-Westerheide (2020)[194]	Germany	O, CX	—	—	0	Young and healthy athletic kart-drivers
Quer (2020)[195]	USA	O, R	45.8 (14.4)	92457	57836	Body mass index between 15 and 50 kg/m^2
Rodrigues (2022)[196]	Brazil	O, CX	—	10	—	Active, without chronic-degenerative diseases and neurofunctional disorders
Roe (2020)[197]	USA	O, L	64.8 {55–77}	11	5	Healthy, residents in independent living facility for older people
Rykov (2021)[198]	Singapore	O, CX	33 (8.6)	290	170	Healthy, working
Rykov (2020)[199]	Singapore	O, CX	44.3 (12)	83	19	Healthy, working
Saarikko (2020)[200]	Finland	O, L	26 (5.0)	20	20	Nulliparous, postpartum
Saghir (2020)[201]	USA	O, CX	31 (8.5)	43	28	Healthy
Sakulsupsiri (2021)[202]	Thailand	O, L	58 (12.3)	1177	699	Hypertension without prior regular HBPM
Salazar (2020)[203]	Argentina	O, L	51.4 (14.4)	1344	797	Hypertension, ambulatory blood pressure monitoring
Salvi (2021)[204]	UK	O, L	50 (16.6)	30	19	Pulmonary arterial hypertension
Saner (2020)[205]	Switzerland	O, L	88.9 (7.5)	24	19	History of chronic disease
Sardana (2021)[206]	USA	O, L	53 (9)	660	387	Electronic Framingham Heart Study participants
Sarganas (2020)[207]	Germany	O, CX	{21–86}	124	72	Hypertensive outpatients; White collar workers
Sasaki-Otomaru (2020)[208]	Japan	O, CX	54.2 {24–81}	68	38	Elderly with no dementia diagnosis; Manufacturing company employees with no heart diseases
Sato (2021)[209]	Japan	O, L	62.1 (14.2)	62	35	Asthma
Savvari (2020)[210]	Greece	O, L	66.5 (10)	2408	1235	Arterial hypertension
Sayer (2022)[211]	USA	O, L	60.7 (15.2)	34	15	Healthy; Unspecified hospitalization
Schubert (2020)[212]	Germany, Netherlands, UK	O, CX	66 [56, 75]	107	42	Aortic stenosis, mitral regurgitation, and mixed valvular heart disease

First author (year)	Country	Study design	Age, years	Sample size, N	Females, n	Population characteristics
Schwartz (2020)[213]	USA	O, L	41.2 (13.1)	400	238	Free of known cardiovascular disease
Selder (2020)[214]	Belgium	O, CX	69.6 (16.9)	60	41	Senior care, hypertension, hyperlipidemia
Sequeira (2020)[215]	Canada	O, CX	52.3 (17.2)	65	35	Re-entrant supraventricular tachycardia
Shan (2020)[216]	USA	O, L	58 (10)	68	17	Discharged from acute myocardial infarction
Sher (2022)[217]	USA	O, L	61* {54–69}	43	6	Head and neck cancer radiotherapy patients
Shu (2020)[218]	China	O, CX	23.5 {22–25}	25	13	Good health
Shufelt (2020)[219]	USA	O, L	65 (11)	198	119	Stable ischaemic heart disease
Shui (2021)[220]	China	O, L	21.5 {18–31}	142	78	—
Shumate (2021)[221]	Australia	O, CX	26.1 (3.4)	30	14	Healthy
Sjöberg (2021)[222]	Sweden	O, CX	43.8 (11.8)	41	31	Chronic musculoskeletal or widespread pain
Slade (2021)[223]	USA	O, L	65 [61, 74]	20	2	Radical cystectomy for bladder cancer
So (2021)[224]	Hong Kong	O, CX	38.1 (14.0)	21	12	—
Sokas (2021)[225]	Lithuania	O, L	39.8* {19–68}	99	63	Cardiovascular disease; Healthy and below 40-years-old
Sola (2021)[226]	Switzerland	O, CX	38 (13)	91	—	Low to hypertensive blood pressure levels
Song (2020)[227]	China	O, CX	60.2 (13.5)	33	17	Healthy; Hypertension
Spaccarotella (2022)[228]	Italy	O, CX	64.0 (16.2)	257	89	Healthy; Lung disease; Cardiovascular disease
Stark (2020)[229]	Germany	O, L	61	1	0	Subacute ST-elevation myocardial infarction
Stollfuss (2021)[230]	Germany	O, L	—	18	12	Pulmonary arterial hypertension
Stone (2021)[231]	USA	O, CX	20.0 (1.6)	5	2	Healthy
Sun (2020)[232]	Denmark, Italy, Netherlands, Spain, UK	O, L	—	1062	—	Residing in European countries
Tabara (2020)[233]	Japan	O, CX	58.5 (11.7)	5916	4011	Without any physical impairment or dysfunction
Takahashi (2021)[234]	Japan	O, CX	50.0 (11.7)	86	48	No arrhythmias
Takami (2021)[235]	Japan	O, L	63 (16)	25	8	Long-term ECG monitoring
Tan (2021)[236]	UK	O, L	54.9 [52.3, 57.3]<br>71.0 [63.4, 76.8]	55	31	Healthy controls;<br>Normal tension glaucoma
Tayal (2020)[237]	India	O, CX	34.7 (15.0)	200	48	Outpatients
Teo (2021)[238]	Singapore	I, L	58.1 (10.3)	238	99	Hypertension
Toba (2021)[239]	Japan	O, CX	76.5 (8.6)	73	46	Hypertension outpatients
Tomitani (2021)[240]	Japan	O, CX	66.1 (10.8)	50	20	One or more cardiovascular risk factors
Treskes (2020)[241]	Netherlands	I, RCT	59.7 [52.9, 65.6]	200	44	ST-segment elevation myocardial infarction or non-ST-segment acute coronary syndrome
Trudel (2020)[242]	Canada	O, L	44.5 (8.6)	3547	—	White-collar workers with long working hours
Tsai (2022)[243]	Taiwan	O, L	46.2 (14.7)	59	36	Psychiatric clinic patients with panic disorder
Tseng (2020)[244]	USA	O, L	{18–42}	12	10	Students
Ushigome (2020)[245]	Japan	O, L	73.0 [67.5, 76.0]	41	17	Type 2 diabetes outpatients
Van Chien (2021)[246]	Vietnam	O, L	57.8 (12.5)	26	6	Outpatients with suspected cardiac arrythmias
Varas-Diaz (2020)[247]	USA	O, CX	64.8 (5.9)	45	28	Healthy; Persons with chronic stroke
Varga (2020)[248]	Romania	O, L	60 {52–64}	93	21	Patients with obstructive sleep apnea
Vischer (2022)[249]	Switzerland	O, L	—	50	24	Arterial hypertension, antihypertensive treatment
Vlahoyiannis (2021)[250]	Cyprus	I, RCT	23.9 (3.3)	10	0	Recreationally trained males
Vodička (2021)[251]	Slovenia	O, L	49.8 (16.4)	400	299	History of (suspected) cardiac rhythm disturbance
Vorwerg (2021)[252]	Germany	O, CX	73.9 (7.2)	43	21	Cardiac rehabilitation centre outpatients
Vybornova (2021)[253]	Switzerland	O, CX	38 (13)	86	43	Hypertensive outpatients
Wan (2021)[254]	China	O, CX	—	8	—	Heart-healthy
Wang (2022)[255]	USA	O, L	—	487	—	—

First author (year)	Country	Study design	Age, years	Sample size, N	Females, n	Population characteristics
Wattanapanyawech (2020)[256]	Thailand	I, RCT	20.6 (1.9)	30	21	Healthy
Weng (2021)[257]	USA	O, L	57.0 (10.6)	91	24	Recovering acute myocardial infarction
Weng (2021) [258]	Canada	O, L	62.6 (11.6)	94	39	New-onset non-valvular AF; Symptomatic AF
Wilson-Anumudu (2022)[259]	USA	I, L	44.0 (9.3)	151	85	Self-reported hypertension
Winter (2020)[260]	Canada	O, R	84 (5.3)	62	29	Aortic stenosis, TAVR
Wong (2020)[261]	Hong Kong	O, L	33.8 (11.0)	23	13	Healthy, various sporting backgrounds
Yamagami (2021)[262]	Japan	O, L	50.9 (20)	23	16	COVID-19
Yamakoshi (2021)[263]	Japan	O, CX	{10–73}	11	6	Healthy; Hypertension grade I
Yatabe (2021)[264]	Japan	I, RCT	53 (9)	97	58	Uncomplicated hypertension
Yen (2022)[265]	Taiwan	I, RCT	27.7 (6.6)	60	34	Healthy
Zhang (2020)[266]	USA	O, CX	39 (17)	11	1	Healthy
Zhang (2021)[267]	China	O, CX	78 (10)	291	122	Arrhythmias; Normal sinus rhythm
Zhang (2022)[268]	China	O, L	68.3 (4.7)	3724	1993	Hypertension
Zhang (2021) [269]	China	O, L	68.6 (4.8)	7394	3956	Hypertension
Zhang (2020)[270]	China	O, CX	51.7 (13.4)	43	21	Hospital staff; Hypertensive patients
Zhang (2021)[271]	China	O, CX	{21–35}	8	2	—
Zhu (2022)[272]	USA	O, CX	58.6 (18.6)	41	6	Diagnosed AF

Data are presented as mean (SD), mean {range}, median [IQR] or median* {range}, unless otherwise stated. IQR: interquartile range; SD: standard deviation.
If articles do not report the data, the symbol "—" is used.
Abbreviations: ABPM, ambulatory blood pressure monitoring; AF, atrial fibrillation; BMI, body mass index; COPD, chronic obstructive pulmonary disease; COVID-19, coronavirus disease 2019; CX, cross-sectional; ECG, electrocardiogram/electrocardiograph(y); HBPM, home blood pressure monitoring; I, interventional; L, longitudinal; O, observational; PCR, polymerase chain reaction; R, retrospective; RCT, randomized controlled trial; RPM, remote patient monitoring; SARS-CoV-2, severe acute respiratory syndrome coronavirus 2; SAVR, surgical aortic valve replacement; TAVR, transcatheter aortic valve replacement; UK, United Kingdom; USA, United States of America.

Appendix 4. Characteristics of the remote monitoring technologies (RMTs) measuring cardiovascular variables in community-dwelling adults described in the 272 included articles.

First author (year)	RMT (company, city, province/state/country, hardware version), Software/mobile application (version)	Measurement technology, Processing/analysis algorithm (version)	Cardiovascular physiological variable(s) measured	Wearing location (sensor position)
Agyün (2021)[1]	Polar S810i pulse watch + Polar chest band transmitter (Polar Electro, Finland)	—	HR	Chest
Ahn (2021)[2]	SM-R850 smartwatch (Samsung Electronics, Suwon, SK), Samsung Health Monitor app	LED PPG	SBP, DBP	Less affected side wrist
Akbar (2021)[3]	Garmin Vívosmart 3, Garmin Connect app	Optical heart rate sensor	HR, HRV	Wrist
Al Rajeh (2020)[4]	Nonin 3150 pulse oximeter	—	HR, SpO2	Wrist
	Nonin G92 pulse oximeter	—	HR, SpO2	Finger
Al-Kaisey (2020)[5]	Apple Watch Series 3, Health app	PPG	HR<br>Cardiac rhythm (AF)	Above the wrist
	Fitbit Charge HR, Fitbit app	PPG	HR<br>Cardiac rhythm (AF)	Above the wrist
Al-Naami (2021)[6]	Prototype: smart wearable monitoring device (SWMD)	LED PPG	PR, SpO2	Wrist
Alexandrou (2020)[7]	Mobil-O-Graph PWA (I.E.M., Germany)	Oscillometry, ARCSolver algorithm	HR, SBP, DBP	—
Aljuaid (2020)[8]	ECG Check device (Cardiac Designs Inc., San Francisco, CA), ECG Check app	1-lead ECG	ECG, Cardiac rhythm (SR, AF detection)	1 finger on each metal pad (2 total)
Amelard (2021)[9]	Polar H7 (Polar Electro Oy, Kempele, Finland)	—	HR	Chest
	Hexoskin shirt (Carré Technologies, Montréal, Canada)	Textile ECG	HR	Chest
Angelucci (2021)[10]	SAT-300 pulse oximeter (Contec Medical Systems Co., Ltd., Qinhuangdao, China)	—	HR, SpO2	Finger
Antali (2021)[11]	Berry Pulse Oximeter (Shanghai Berry Electronic Tech Co., Ltd., Shanghai, China), unspecified mobile app	PPG, study-developed SCN4ALL algorithm	PR, SpO2	Left index finger
	M3 Intellisense (Omron Corporation, Kyoto, Japan)	Oscillometry	SBP, DBP	Arm
Atkins (2020)[12]	Diasys 3 Plus (Novacor, Rueil-Malmaison, France)	Oscillometry, Novacor algorithms	PR, SBP, DBP	Upper arm
Atzmon (2020) [13]	Biobeat PPG-based wristwatch (Biobeat Technologies Ltd., Peta Tikva, Israel), study-developed smartphone app	Reflective PPG	HR, SBP, DBP, SpO2	Wrist
Avram (2021)[14]	Samsung Galaxy Watch Active 2 (Samsung, Seoul, SK), Eureka Platform mobile Application Program Interface	PPG + ECG	HR, Cardiac rhythm (SR, AF detection)	—
Baek (2021)[15]	Fitbit Charge 2	PPG	HR	Two-finger width above the ulnar styloid on the right wrist
	Polar H7 chest strap	ECG	HR	Chest
Bartlett (2021)[16]	KardiaMobile™ (AliveCor, Inc., Mountain View, CA)	1-lead ECG	ECG, Cardiac rhythm (SR, AF detection)	—
Batalik (2021)[17]	Polar M430 monitor (Kempele, Finland), PolarFlow application on the web	—	HR	Wrist
Batta (2022)[18]	Mobil-O-Graph NG device (I.E.M. GmbH, Germany)	Oscillometry, ARCSolver algorithm	HR, SBP, DBP, PWV	Left arm
Beers (2021)[19]	KardiaMobile 1L (AliveCor, Mountain View, CA), AliveCor smartphone app	1-lead ECG	HR, ECG (QTI measurement)	Handheld
Beime (2021)[20]	Microlife BP B3 AFIB/ERP monitor	Oscillometry	PR, SBP, DBP	Upper arm
Benedetti (2021)[21]	Fitbit ChargeHR™	PurePulse® LED PPG	HR	Wrist
Berryhill (2020)[22]	WHOOP Strap 2.0 (WHOOP, Inc., Boston, MA)	Reflectance PPG, WHOOP algorithms	HR, HRV	Nondominant arm
Blok (2021)[23]	Corsano CardioWatch/Bracelet 287 (Corsano Health B.V. Bussum, Netherlands), dedicated software on Android smartphone	PPG, study-developed algorithm	HR	Wrist

First author (year)	RMT (company, city, province/state/country, hardware version), Software/mobile application (version)	Measurement technology, Processing/analysis algorithm (version)	Cardiovascular physiological variable(s) measured	Wearing location (sensor position)
Böhm (2020)[24]	Spacelabs 90207 monitor (Spacelabs Healthcare, Snoqualmie, WA)	Oscillometry	HR, SBP, DBP	—
Bolin (2022)[25]	Heart Tracker (Biocom Technologies, USA)	PPG, Biocom Technologies software	HRV (measured by SDNN)	Ear
Bourassa (2020)[26]	Polar RS800CX (Polar Electro, Finland)	—	HR	Chest
Browne (2021)[27]	Samsung Galaxy S9 smartphone (Samsung, Seoul, SK): Maxim Integrated biosensors MAX86916 (Maxim Integrated, San Jose, CA), Samsung Health App	PPG	SpO2	Left forefinger placed over device
Browne (2021)[28]	ZUK Z2 X (ZUK; Lenovo), MAX86100A Android smartphone app (Maxim Integrated)	PPG, MaximFast algorithm	HR, SpO2	Index finger over sensor
Budig (2021)[29]	Polar H10 chest strap (Polar Electro Oy, Kempele, Finland, firmware 3.0.56, 2018), Polar Beat (version 3.4.7, 2020)	—	HR	Chest
	Polar Ignite (Polar Electro Oy, Kempele, Finland, firmware 2.0.25, 2019)	—	HR	Alternately on the left and right forearm behind the processus styloideus ulnae
	Garmin Forerunner 945 (Garmin, Ltd., Olathe, KS, firmware 5.50, 2019), Garmin Connect (version 4.37.2.0)	—	HR	
Burkhoff (2020)[30]	LifeVest™ Wearable Cardioverter Defibrillator (WCD) (ZOLL, Pittsburgh, PA)	ECG, automated WCD monitor algorithm	HR, ECG	Chest/body
Cao (2022)[31]	Oura Ring, Oura mobile app	PPG, Oura algorithm	HR, HRV	Nondominant hand finger
Capdevila (2021)[32]	Polar Band H7 (Polar Electro, Finland), FitLab® App iOS app	—	HR, HRV	Chest
Carnagarin (2022)[33]	Spacelabs 90207 or 90217 monitor (Spacelabs Medical, Inc., Redmond, WA)	Oscillometry	SBP, DBP	—
Castillo-Escario (2021)[34]	EMO-80 pulse oximeter (EMAY, Ltd., Hong Kong, China), EMAY Pulse Oximeter app	—, study-developed algorithms	PR, SpO2	Finger
Chalmers (2021)[35]	Fitbit Versa 2	PPG	HR	Non-dominant hand
Chang (2020)[36]	Polar H10 Heart Rate Monitor (Polar Electro Oy, Finland)	—	HR	Chest
Chaudhury (2021)[37]	Samsung Gear S3 smartwatch (Samsung Electronics, Inc., Ridgefield Park, NJ), study-developed smartphone app	PPG	HR	Wrist
Chen (2020)[38]	Amazfit Health Band 1S (Huami Technology, Anhui, China), unspecified smartphone app	1-lead ECG + PPG, RealBeats Artificial Intelligence Biological Data Engine algorithms	ECG, Cardiac rhythm (AF detection)	Wrist
Chen (2020)[39]	Polar H7 heart rate monitor (Polar Electro), Pulse Express Pro smartphone app	—, Kubios HRV Premium analysis software (version 3.2)	HR, HRV (measured by RMSSD, SDNN, pNN50, nVLF, nLF, nHF)	Chest
Chesebro (2020)[40]	Spacelabs 90207 monitor (Spacelabs, Redmond, WA)	Oscillometry	SBP, DBP	—
Chiang (2021)[41]	Omron Evolv wireless BP monitor, Omron Connect mobile app	—	SBP, DBP	—
	Samsung Galaxy Watch activity tracker, Samsung Health mobile app	—	HR	—
Cho (2022)[42]	KardiaMobile (AliveCor, Mountain View, CA)	1-lead ECG, KardiaMobile algorithm	ECG, Cardiac rhythm (AF detection)	—
Chorin (2021)[43]	CardiacSense	PPG, CardiacSense algorithm	HR, Cardiac rhythm (arrythmia detection)	Wrist
Chow (2020)[44]	Garmin Vívosmart HR+ (Garmin International Inc.)	3 LED PPG	HR	Wrist
	Xiaomi Mi Band 2 (Xiaomi Corporation), Cardio Training (Angelfmarcos) Android app	2 LED PPG	HR	Wrist

First author (year)	RMT (company, city, province/state/country, hardware version), Software/mobile application (version)	Measurement technology, Processing/analysis algorithm (version)	Cardiovascular physiological variable(s) measured	Wearing location (sensor position)
Christen (2020)[45]	QardioArm Smart BP Monitor (Qardio, San Francisco, CA), study-developed phone app	—	HR, SBP	Upper arm
	Apple SmartWatch (Apple)	—	HR	—
Climstein (2020)[46]	Polar Vantage M (Polar Electro, Kempele, Finland)	PPG	HR	Right wrist
Cooper (2020)[47]	Onyx II pulse oximeter (Nonin Medical, Plymouth, MN)	—	SpO2	—
Cos (2021)[48]	Fitbit Inspire HR (Fitbit, Inc.), study-developed data collection software	—	HR	—
Costa (2021)[49]	Spacelabs 90207 monitor (Spacelabs, Inc., Redmond, WA)	—	PR, SBP, DBP	—
Cuspidi (2020)[50]	Spacelabs 90207 monitor	—	HR, SBP, DBP	Arm
Dagel (2020)[51]	Mobil-O-Graph Arteriograph (I.E.M. GmbH, Stolberg, Germany)	Oscillometry, ARCSolver algorithm	PR, SBP, DBP, PWV	Upper left arm
Davidson (2020)[52]	Polar V800 watch (Polar Electro Oy, Kempele, Finland)	—	HR	Wrist
de la Casa Pérez (2022)[53]	Firstbeat Bodyguard 2 (Firstbeat Technologies, Ltd., Jyväskylä, Finland)	ECG, Kubios HRV standard software (version 3.5)	HR	—
	Xiaomi MiBand 4 (Xiaomi Corp., Beijing, China), Mi Fit mobile app	Photoelectric PPG	HR	Non-dominant wrist
de la Sierra (2021)[54]	Spacelabs 90207 monitor (Spacelabs, Snoqualmie, WA)	Oscillometry	SBP, DBP	—
Degott (2021)[55]	Samsung Galaxy S7 (Samsung GEC, Seoul, Korea), OptiBP mobile app	PPG, Biospectral SA algorithms	SBP, DBP	Fingertip on smartphone camera
Dehghani Zahedani (2021)[56]	MiBand 3 (Xiaomi Corp., Beijing, China), Sugar AI app	—	HR	—
Dinesen (2021)[57]	iHealth Neo blood pressure device	—	PR, SBP, DBP	—
	KardiaMobile ECG monitor (AliveCor)	ECG	ECG, Cardiac rhythm (AF detection)	—
Dörr (2021)[58]	iPhone 4S (Apple Inc., Cupertino, CA)	PPG, Preventicus algorithm (version 01/2017)	SBP	Index finger on smartphone camera
Dunietz (2020)[59]	Watch-PAT 200 (Itamar Medical, Caesarea, Israel)	—	SpO2	Finger (with wrist attachment)
Edwards (2020)[60]	Nonin 3230 pulse oximeter (Nonin Medical Inc.), patientMpower mobile/tablet app	—	SpO2	—
Elzinga (2021)[61]	Steel HR smartwatch (Withings), Withings Health Mate phone app	PPG	HR	Wrist
Fanget (2022)[62]	Dona Care connected watch (Life Plus, Versailles, France)	—	HR	—
Fouassier (2020)[63]	Cardioskin™ smart T-shirt (BioSerenity, Paris, France)	12-lead ECG (used by study) but CE-mark registered 15-lead ECG	HR, ECG (PR, QRS, QTc intervals)	Chest/body
Fu (2022)[64]	Triple-Physiology Sensor (TPS) (Thought Technology Ltd.©, Montréal, Quebec)	—	HR, HRV	Dominant hand finger
Gambassi (2020)[65]	Polar H7 electrode belt (Polar Electro Oy, Kempele, Finland), Elite HRV smartphone app (release 4.0.2, 2018)	ECG, Kubios HRV Standard software (release 3.1.0.1, 2018)	HRV (measured by mean NN interval, RMSSD, SDNN, pNN50)	At the xiphoid level just below the chest muscles
Garcia-Moreno (2022)[66]	Empatica E4 wristband sensor (Empatica Inc., Milan, Italy), study-developed gateway	—, study-developed algorithm	HR	Dominant hand wrist
Gazit (2021)[67]	A&D UA-651BLE BP, Hello Heart smartphone app	—	SBP and DBP	—
	Zewa UAM-900T, Hello Heart smartphone app	—	SBP, DBP	—
	Zewa UAM-910BT, Hello Heart smartphone app	—	SBP, DBP	—
Georgianos (2021)[68]	Mobil-O-Graph monitor (I.E.M., Stolberg, Germany)	Oscillometry	HR, SBP, DBP	Non-dominant arm

First author (year)	RMT (company, city, province/state/country, hardware version), Software/mobile application (version)	Measurement technology, Processing/analysis algorithm (version)	Cardiovascular physiological variable(s) measured	Wearing location (sensor position)
Gielen (2021)[69]	Biostrap wrist-worn device (Biostrap USA LLC, Duarte, CA), Biostrap app	PPG	HR, SpO2	Wrist
Girerd (2022)[70]	Withings BP monitor (Withings, Issy-les-Moulineaux, France)	—	HR, SBP, DBP	—
Giudicessi (2021)[71]	KardiaMobile 6L (AliveCor)	6-lead ECG, AliveCor algorithm	HR, ECG, Cardiac rhythm (QTc detection)	Handheld: both hands and left leg
Gkikopoulos (2022)[72]	Fitbit Alta HR (Fitbit, Inc., San Francisco, CA)	—	HR	—
Gladstone (2021)[73]	WatchBP-Home A (Microlife Corp)	Oscillometry	HR, SBP, DBP, Cardiac rhythm (AF detection)	—
Gognieva (2022)[74]	CardioQVARK® iPhone case, API service CardioQVARK®	1-lead ECG	HR, HRV, ECG, Cardiac rhythm (AF detection)	Handheld: index fingers on electrode surface
Golbus (2021)[75]	Apple Watch Series 3 or 4 (Apple, Cupertino, CA), MyDataHelps smartphone app	—	HR	Wrist
	Omron Evolv BPM (Omron Healthcare, Lake Forest, IL), MyDataHelps smartphone app	—	SBP, DBP	Bicep
Goldstein (2021)[76]	BB-613WP (Biobeat Technologies, Ltd., Petah-Tikva, Israel), dedicated gateway	Reflective PPG	PR, SBP, DBP, SpO2	Wrist
Gordon (2021)[77]	Samsung S9 or Note 9, MyBPLab app	PPG	HR, SBP, DBP	Handheld
Gordon (2020) [78]	Masimo MightSat pulse oximeter, MyChart Care Companion mobile app	PPG	SpO2	—
	Sensogram Sensoscan, MyChart Care Companion mobile app	PPG	SpO2	—
Goudman (2021) [79]	Polar H10 chest strap with Polar V800 heart rate monitor watch, Polar Flow app	ECG	HR, HRV (measured by RMSSD, SDNN, nLF, nHF, aHF)	Chest
Gräfitsch (2020)[80]	Santiago Telemonitoring Kit pulse oximeter (Health In Sight Solutions, GmBH, Munich, Germany)	—	PR, SpO2	—
Gresham (2021)[81]	Fitbit Charge HR 2	—	HR	—
Hahnen (2020)[82]	BodiMetrics Performance Monitor (BodiMetrics, Manhattan Beach)	ECG<br>ECG + PPG<br>PPG	HR<br>SBP<br>SpO2	Right index finger on PPG sensor, both hands on electrodes
	Everlast TR10 smartwatch	Electrodes + PPG sensor	HR, SBP	Wrist
Haveman (2022)[83]	Fitbit Charge 3 (Fitbit Inc.)	PPG	HR	Wrist
Hermans (2021)[84]	KardiaMobile® (AliveCor Inc., Mountain View, CA)	1-lead ECG	ECG, Cardiac rhythm (AF detection)	Index and middle fingers of each hand
Hillmann (2021)[85]	LifeVest WCD (ZOLL, Pittsburgh, PA)	Electrode belt	HR, HRV (SDNN)	Chest/body
Hochstadt (2020)[86]	CardiacSense wristwatch	PPG, Natan Lubman AF detection algorithm	HR, Cardiac rhythm (AF detection)	Wrist
Hofstede (2022)[87]	Connected HealthKit Pulsewave BP monitor (Cloud DX)	Oscillometry	HR, SBP, DBP	Non-dominant arm wrist
Hojjatinia (2021)[88]	AutoSense chest band	2-lead ECG	HRV	Chest
Holgado (2021)[89]	Polar H7 sensor band (Polar Electro, Finland) with Polar V800 monitor	—	HR	Chest
Holmes (2020)[90]	iPhone 6 (Apple Inc., Foxconn, Pegatron, Taipei, Taiwan), HRV4Training app	PPG, HRV4Training algorithm	HR, HRV (measured by lnRMSSD)	Right index fingertip over smartphone camera
Holmner (2020)[91]	WristOx model 3250 (Nonin Medical Inc., Plymouth, MN), mobile tablet gateway	—	HR, SpO2	Finger clip with wrist-worn display
Holyoke (2021)[92]	Contec CMS50EW (Contec Medical Systems Co Ltd), MediBeat smartphone app	PPG, HeartBeat algorithm	PR, SpO2	Handheld: index finger
Huang (2021)[93]	AViTA BPM65ZB sphygmomanometer	—	PR, SBP, DBP	Upper arm

First author (year)	RMT (company, city, province/state/country, hardware version), Software/mobile application (version)	Measurement technology, Processing/analysis algorithm (version)	Cardiovascular physiological variable(s) measured	Wearing location (sensor position)
Huang (2020)[94]	A&D UA-767PBT-Ci, Koneksa Health app	Oscillometry	SBP, DBP	Upper arm
	Preventice BodyGuardian, Koneksa Health app	1-lead ECG	HR	Chest
Huppertz (2020)[95]	Microlife WatchBPO3 AFIB (Microlife, Heerbrugg, Switzerland)	Oscillometry, Microlife algorithm	PR, SBP, DBP, Cardiac rhythm (AF detection)	Upper arm
Hutchings (2021)[96]	iHealth Air pulse oximeter PO3M (iHealth Labs, Inc.)	—	PR, SpO2	Finger
Huynh (2021)[97]	Apple Watch Series 0	PPG	HR	—
Ingvaldsen (2021)[98]	Cerebri sensor (Nordic Brain Tech AS), Brain Twin smartphone app	—	HR	Index finger
Inui (2020)[99]	Apple Watch Series 3 (Apple Inc.)	PPG	PR, Cardiac rhythm (AF detection)	Forearm wrist
	Fitbit Charge HR (Fitbit Inc.)	PPG	PR, Cardiac rhythm (AF detection)	Forearm wrist
Ioachimescu (2020)[100]	WatchPat 200 device (Itamar Medical, Caesarea, Israel)	PAT, WatchPat algorithm	SpO2	Finger with wrist attachment
Ionov (2021)[101]	BPLab (Petr Telegin Ltd., Russian Federation)	Oscillometry	SBP, DBP	—
Iqbal (2021)[102]	SensiumVitals™ ECG patch (The Surgical Company, Oxford, UK)	1-lead ECG, SensiumVitals™ algorithms	HR	Chest
Jachymek (2021)[103]	Fitbit Charge 4 (Fitbit), Fitbit mobile app	PPG	HR	Wrist
	Xiaomi Mi Band 5 (Xiaomi), Google FIT app	PPG	HR	Wrist
Jen (2020)[104]	WatchPAT200 (Itamar Medical Ltd., Caesarea, Israel)	PAT, WatchPAT algorithm	HR, SpO2	Finger
Jones (2021)[105]	Garmin Vívosmart® HR+	—	HR	Wrist
Jortveit (2022)[106]	ECG247 Smart Heart Sensor (AppSens, Lillesand, Norway), ECG247 mobile app	1-lead ECG	ECG, Cardiac rhythm (arrhythmia detection)	Chest
Kaile (2021)[107]	SmartPhone Oxygenation Tool (SPOT) adapted to an Android-based smartphone Samsung Galaxy 7.0, study-developed app	Near-infrared spectroscopy (NIRS)	Tissue oxygenation: changes in oxygenation (HbO and HbR)	Handheld
Kalanadhabhatta (2021)[108]	Fitbit Charge 3 (Fitbit Inc.), Fitbit app and study-designed smartphone app	—	HR	Nondominant hand
Kanady (2020)[109]	Basis B1 (Basis Science, Inc., San Francisco, CA)	PPG, Basis B1 algorithm	HR	Nondominant wrist
Kańtoch (2021)[110]	Fitbit Versa, Fitbit cloud app	—	HR	Wrist
Kario (2021)[111]	UA-651BLE (A&D Co.), HERB Mobile app	—	SBP, DBP	—
Kario (2021)[112]	TM-2441 device (A&D Co.), HERB Mobile app	Oscillometry	Ambulatory BP	—
	UA-651BLE (A&D Co.), HERB Mobile app	Oscillometry	Morning home BP	—
Kario (2020)[113]	HeartGuide device (Omron Healthcare Co., Ltd.)	Oscillometry	PR, SBP, DBP	Nondominant arm wrist
	TM-2441 (A&D Company)	Oscillometry	PR, SBP, DBP	Upper arm
Khattak (2021)[114]	Samsung Galaxy phone, Samsung Health/S Health smartphone app	PPG	SpO2	Right index finger held to phone camera
Khushhal (2020)[115]	Apple Watch Series 0 or 2 (Apple Inc., California, USA, WatchOS 2.0.1), Workout app	PPG	HR	Left wrist
Kilic (2020)[116]	Mobil-O-Graph Arteriograph (I.E.M. GmbH, Stolberg, Germany)	Oscillometry, Mobil-O-Graph algorithm	HR, brachial BP (SBP, DBP, MAP), PWV	—
Kim (2020)[117]	HEM-9200T (Omron Healthcare, Co Ltd, Kyoto, Japan), study-developed Android app	—	SBP, DBP	—
Kim (2021)[118]	Fitbit Charge HR (Fitbit)	PPG	HR	Wrist
Kim (2020)[119]	Xiaomi Mi Band Pulse 1S	PPG	HR	Wrist

First author (year)	RMT (company, city, province/state/country, hardware version), Software/mobile application (version)	Measurement technology, Processing/analysis algorithm (version)	Cardiovascular physiological variable(s) measured	Wearing location (sensor position)
Kim (2020)[120]	Polar RS800CX (Polar® Electro Oy, Kempele, Finland), Pro-Trainer Polar 5 software (version 5.40.171)	2-lead ECG, Kubios HRV analysis software (version 2.1, 2012)	HR, HRV (measured by RMSSD, pNN50, HF, LF, LF/HF ratio)	Chest
Kim (2021)[121]	Fitbit Charge 2 (Fitbit, San Francisco, CA, firmware version 22.58.0), Fitbit mobile app	PPG	HR	Right wrist
Kim (2020)[122]	Apple Watch 2 Nike+ (Apple, Inc.)	—	HR	—
Kim (2020)[123]	Neofit (Partron Co), Second Wind mobile app	—	HR	Wrist
Kinnunen (2020)[124]	Oura Ring Gen2 (OURA, Oulu, Finland), unspecified smartphone app	2 LED PPG, Oura Algorithm	HR, HRV (measured by RMSSD)	—
Ko (2022)[125]	ASUS VivoWatchBP (ASUSTeK Computer Inc., Taipei, Taiwan)	PPG, study-developed algorithm	HR	Wrists (at least 2 cm away from ulna bone)
Koh (2021)[126]	KardiaMobile (AliveCor®, Mountain View, CA), AliveCor® KardiaMobile smartphone app	1-lead ECG	ECG, Cardiac rhythm (AF detection)	Two fingers from each hand on electrodes
Kokubo (2020)[127]	Beat-by-Beat BP monitoring device	Oscillometry + tonometry	PR, SBP, DBP	Left wrist
Kolkenbeck-Ruh (2022)[128]	Mobil-O-Graph (I.E.M. GmbH, Aachen, Germany), I.E.M. HMS Client-Server (version 5.2)	Oscillometry, ARCSolver algorithm	HR, brachial BP (SBP, DBP), PWV	Right upper arm
	Omron MIT5 (Omron Healthcare, Kyoto Japan)	—	SBP, DBP, MAP	Right upper arm
Krhut (2021)[129]	CardioMem CM 3000 device (Getemed, Teltow, Germany)	2- or 3-lead ECG	HR, ECG, Cardiac rhythm (ectopic beats)	—
	Ultralite™ 90217A monitor (Spacelabs Healthcare, Hertford, UK)	—	SBP, DBP	—
Kuula (2021)[130]	Firstbeat Bodyguard 2	2 chest electrodes, Firstbeat algorithm	HRV	Chest
Kwon (2021)[131]	mobiCARE-MC100 (Seers Technology, Seongnam-si, Republic of Korea), unspecified smartphone app	1-lead ECG	HR, ECG, Cardiac rhythm (QRS complex, ectopic beats)	Chest (precordium)
Kwon (2021)[132]	MotionSense HRV (MD2K), mCerbrum app	PPG	HR, HRV	Non-dominant wrist
Lai (2021)[133]	Samsung Gear Sport smartwatch, Samsung Android mobile app	—	HR, HRV (measured by RMSSD)	Wrist
Lambert (2021)[134]	KardiaMobile (AliveCor, Mountain View, CA), KardiaPro platform web portal	1-lead ECG	HR, ECG, Cardiac rhythm (AF detection)	Handheld
Lan (2020)[135]	Study-developed prototype device, study-developed Android mobile app	PPG	PR	Wrist (adjacent styloid process of radius bone)
Larsen (2022)[136]	Garmin Vívosmart 4 (Garmin Ltd, Schaffhausen, Switzerland), Garmin Connect mobile app	Optical PPG	HR, HRV (measured by RMSSD)	Wrist
Lauder (2020)[137]	Lifecard CF (Spacelabs Healthcare)	ECG	HR, Cardiac rhythm (SR, AF detection)	—
	Mobil-O-Graph New Generation 24-h ABPM classic (I.E.M. GmBH, Stolberg, Germany)	Oscillometry	PR, SBP, DBP, Cardiac rhythm (SR, AF detection)	Left or right brachial artery
Layton (2021)[138]	Peloton Heart Rate Band (Peloton, New York, NY), Peloton app	—	HR	—
Li (2021)[139]	Recovery Plus heart rate band (Recovery Plus Inc.), R Plus Health app	—	HR	Chest
Liu (2021)[140]	KardiaMobile® EKG 6L (AliveCor, Mountain View, CA), KardioMobile® EKG monitoring smartphone app	6-lead ECG (3 electrodes)	ECG (PR, QRS, QTc intervals)	Handheld: inside of left ankle with both thumbs on sensors
Liu (2020)[141]	Fitbit Alta (Fitbit Inc.)	PPG	HR	Nondominant hand
Liu (2022)[142]	SpaceLabs 90217 monitor (SpaceLabs Medical Inc., Redmond, WA)	Oscillometry	SBP, DBP	Arm
Liu (2022)[143]	Huawei Watch GT 2 Pro ECG edition (Huawei Device Co., Shenzhen, China), Huawei Watch GT 2 Pro app (2020–2021)	—	HR, SpO_2, ECG	—

First author (year)	RMT (company, city, province/state/country, hardware version), Software/mobile application (version)	Measurement technology, Processing/analysis algorithm (version)	Cardiovascular physiological variable(s) measured	Wearing location (sensor position)
Lo (2020)[144]	ANSWatch Model TS-0411 (Taiwan Scientific Corp., New Taipei City, Taiwan)	Piezoelectric sensors	HR, HRV (measured by nHF, nLF, LF/HF ratio)	Left wrist radial artery
Low (2021)[145]	Fitbit Charge 2, AWARE Android mobile app	—	HR	—
Lumikari (2020)[146]	Beat2Phone device (VitalSignum, Helsinki, Finland), VitalSignum mobile phone app	1-lead ECG	HR, ECG, Cardiac rhythm (AF detection)	Waist
Luštrek (2021)[147]	HeartMan wristband (study-developed prototype), custom HeartMan mobile app	PPG	HR (b2b intervals)	Wrist
Mach (2020)[148]	Vívosmart 3 (Garmin, software version 2.9–5.10), Garmin Connect app (version 3.22.0.1–4.20)	PPG	HR	Nondominant hand wrist
Magnusson (2020)[149]	Coala Heart Monitor (Coala Life AB, Stockholm, Sweden), unspecified smartphone app	ECG	Cardiac rhythm (SR, AF detection)	Held with thumb and forefinger, placed on chest
Maille (2021)[150]	Withings Move ECG™ smartwatch, Cardiologs® smartphone app	1-lead ECG, AI-QTc Cardiologs® algorithm	ECG (QTc interval)	Wrist
Marcelli (2021)[151]	Venous Congestion Meter (VenCoM) prototype device, Graphical User Interface on laptop	Venous occlusive plethysmography (VOP)	BP (central venous pressure)	Upper arm and forearm
Marcus (2022)[152]	KardiaMobile (AliveCor, Mountain View, CA)	ECG	ECG, Cardiac rhythm (AF detection)	—
Martini (2022)[153]	Movesense HR+ heart rate sensor (Movesense by/& Suunto, Vantaa, Finland), Heart Sentinel smartphone app	1-lead ECG, RITMIA™ algorithm	HR, ECG, Cardiac rhythm (AF detection)	Chest
Mason (2022)[154]	Oura Ring Generation 2 (Oura Helath, Oulu, Finland), Oura smartphone app	PPG	HR, HRV	Finger of participant's choosing
McManus (2021)[155]	Omron M3 monitor, HOME BP online	—	SBP, DPB	—
McNeil (2022)[156]	Polar A360® activity tracker (Polar Electro Oy, Kempele, Finland), Polar Flow® app	—	HR	Wrist
Mena (2020)[157]	Omron HEM-4030	Oscillometry	SBP, DBP	Nondominant upper arm
	Study prototype: TSL2561 luminosity sensor (Adafruit Industries) + infrared LED LTE-302 (Lite-On), study-developed app	PPG, combinatorial ANN model	SBP, DBP, MAP	Nondominant arm wrist
Meng (2020)[158]	Fitibit Charge 2 (Fitbit Inc., San Francisco, CA)	—	HR	—
Mhajna (2020)[159]	Invu wearable belt (Nuvo-Group, Ltd, Tel Aviv, Israel), Invu mobile app	ECG-like sensor, Invu algorithm	HR (maternal)	Abdomen/waist
Miller (2020)[160]	WHOOP strap (CB Rank, Greater Boston, New England, USA, Generation 2.0 hardware), WHOOP smartphone app	PPG, WHOOP strap Generation 3 algorithm	HR, HRV	Non-dominant wrist
Miranda Hurtado (2021)[161]	Bpro (HealthSTATS International, Singapore)	Tonometry	SBP, DBP	Right wrist
Mishra (2020)[162]	Polar H7, Amulet wrist device (data hub)	1-lead ECG	HR, HRV	Chest
	Zephyr HxM HR Monitor, Amulet wrist device (data hub)	—	HR, HRV	Chest
Mocny-Pachońska (2020)[163]	Garmin Vívoactive 3 GPS smartwatch	—	HR	—
Modi (2021)[164]	Kenek Edge (LionsGate Technologies, Vancouver, Canada) pulse oximeter probe attached to Apple iPhone 6S (Apple, Cupertino, USA), Kenek Edge mobile app	PPG	PR, SpO2	Finger
	Samsung S8 (Sansung Electronics, Seoul, SK), Samsung Health mobile app	PPG	PR, SpO2	Handheld
Moitinho-Silva (2021)[165]	Garmin® Vívosport fitness tracker, Garmin® Connect smartphone app	—	HR	Wrist

First author (year)	RMT (company, city, province/state/country, hardware version), Software/mobile application (version)	Measurement technology, Processing/analysis algorithm (version)	Cardiovascular physiological variable(s) measured	Wearing location (sensor position)
Montrivade (2020)[166]	Upright model TD-3128 (TaiDoc Corporation, Taiwan)	Oscillometry	SBP, DBP	Arm
Moon (2020)[167]	InBodyWATCH (InBody Co., Ltd., Seoul, SK), InBody Android smartphone app	PPG + ECG, InBody algorithm	HR, SBP, DBP	Left wrist
Morgado Areia (2021)[168]	VitalPatch® RTM (VitalConnect USA), study-developed Android tablet-based app	1-lead ECG	HR, HRV, ECG (QRS interval)	Chest: left midclavicular line over intercostal space
Moshe (2021)[169]	Oura Ring, Delphi iOS smartphone app	—	HRV	—
Motta (2021)[170]	BIC model YK-80A finger pulse oximeter, study-developed Android app	—	HR, SpO2	Finger
Mugabirwe (2021)[171]	Omron VR BP710N 3 series (Omron Healthcare Inc., Bannockburn, USA), PositiveLinks app	Oscillometry	SBP, DBP	Arm
Muggeridge (2021)[172]	Fitbit Charge 3, Fitbit app	PurePulse wrist HR technology	HR	Nondominant wrist: 2-finger widths above ulnar styloid process
	Polar OH1 (Polar Electro Oy), Polar Beat smartphone app	6 LED PPG	HR	Nondominant forearm
Murase (2022)[173]	HEM-7080IC (Omron Healthcare, Inc., Kyoto, Japan)	Oscillometry	SBP, DBP	—
	PULSOX-Me300 (Konica Minolta, Inc., Tokyo, Japan)	—	Oxygen desaturation	—
Nabasny (2022)[174]	Polar H10 Heart Rate Sensor (Polar Electro Oy, Kempele, Finland), Elite HRV smartphone app	—, Elite HRV algorithms	HRV (measured by RMSSD)	Chest
Nissen (2022)[175]	Fitbit Charge 4 (Fitbit, Inc., San Francisco, CA), Fitbit mobile app	—, Fitbit algorithm	HR	Arm
	Samsung Galaxy Watch Active2 (Samsung Group, Suwon-si, SK), Tizen OS Human Activity Monitor API	PPG (8 photodiodes)	HR, BP, ECG	Arm
Nolde (2021)[176]	Spacelabs BP monitor (Spacelabs, Snoqualmie, WA)	—	HR, SBP, DBP	Arm
	Mobil-O-Graph BP monitor (I.E.M. GmbH, Stolberg, Germany)	—	HR, SBP, DBP	Arm
	OSCAR-2 BP monitor (SunTech, Morrisville, North Carolina)	—	HR, SBP, DBP	Arm
Ocagli (2021)[177]	Garmin© Vívoactive® 3 smartwatch, Garmin Connect© app	—	HR	Wrist
O'Driscoll (2020)[178]	Fitbit Charge 2 (Fitbit Inc., San Francisco, CA), Fitbit mobile app	LED PPG: PurePulse technology	HR	A finger's width above non-dominant wrist
	Polar H7 chest strap with Polar m400 HR Monitor Watch (Polar Electro, Kempele, Finland), Polar Flow online app	—	HR	Chest
Oh (2022)[179]	HEM-9200T BP monitor (Omron), study-developed Android smartphone app	Oscillometry, Omron algorithm	SBP, DBP	Upper arm
Ohtsuka (2021)[180]	WatchPAT200 (Itamar Medical, Caesarea, Israel)	—	HR, SpO2	—
Olbers (2021)[181]	SpaceLabs 90217a device (Spacelabs, Snoqualmie, WA)	—	HR, SBP, DBP	—
Omboni (2020)[182]	BPLab device (BPLab GmbH, Schwalbach am Taunus, Hessen, Germany), THOLOMEUS telehealth platform	Oscillometry, THOLOMEUS algorithm	SBP, DBP, central arterial pressure, PWV	Non-dominant mid-upper arm
Ong (2021)[183]	Fitbit Ionic (Fitbit Inc, San Francisco, CA)	—	HR	Wrist
Panula (2020)[184]	Omron Intellisense M6	—	SBP, DBP	—
Park (2022)[185]	BioHarness-3™ ECG chest strap (Zephyr Technologies, Annapolis, MD)	1-lead ECG, Kubios HRV Premium Software (Version 2.0)	HR, HRV (SDNN, HF, LF), ECG	Chest: lower sternum
Pavic (2020)[186]	Everion® bracelet (Biovotion AG®, Zurich, Switzerland), Active Monitoring Android app	—	HR, HRV (RMSSD), SpO2	Upper arm

First author (year)	RMT (company, city, province/state/country, hardware version), Software/mobile application (version)	Measurement technology, Processing/analysis algorithm (version)	Cardiovascular physiological variable(s) measured	Wearing location (sensor position)
Persell (2020)[187]	7 Series Wireless Upper Arm BP Monitor Model BP761N (Omron), Omron Wellness mobile app (for Control group) or HTN Pro mobile app (beta version: Hypertension Personal Control Program (HPCP) coaching app for Intervention group)	—	SBP, DBP	Upper arm
	Global Model HEM-7320T (Omron Healthcare Co Ltd), Omron Wellness mobile app (for Control group) or HTN Pro mobile app (beta version: Hypertension Personal Control Program (HPCP) coaching app for Intervention group)	—	SBP, DBP	—
Pinheiro (2020)[188]	Oxistar™ wireless oximeter (Biologix Sistemas Ltd., Brazil), Overnight Digital Monitoring (ODM) smartphone app	PPG, ODM algorithm	PR, oxygen desaturation	Finger
Pipek (2021)[189]	Apple Watch Series 6 (Apple, CA)	—	HR, SpO2	Left wrist
Polverino (2022)[190]	Diamond Cuff BP – P80 (ForaCare, Inc., Moorpark, California, USA), mobile gateway device	—	HR, SBP, DBP	—
	OxyWatch pulse oximeter (ChoiceMMed, Beijing, China), mobile gateway device	—	HR, SpO2	—
Porumb (2020)[191]	Zephyr BioPatch™ HP (Medtronic)	—, QRS detection algorithm and study-developed CNN + RNN network model	Heartbeats, ECG	—
Praus (2021)[192]	KardiaMobile (AliveCor), Kardia and NowClinic smartphone apps	1-lead ECG (2 electrodes)	ECG, Cardiac rhythm (AF detection)	—
Prigent (2021)[193]	Polar H10 HR monitor (Polar®, Finland)	1-lead ECG	HR	Chest
Puhr-Westerheide (2020)[194]	Wellysis S-Patch Cardio solution, Android device gateway	1-lead ECG	HR, ECG (QRS interval, ms), Cardiac rhythm (ectopic beats)	Chest
Quer (2020)[195]	Fitbit Charge HR versions 1–3, Blaze, Alta HR, Ionic, Surge, or Versa (Fitbit, Inc., San Francisco, CA)	PPG, Fitbit algorithm	HR	Wrist
Rodrigues (2022)[196]	Fitbit Inspire HR (Fitbit Company, San Francisco, CA), Fitbit smartphone app	PPG, Fitbit algorithm	HR, HRV (measured by RMSSD, SDNN, pNN50)	Left wrist: two fingers above styloid process of ulnar bone
	Polar H10 heart rate monitor (Polar Electro Brasil Comércio, Distribuição, Importação e Exportação Ltd.)	—, Kubios HRV software	HR, HRV (measured by RMSSD, SDNN, pNN50)	Chest
Roe (2020)[197]	Huawei Watch2, study-developed app	PPG, bandpass filters	HR, HRV (measured by RMSSD)	Wrist
Rykov (2021)[198]	Fitbit Charge 2, Fitbit mobile app	—	HR	—
Rykov (2020)[199]	Fitbit Charge 2, Fitbit mobile app	—	HR	Wrist
Saarikko (2020)[200]	Garmin Vívosmart, mobile gateway device	PPG, Garmin algorithm	HR	Wrist
Saghir (2020)[201]	Apple Watch Series 4 (watchOS 5.2), Apple Health app (iOS 12.3.1)	1-lead ECG	HR, ECG (QRS, ST, QT, QTc intervals)	Left wrist
Sakulsupsiri (2021)[202]	Upright model TD-3128 (TaiDoc Technology Corporation, Taiwan)	Oscillometry	SBP, DBP	—
Salazar (2020)[203]	Spacelabs 90207 (Spacelabs, Snoqualmie, WA)	Oscillometry	SBP, DBP	Arm
Salvi (2021)[204]	PC-68B (Shenzhen Creative Industry Co Ltd), SMWT (study-developed) phone app	—	HR, SpO2	—
	WristOx 2 3150 (Nonin), SMWT (study-developed) phone app	—	HR, SpO2	—
Saner (2020)[205]	Everion® armband (Biovotion AG, Zürich, Switzerland), unspecified smartphone app	—	HR, HRV (measured by RMSSD)	Upper arm
Sardana (2021)[206]	Apple Watch Series 0	—	HR	—

First author (year)	RMT (company, city, province/state/country, hardware version), Software/mobile application (version)	Measurement technology, Processing/analysis algorithm (version)	Cardiovascular physiological variable(s) measured	Wearing location (sensor position)
	Wireless BP cuff (Nokia Withings)	—	SBP, DBP	Left arm
Sarganas (2020)[207]	Mobil-O-Graph PWA device (I.E.M., Stolberg, Germany)	Oscillometry, Mobil-O-Graph algorithm	SBP, DBP	Upper arm
Sasaki-Otomaru (2020)[208]	Pulsesense® PS-500B pulsimeter (Seiko Epson Co. Ltd., Nagano, Japan)	—	PR	Non-dominant arm wrist (generally left)
	UA-772 automated device (A&D Company, Ltd., Tokyo, Japan)	—	SBP, DBP	Upper arm
Sato (2021)[209]	Watch-PAT 200 (Itamar Medical, Caesarea, Israel)	PAT, Watch-PAT algorithm	HR, arterial oxygen saturation	Finger
Savvari (2020)[210]	Microlife BP A6 PC	Oscillometry, mean and SD of pulse intervals mapped to irregularity index	PR, SBP, DBP, Cardiac rhythm (AF detection)	Arm
Sayer (2022)[211]	LiveOne device (LiveMetric S.A., Luxemborg)	Pressure sensor, LiveOne algorithm	SBP, DBP	Wrist
Schubert (2020)[212]	Philips health watch DL8791 (Philips, Stamford, CT, USA)	—	HR	Wrist
Schwartz (2020)[213]	HEM-790IT [HEM-7080-ITZ2] or HEM-791IT [HEM-7222-ITZ] (Omron Healthcare Inc., Lake Forest, Illinois)	Oscillometry	SBP, DBP	Nondominant arm
	Spacelabs Model 90207 (Spacelabs Healthcare, Snoqualmie, WA)	—	SBP, DBP	Nondominant arm
Selder (2020)[214]	Kardia Band (AliveCor, Mountain View, CA) attached to Apple Watch (Apple Inc., Cupertino, CA), unspecified app on Apple iPad	1-lead ECG, AliveCor Kardia algorithm	Cardiac rhythm (AF detection)	Wrist
	Wavelet wristband (Wavelet Health, California, US), unspecified app on Apple iPad	PPG, Fibricheck algorithm	Cardiac rhythm (AF detection)	Wrist
Sequeira (2020)[215]	Apple Watch (Apple Inc, Cupertino, CA), Apple Health smartphone app	PPG, Apple algorithms	HR	Wrist above the distal radioulnar joint
	Fitbit Charge HR (Fitbit Inc, San Francisco, CA), Fitbit smartphone app	PPG, Fitbit algorithms	HR	Wrist above the distal radioulnar joint
	Garmin Vívosmart HR (Garmin, Ltd, Olathe, KS), Garmin smartphone app	PPG, Garmin algorithms	HR	Wrist above the distal radioulnar joint
	Polar A360 (Polar Electro Oy, Kempele, Finland), Polar Flow smartphone app	PPG, Polar algorithms	HR	Wrist above the distal radioulnar joint
Shan (2020)[216]	BP3L BP monitor (iHealth Lab Inc.), Corrie Health smartphone app	—	SBP, DBP	Arm
Sher (2022)[217]	Fitbit 2 (Fitbit, Inc., San Francisco, CA)	—	HR	—
Shu (2020)[218]	Algoband F8 (Desay Electronics, Huizhou, China)	PPG	PR	Wrist
Shufelt (2020)[219]	Fitbit Charge 2 (Fitbit Inc., San Francisco, CA)	—	HR	—
Shui (2021)[220]	Custom-designed wristband (Pyschorus, HuiXin, Beijing, China)	PPG	HR	Wrist
Shumate (2021)[221]	Polar Vantage M (Polar Electro, Kempele, Finland)	PPG	HR	Right hand
Sjöberg (2021)[222]	Fitbit Versa (Fitbit Inc)	PPG	HR	Nondominant wrist
Slade (2021)[223]	Garmin Vívofit	—	HR	Wrist
So (2021)[224]	Upmood band, Upmood Android mobile app	PPG	PR, HRV	Wrist
Sokas (2021)[225]	Fitbit Alta HR (Fitbit, San Francisco, CA)	PPG	HR	Wrist
	Fitbit Charge 2 (Fitbit, San Francisco, CA)	PPG	HR	Wrist
Sola (2021)[226]	Aktiia bracelet, Aktiia companion smartphone app	PPG, Aktiia pulse wave analysis algorithms	SBP, DBP	Right wrist
Song (2020)[227]	Globalcare GCE603 (Globalcare Medical Technology Co., Ltd, Zhongshan, China)	Oscillometry	SBP, DBP	Upper arm
Spaccarotella (2022)[228]	Apple Watch Series 6 (Apple Inc., Cupertino, CA)	4 LED clusters, 4 photodiodes	HR, SpO2	Wrist

First author (year)	RMT (company, city, province/state/country, hardware version), Software/mobile application (version)	Measurement technology, Processing/analysis algorithm (version)	Cardiovascular physiological variable(s) measured	Wearing location (sensor position)
Stark (2020)[229]	Apple Watch 5	1-lead ECG	ECG, Cardiac rhythm (AF detection)	Left arm wrist
Stollfuss (2021)[230]	Apple Watch Series 2, xbird GmbH mobile app	—	HR	Wrist
Stone (2021)[231]	Firstbeat Textile Strap (Firstbeat, Jyväskylä, Finland), Firstbeat smartphone app	ECG	HR, HRV	Chest
	iPhone 8 (Apple, CA), HRV4Training + EliteHRV + Camera HRV smartphone apps	LED PPG	HR, HRV	Handheld: fingertip
	Oura Ring Gen2 (OURA, Oulu, Finland), OURA smartphone app	Infrared PPG sensor	HR, HRV	Finger
	Polar H10 strap (Polar , Kempele, Finland), HRV4Training + EliteHRV smartphone apps	ECG	HR, HRV	Chest
	Shimmer3 mECG Unite (Shimmer, Dublin, Ireland), iMotions software	5-lead ECG	HR, HRV	Chest
Sun (2020)[232]	Fitbit Charge 2 and Fitbit Charge 3 devices, Fitbit Android app	—	HR	Nondominant hand
Tabara (2020)[233]	HEM-7080IC (Omron Healthcare, Kyoto, Japan)	Oscillometry	SBP, DBP	Upper arm
Takahashi (2021)[234]	HEM-7600T (Omron Healthcare Co., Ltd., Kyoto, Japan)	Oscillometry	HR, SBP, DBP	Upper arm
Takami (2021)[235]	Duranta ECG monitoring device (ImageONE Co., Ltd.), unspecified iPhone app	ECG (2 electrodes)	ECG, Cardiac rhythm (arrythmia detection)	Chest
Tan (2021)[236]	Model ABPM50 (Contec Medical Systems, Hebei, China)	—	PR, SBP, DBP, MAP	Nondominant arm
Tayal (2020)[237]	Remote monitoring wearable device (Electronics Corporation of India Limited (ECIL), Hyderabad, India)	PPG, ECIL algorithms	PR, SpO2	Left hand index finger
Teo (2021)[238]	FORA P20b BP Monitoring System (TaiDoc Technology), Phicomm C230 gateway device	—	SBP, DBP	—
Toba (2021)[239]	HEM-7080IC (Omron, Kyoto, Japan)	Oscillometry	SBP, DBP	Arm
Tomitani (2021)[240]	"HeartGuide": HEM-6410T (Omron Healthcare, Kyoto, Japan)	Oscillometry	HR, SBP, DBP	Nondominant arm wrist
Treskes (2020)[241]	Kardia (AliveCor Inc), Kardia smartphone app	1-lead ECG (2 electrodes)	ECG	Handheld: 2 fingers from each hand
	BP Monitor Connect (Withings), device-dedicated smartphone app	Oscillometry	SBP, DBP	Bare upper arm
Trudel (2020)[242]	Spacelabs 90207 monitor (Spacelabs Produits Médicaux Ltée, St-Laurent, Quebec)	—	SBP, DBP	Nondominant arm
Tsai (2022)[243]	Garmin Vívosmart 4 (Garmin Ltd, Schaffhausen, Switzerland)	—	HR	—
Tseng (2020)[244]	Polar H10 HR monitor, InhibiSense study-developed phone app	—	HRV	—
Ushigome (2020)[245]	HEM-7251G BP monitor (Omron Healthcare Co., Ltd, Kyoto, Japan), Medical LINK® server-connected mobile gateway	Oscillometry	SBP, DBP	Non-dominant arm (cuff positioned at heart level)
Van Chien (2021)[246]	Spyder ECG monitoring device (Dr. P. Wong, Singapore National Heart Centre), Doctor Spyder server-connected mobile gateway	1-lead ECG	ECG, Cardiac rhythm (AF detection)	Left chest
Varas-Diaz (2020)[247]	Polar RS800CX (Polar Electro Oy, Kempele, Finland), POLAR PRO trainer 5	2-lead ECG, Kubios analysis software (version 4.0, 2012)	HR, HRV (RMSSD, nHF)	Chest
Varga (2020)[248]	CardioPoint H100 Holter (BTL Industries Limited, UK)	3-lead ECG	PR, Cardiac rhythm (extrasystoles, AF detection, tachycardia, cardiac activity pauses)	—
Vischer (2022)[249]	iPhone 6S (Apple Inc., Cupertino, CA, iOS 15.1), RIVA Digital Blood Pressure app	PPG	SBP, DBP	Non-dominant arm index, middle, or ring finger
Vlahoyiannis (2021)[250]	Polar® H7 HR monitor (Polar Electro Oy®, Kempele, Finland)	—	HR	—

First author (year)	RMT (company, city, province/state/country, hardware version), Software/mobile application (version)	Measurement technology, Processing/analysis algorithm (version)	Cardiovascular physiological variable(s) measured	Wearing location (sensor position)
Vodička (2021)[251]	Savvy ECG sensor (Jozef Stefan Institute), MobECG smartphone app	ECG (2 electrodes)	ECG, Cardiac rhythm	Chest
Vorwerg (2021)[252]	Polar M600 smartwatch	PPG	HR	Wrist
Vybornova (2021)[253]	Aktiia Bracelet (Aktiia SA), Aktiia smartphone app	PPG, Aktiia pulse wave analysis algorithm	SBP, DBP	Right wrist at level of the heart
Wan (2021)[254]	Homemade device (HMD) Bio Pad	PPG, study-developed algorithm	PR	Head
Wang (2022)[255]	Fitbit Charge HR (Fitbit Company, San Francisco, CA)	—	HR	—
Wattanapanyawech (2020)[256]	Apple Watch Series 5 (US), Pillow Automatic Sleep Tracker iOS app (version 3.9.65)	—, Pillow algorithms	HR	Wrist
Weng (2021)[257]	Apple Watch Series 1 (Apple Inc, Cupertino, CA), Corrie Health smartphone app	PPG	HR	Wrist
Weng (2021)[258]	KardiaMobile heart monitor (AliveCor Inc, Mountain View, CA), Kinduct AF platform	1-lead ECG	Cardiac rhythm (SR, AF detection)	—
Wilson-Anumudu (2022)[259]	Cellular-connected BP monitor (BodyTrace Inc.)	—	SBP, DBP	—
Winter (2020)[260]	Pocket-ECG (m-Health Solutions, Burlington, ON)	3-lead ECG	Cardiac rhythm (AF and atrioventricular block detection)	—
Wong (2020)[261]	Polar H10 chest strap	—	HR	Chest
Yamagami (2021)[262]	Fitbit Charge 3, Fitbit smartphone app	—	HR, SpO2	Wrist
Yamakoshi (2021)[263]	Study prototype, Experimental app (Exp_app) usable by iPhone 7 and later (iOS 13.3 or later)	PPG + oscillometry	PR, SBP, DBP	Finger + wrist (swap device attachment)
Yatabe (2021)[264]	HEM-7252G-HP BP monitor (Omron)	Oscillometry	HR, SBP, DBP	Upper left arm
Yen (2022)[265]	ASUS VivoWatch BP (©ASUSTeK Computer), HealthConnect smartphone app	ECG + PPG, VivoWatch algorithm	HR, SBP, DBP	Wrist
	Mi Smart Band 3 (XiaoMi, China), Mi Band mobile app	—	HR	Wrist
Zhang (2020)[266]	Study wireless SpO2 monitor prototype using Texas Instruments Model AFE4490 finger cuff, study-developed smartphone app	2-channel PPG, study-developed algorithm	SpO2	Left- and right-hand index/middle/ring fingers
Zhang (2021)[267]	AMAZFIT® ECG recorder (Huami Corporation, Anhui, China), AMAZFIT® Health App	1-lead ECG	HR, ECG, Cardiac rhythm (arrhythmia detection)	Chest (left side of chest wall) or Wrist (above the ulnar styloid)
Zhang (2022)[268]	HEM-9200T BP monitor (Omron Healthcare, Kyoto, Japan), Gaoxin Health smartphone app	Oscillometry, Omron algorithm	SBP, DBP	Upper arm
Zhang (2021) [269]	HEM-9200T BP monitor (Omron Healthcare, Kyoto Japan), unspecified smartphone app	—	SBP	—
Zhang (2020)[270]	HL868ED BP monitor (Health & Life Co. Ltd, Taipei, Taiwan)	Oscillometry	SBP, DBP	Upper arm
Zhang (2021)[271]	iWRAP theranostic prototype, study-developed smartphone app	Pressure sensor, arterial lumen area (ALA) model	HR, SBP, DBP	Calf: lower limb below the knee
Zhu (2022)[272]	Samsung Galaxy Watch Active 2, MyHeartLab app	PPG, study-developed algorithm	HR	Wrist

If articles do not report the data, the symbol "—" is used.

Abbreviations: AF, atrial fibrillation; aHF, absolute high frequency (measure of HRV) in ms^2; bpm, beats per minute; BP, blood pressure in mmHg; BPM, blood pressure monitoring; CA, California; CE, Conformitè Europëenne (CE) Mark; CNN, convolutional neural network; DBP, diastolic blood pressure; ECG, electrocardiograph(y)/electrocardiogram (recording/tracing); HR, heart rate in bpm; HRV, heart rate variability in ms or n.u.; IBI, inter-beat interval; IL, Illinois; KS, Kansas; LED, light-emitting diode; LLC, limited liability company; MA, Massachusetts; MAP, mean arterial pressure in mmHg; mmHg, millimeters of mercury; MD2K, a Centre of Excellence part of the National Institutes of Health (NIH) Big Data to Knowledge Initiative; $ms^{(2)}$, milliseconds (squared); m/s, metres per second; NJ, New Jersey; (n)HF,

(nighttime) high frequency (measure of HRV) in n.u.; (n)LF, (nighttime) low frequency (measure of HRV) in n.u.; (n)VLF, (nighttime) very low frequency (measure of HRV) in n.u.; n.u., normalized units; OS, operating system; PA, Pennsylvania; PAT, peripheral arterial tonometry; pNN50, percentage of normal R-R intervals that differ by 50 ms (measure of HRV); PPG, photoplethysmography; PR, pulse rate in bpm; PWV, pulse wave velocity in m/s; RMSSD, root mean square of differences in successive R-R intervals (measure of HRV) in ms; RNN, recurrent neural networks; SBP, systolic blood pressure; SDNN, standard deviation of NN intervals (measure of HRV) in ms; SK, South Korea; SpO2, (arterial) (mean) (peripheral) (blood) (capillary) oxygen saturation in percent (%); SR, sinus rhythm; UK, United Kingdom; USA, United States of America; WA, Washington; WCD, wearable cardioverter defibrillator.

Appendix 5. Summary of the cardiovascular variables measured by 216 distinct remote monitoring technologies reported in the 272 included articles.

Remote monitoring technology	Blood pressure (BP), mmHg			Cardiac rhythm	Electrical heart activity (ECG)				Heart rate (HR), bpm	Heart rate variability (HRV), ms	Pulse wave velocity (PWV), m/s	Oxygen saturation (SpO_2), %
	Systolic	Diastolic	Mean arterial		1-lead	2-lead	6-lead	12-lead				
A&D TM-2441[112, 113]	✅	✅							✅			
A&D UA-651BLE BP Monitor[67, 111, 112]	✅	✅										
A&D UA-767PBT-Ci[94]	✅	✅										
A&D UA-772[208]	✅	✅										
Aktiia Bracelet[226, 253]	✅	✅										
AliveCor KardiaMobile®[16, 19, 42, 57, 84, 126, 134, 152, 192, 241, 258]				✅	✅				✅			
AliveCor KardiaMobile® 6L[71, 140]				✅			✅		✅			
AliveCor KardiaBand[214]				✅								
Amazfit ECG recorder[267]				✅	✅				✅			
Amazfit Health Band 1S[38]				✅	✅							
ANSWatch Model TS-0411[144]									✅	✅		
Apple iPhone 4S[58]	✅											
Apple iPhone 6[90]									✅	✅		
Apple iPhone 6S[249]	✅	✅										
Apple iPhone 8[231]									✅	✅		
Apple Watch[45, 215]									✅			
Apple Watch 2 Nike+[122]									✅			
Apple Watch Series 0 [97, 115, 206]									✅			
Apple Watch Series 1[257]									✅			
Apple Watch Series 2 [115, 230]									✅			
Apple Watch Series 3[5, 75, 99]				✅					✅			
Apple Watch Series 4[75, 201]					✅				✅			
Apple Watch Series 5[229, 256]				✅	✅				✅			
Apple Watch Series 6[189, 228]									✅			✅
ASUS VivoWatch BP[125, 265]	✅	✅							✅			
AutoSense chest band[88]										✅		
AViTA BPM65ZB[93]	✅	✅							✅			
Basis B1[109]									✅			
Beat-by-Beat BP Monitor[127]	✅	✅							✅			
Biobeat BB-613WP Wrist Monitor[13, 76]	✅	✅							✅			✅
Biostrap Wristband[69]									✅			✅
BodiMetrics Performance Monitor[82]	✅								✅			✅
BodyTrace BP monitor[259]	✅	✅										
BIC model YK-80A[170]									✅			✅
Biocom Heart Tracker[25]										✅		

Remote monitoring technology	Blood pressure (BP), mmHg			Cardiac rhythm	Electrical heart activity (ECG)				Heart rate (HR), bpm	Heart rate variability (HRV), ms	Pulse wave velocity (PWV), m/s	Oxygen saturation (SpO_2), %
	Systolic	Diastolic	Mean arterial		1-lead	2-lead	6-lead	12-lead				
Biologix Oxistar[188]									✅			✅
Biovotion Everion® bracelet[186, 205]									✅	✅		✅
BPLab device[182]	✅	✅	✅								✅	
Cardiac Designs ECG Check device[8]				✅	✅							
CardiacSense Wristwatch[43, 86]				✅					✅			
CardioMem CM3000[129]				✅		✅			✅			
CardioPoint H100 Holter[248]				✅					✅			
CardioQVARK® iPhone case[74]				✅	✅					✅		
Cardioskin smart T-shirt[63]								✅	✅			
ChoicMMed OxyWatch[190]									✅			✅
Cloud DX BP Monitor[87]	✅	✅							✅			
Coala Heart Monitor[149]				✅								
Contec Medical ABPM50[236]	✅	✅	✅						✅			
Contec CMS50EW pulse oximeter[92]									✅			✅
Contec SAT-300 finger pulse oximeter[10]									✅			✅
Corsano Cardiowatch 287 Bracelet[23]									✅			
Desay Electronics Algoband F8[218]									✅			
ECG247 Smart Heart Sensor[106]				✅	✅							
ECIL Remote Monitoring device[237]									✅			✅
EMAY EMO-80 Pulse Oximeter[34]									✅			✅
Empatica E4 wristband[66]									✅			
Everlast TR10 Watch[82]	✅								✅			
Firstbeat Bodyguard 2 HR Monitor[53, 130]									✅			
Firstbeat Textile ECG Strap[231]									✅	✅		
Fitbit 2[217]									✅			
Fitbit Alta[141]									✅			
Fitbit Alta HR[72, 195];225									✅			
Fitbit Blaze[195]									✅			
Fitbit Charge 2 [15, 121, 145, 158, 178, 198, 199, 219, 225, 232]									✅			
Fitbit Charge 3[83, 108, 172, 232, 262]									✅			✅
Fitbit Charge 4[103, 175]									✅			
Fitbit Charge HR [5, 21, 99, 118, 195, 215, 255]				✅					✅			
Fitbit Charge HR 2[81, 195]									✅			
Fitbit Charge HR 3[195]									✅			
Fitbit Inspire HR[48, 196]									✅	✅		
Fitbit Ionic[183, 195]									✅			
Fitbit Surge[195]									✅			

Remote monitoring technology	Blood pressure (BP), mmHg			Cardiac rhythm	Electrical heart activity (ECG)				Heart rate (HR), bpm	Heart rate variability (HRV), ms	Pulse wave velocity (PWV), m/s	Oxygen saturation (SpO_2), %
	Systolic	Diastolic	Mean arterial		1-lead	2-lead	6-lead	12-lead				
Fitbit Versa[110, 195, 222]									✅			
Fitbit Versa 2[35]									✅			
ForaCare Diamond Cuff[190]	✅	✅							✅			
Garmin Forerunner 945[29]									✅			
Garmin Vívoactive 3[163, 177]									✅			
Garmin Vívofit HR[223]									✅			
Garmin Vívosmart[200]									✅			
Garmin Vívosmart 3[3, 148]									✅	✅		
Garmin Vívosmart 4[136, 243]									✅	✅		
Garmin Vívosmart HR[215]									✅			
Garmin Vívosmart HR+[44, 105]									✅			
Garmin Vívosport™[165]									✅			
Globalcare GCE603 BP Monitor[227]	✅	✅										
HealthSTATS Bpro[161]	✅	✅										
Health&Life HL868ED BP Monitor[270]	✅	✅										
HeartMan wristband[147]									✅			
Hexoskin Smart Shirt[9]									✅			
Huawei Watch 2[197]									✅	✅		
Huawei Watch GT2 Pro ECG edition[143]					✅				✅			✅
iHealth Air PulseOx3M[96]									✅			✅
iHealth Neo BP device[57]	✅	✅							✅			
iHealth Lab BP3L Monitor[216]	✅	✅										
ImageONE Duranta[235]				✅	✅							
InBodyWATCH cuffless device[167]	✅	✅							✅			
Itamar Watch-PAT 200 [59, 100, 104, 180, 209]									✅			✅
iWRAP theranostic prototype[271]	✅	✅							✅			
Kenek Edge pulse oximeter probe[164]									✅			✅
Konica Minolta PULSOX-Me300[173]												✅
Lenovo ZUK Z2 X[28]									✅			✅
Life Plus Dona Care Watch[62]									✅			
LiveMetric LiveOne[211]	✅	✅										
Masimo MightSat[78]												✅
m-Health Solutions Pocket-ECG[260]				✅								
Microlife BP A6 PC BP Monitor[210]	✅	✅		✅					✅			
Microlife BP B3 AFIB Monitor[20]	✅	✅							✅			
Microlife WatchBP-Home A Monitor[73]	✅	✅		✅					✅			
Microlife WatchBP O3 AFIB Monitor[95]	✅	✅		✅					✅			
Mobil-O-Graph BP Monitor[68, 128, 176]	✅	✅	✅						✅			

Remote monitoring technology	Blood pressure (BP), mmHg			Cardiac rhythm	Electrical heart activity (ECG)				Heart rate (HR), bpm	Heart rate variability (HRV), ms	Pulse wave velocity (PWV), m/s	Oxygen saturation (SpO_2), %
	Systolic	Diastolic	Mean arterial		1-lead	2-lead	6-lead	12-lead				
Mobil-O-Graph Arteriograph[51, 116]	✅	✅	✅						✅		✅	
Mobil-O-Graph NG[18, 137]	✅	✅		✅					✅		✅	
Mobil-O-Graph PWA[7, 207]	✅	✅							✅		✅	
MotionSense HRV[132]									✅	✅		
Movesense HR+ Sensor[153]					✅				✅			
Nonin 3230 finger pulse oximeter[60]												✅
Nonin G92 finger pulse oximeter[4]									✅			✅
Nonin Onyx II pulse oximeter[47]												✅
Nonin WristOx₂® Model 3150[4, 204]									✅			✅
Nonin WristOx₂® Model 3250[91]									✅			✅
Nordic Brain Cerebri[98]									✅			
Novacor Diasys 3 Plus[12]	✅	✅							✅			
Nuvo-Group Invu Belt[159]									✅			
Omron Evolv® BPM[41, 75]	✅	✅										
Omron HEM-6410T "HeartGuide"[113, 240]	✅	✅							✅			
Omron HEM-4030[157]	✅	✅										
Omron HEM-7080IC[173, 233, 239]	✅	✅										
Omron HEM-7080-ITZ[213]	✅	✅										
Omron HEM-7080-ITZ2[213]	✅	✅										
Omron HEM-7251G[245]	✅	✅										
Omron HEM-7252G-HP[264]	✅	✅							✅			
Omron HEM-7320T[187]	✅	✅										
Omron HEM-7600T[234]	✅	✅							✅			
Omron HEM-9200T[117, 179, 268, 269]	✅	✅										
Omron M3 Intellisense[11, 155]	✅	✅										
Omron M6 Intellisense[184]	✅	✅										
Omron BP761N 7 Series[187]	✅	✅										
Omron VR BP710N 3 Series[171]	✅	✅										
Oura Ring[31, 169]									✅	✅		
Oura Ring Gen2[124, 154, 231]									✅	✅		
Partron Co. Neofit[123]									✅			
Peloton HR Band[138]									✅			
Petr Telegin BPLab[101]	✅	✅										
Philips Health Watch DL8791[212]									✅			
Polar A360® Activity Tracker[156, 215]									✅			
Polar H7 HR Sensor [9, 15, 32, 39, 65, 89, 162, 178, 250]									✅	✅		

Remote monitoring technology	Blood pressure (BP), mmHg			Cardiac rhythm	Electrical heart activity (ECG)				Heart rate (HR), bpm	Heart rate variability (HRV), ms	Pulse wave velocity (PWV), m/s	Oxygen saturation (SpO_2), %
	Systolic	Diastolic	Mean arterial		1-lead	2-lead	6-lead	12-lead				
Polar H10 HR Sensor [29, 36, 79, 174, 193, 196, 231, 244, 261]									✅	✅		
Polar Ignite Watch[29]									✅			
Polar M430 Watch[17]									✅			
Polar M600 Watch[252]									✅			
Polar OH1 HR Sensor[172]									✅			
Polar RS800CX[26, 120, 247]									✅	✅		
Polar S810i™ pulse watch + Polar chest band[1]									✅			
Polar V800 Watch[52]									✅			
Polar Vantage M Watch[46, 221]									✅			
Preventice BodyGuardian® Heart Monitor[94]									✅			
Pulsesense PS-500B pulsimeter[208]									✅			
Psychorus wristband[220]									✅			
QardioArm Smart BPM[45]	✅	✅							✅			
Recovery Plus HR Band[139]									✅			
Samsung Galaxy phone[114]												✅
Samsung Galaxy Note 9[77]	✅	✅							✅			
Samsung Galaxy S7[55]	✅	✅										
Samsung Galaxy S8[164]									✅			✅
Samsung Galaxy S9[27, 77]	✅	✅							✅			✅
Samsung Galaxy Watch[41]									✅			
Samsung Galaxy Watch Active2[14, 175, 272]	✅	✅		✅					✅			
Samsung Galaxy Watch3 (SM-R850)[2]	✅	✅										
Samsung Gear Sport Watch[133]									✅	✅		
Samsung Gear S3 Watch[37]									✅			
Santiago TM Kit, pulse oximeter[80]									✅			✅
Savvy ECG sensor[251]				✅	✅							
Seers mobiCARE-MC100 ECG patch[131]				✅	✅				✅			
SensiumVitals™ ECG Patch[102]									✅			
Sensogram Sensoscan[78]												✅
Shanghai Berry Pulse Oximeter[11]									✅			✅
Shenzhen Creative PC-68B Wrist Oximeter[204]									✅			✅
Shimmer3 Mobile ECG (mECG)[231]									✅	✅		
Spacelabs BP Monitor[176]	✅	✅							✅			
Spacelabs 90207 BP Monitor[24, 33, 40, 49, 50, 54, 203, 242]	✅	✅							✅			

Remote monitoring technology	Blood pressure (BP), mmHg			Cardiac rhythm	Electrical heart activity (ECG)				Heart rate (HR), bpm	Heart rate variability (HRV), ms	Pulse wave velocity (PWV), m/s	Oxygen saturation (SpO_2), %
	Systolic	Diastolic	Mean arterial		1-lead	2-lead	6-lead	12-lead				
Spacelabs 90217 BP Monitor[33, 142, 181]	✅	✅							✅			
Spacelabs Ultralite 90217[129]	✅	✅										
Spacelabs Lifecard CF[137]				✅					✅			
Spyder ECG monitor[246]				✅	✅							
SunTech OSCAR-2 BPM[176]	✅	✅										
TaiDoc Upright TD-3128 BPM[166, 202]	✅	✅							✅			
TaiDoc FORA P20b[238]	✅	✅										
Thought Technology Triple-Physiology Sensor[64]									✅	✅		
Upmood Band[224]									✅	✅		
Venus Congestion Meter (VenCoM)[151]			✅ (CVP)									
VitalConnect VitalPatch® RTM[168]					✅				✅	✅		
VitalSignum Beat2Phone[146]				✅	✅				✅			
Wavelet Health wristband[214]				✅								
Wellysis S-Patch Cardio Solution[194]				✅	✅				✅			
WHOOP Strap 2.0[22, 160]									✅	✅		
Withings BP Monitor[70, 206, 241]	✅	✅							✅			
Withings Move ECG Watch[150]					✅							
Withings Steel HR[61]									✅			
Xiaomi Mi Band Pulse 1S[119]									✅			
Xiaomi Mi Band 2[44]									✅			
Xiaomi Mi Band 3[56, 265]									✅			
Xiaomi Mi Band 4[53]									✅			
Xiaomi Mi Band 5[103]									✅			
Zephyr BioHarness-3™[185]					✅				✅	✅		
Zephyr BioPatch™ HP[191]					✅				✅			
Zephyr HxM HR Monitor[162]									✅	✅		
ZOLL LifeVest™ WCD[30, 85]						✅			✅	✅		
Zewa UAM-900T BPM[67]	✅	✅										
Zewa UAM-910BT BPM[67]	✅	✅										
Al-Naami prototype[6]									✅			✅
Kaile prototype[107]												✅
Lan prototype[135]									✅			
Mena prototype[157]	✅	✅										
Wan Bio Pad prototype [254]									✅			
Yamakoshi prototype [263]	✅	✅							✅			
Zhang prototype [266]												✅

Abbreviations: ABPM, ambulatory blood pressure monitor; BPM, blood pressure monitor; bpm, beats per minute; CVP, central venous pressure; m/s, metres per second; mmHg, millimetres of mercury; ms, milliseconds; PWA, pulse wave analysis; WCD, wearable cardioverter defibrillator; %, percentage.

Appendix 6. Newcastle-Ottawa Scale quality assessment for observational studies.

First author (year)	Study design	Selection		Comparability		Outcome			Score	Quality
		Q1	Q2	Q3	Q4	Q5	Q6	Q7		
Aljuaid (2020)[8]	R	*	*	*	*	*	*	—	6/9	High
Batta (2022)[18]	L	*	*	*	*	*	*	*	7/9	High
Carnagarin (2022)[33]	L	*	—	*	*	—	*	—	4/9	Low
Chang (2020)[36]	CX	*	**	**	*	*			7/7	High
Chaudhury (2021)[37]	L	—	*	*	*	*	*	*	6/9	High
Chiang (2021)[41]	L	*	*	**	*	*	*	—	7/9	High
Cho (2022)[42]	L	—	**	—	—	*	*	—	4/9	Low
Climstein (2020)[46]	CX	*	*	**	*	*			6/7	High
Garcia-Moreno (2022)[66]	CX	*	**	*	—	*			5/6†	High
Girerd (2022)[70]	L	*	*	—	*	*	*	—	5/9	High
Gkikopoulos (2022)[72]	R	*	—	—	—	*	*	*	4/8†	Low
Gräfitsch (2020)[80]	L	*	—	*	—	—	*	—	3/9	Low
Hahnen (2020)[82]	CX	*	*	—	*	*			4/7	High
Jortveit (2022)[106]	L	*	**	*	*	*	—	—	6/9	High
Khattak (2021)[114]	CX	*	*	*	*	*			5/7	High
Kim (2020)[119]	L	*	*	*	*	*	*	*	7/9	High
Ko (2022)[125]	L	*	**	**	*	*	*	—	8/9	High
Kolkenbeck-Ruh (2022)[128]	CX	*	*	—	*	*			4/7	High
Kuula (2021)[130]	CX	*	*	**	*	—			5/7	High
Kwon (2021)[131]	L	*	**	*	*	*	*	*	8/9	High
Liu (2022)[143]	L	*	—	**	*	*	*	*	7/9	High
Martini (2022)[153]	CX	*	**	—	—	*			4/7	High
Mason (2022)[154]	L	—	—	*	*	*	*	—	4/9	Low
Muggeridge (2021)[172]	CX	*	**	—	*	*			5/7	High
Murase (2022)[173]	L	—	—	*	*	*	*	*	5/9	High
Nabasny (2022)[174]	L	*	**	—	—	*	*	—	5/9	High
Nissen (2022)[175]	CX	*	**	*	—	*			5/7	High
Park (2022)[185]	CX	*	*	*	*	*			5/7	High
Pavic (2020)[186]	L	*	**	*	*	*	*	*	8/9	High
Porumb (2020)[191]	L	*	*	—	*	*	*	—	5/9	High
Rodrigues (2022)[196]	CX	—	**	—	*	*			4/7	High
Saghir (2020)[201]	CX	*	**	*	—	*			5/7	High
Sayer (2022)[211]	L	*	*	**	*	*	*	—	7/9	High
Sher (2022)[217]	L	*	*	*	*	*	*	*	7/9	High
Sola (2021)[226]	CX	—	**	*	—	*			4/7	High
Spaccarotella (2022)[228]	CX	*	*	*	*	*			5/7	High
Tan (2021)[236]	L	*	*	**	*	*	*	*	8/9	High
Toba (2021)[239]	CX	*	*	*	*	*			5/7	High
Tsai (2022)[243]	L	*	*	*	—	*	*	—	5/8†	High
Vischer (2022)[249]	L	—	**	*	*	*	*	—	6/9	High
Wang (2022)[255]	L	—	*	—	*	*	*	—	4/9	Low

If a question receives zero stars, the "—" notation is used.

† If a question is not applicable to the study, a star from the total score is removed.

Abbreviations: CX, cross-sectional; L, longitudinal; R, retrospective.

Appendix 7. Cochrane risk of bias summary per study for randomised controlled trials.

First author (year)	Domain 1 Randomization process	Domain 2 Deviations from the intended interventions	Domain 3 Missing outcome data	Domain 4 Measurement of the outcome	Domain 5 Selection of the reported result	Overall Bias
**Intention to treat**						
Chiang (2021)[41]	+	+	+	+	+	+
Liu (2022)[140]	+	?	−	+	+	−
Marcus (2022)[152]	+	−	+	+	+	−
**Per protocol**						
McNeil (2022)[156]	?	+	+	+	+	?

+ : low risk of bias; ? : some concerns of bias; − : high risk of bias.

Appendix 8. Risk Of Bias In Non-randomised Studies - of Interventions (ROBINS-I) summary per study.

First author (year)	Domain 1 Bias due to confounding	Domain 2 Selection of participants	Domain 3 Classification of interventions	Domain 4 Deviations from the intended interventions	Domain 5 Missing outcome data	Domain 6 Measurement of the outcome	Domain 7 Selection of the reported result	Overall Bias
**Intention to treat**								
Fanget (2022)[62]	?	?	?	+	+	?	+	?
Teo (2021)[238]	+	+	+	+	+	?	+	?
**Per protocol**								
Bolin (2022)[25]	+	+	+	+	+	?	+	?
Larsen (2022)[136]	+	?	+	+	−	?	+	−
Polverino (2022)[190]	+	+	+	+	+	?	+	?
Wilson-Anumudu (2022)[259]	+	+	+	+	?	?	+	?

+ : low risk of bias; ? : moderate risk of bias; − : serious risk of bias.

Appendix 9. Association between the characteristics of the remote monitoring technologies used and the study quality.

	Study quality	
	**High**	**Low**
**Types of technology**		
Photoplethysmography	23	10
Electrocardiography	14	3
Oscillometry	8	5
Pressure sensing	1	0
	$X_3^2(.05) = 2.08$ $p = .56$	
**Cardiovascular variable**		
Heart rate	29	10
Heart rate variability	7	3
Electrocardiogram	9	3
Cardiac rhythm	3	2
Blood pressure	14	5
Oxygen saturation	6	3
Pulse wave velocity	2	0
	$X_6^2(.05) = 1.46$ $p = .96$	
**Wearing location**		
Chest	8	0
Arm	10	5
Wrist	21	6
Finger	1	3
Handheld	4	2
Ear	0	1
	$X_5^2(.05) = 10.8$ $p = .055$	

References

1. Agyün C, Çakir-Atabek H: Alternative Model for Physical Activity: Active Video Games Lead to High Physiological Responses. Res Q Exerc Sport 2021:1-10.
2. Ahn JH, Song J, Choi I, Youn J, Cho JW: Validation of Blood Pressure Measurement Using a Smartwatch in Patients With Parkinson's Disease. Front Neurol 2021, 12:650929.
3. Akbar F, Mark G, Prausnitz S, Warton EM, East JA, Moeller MF, Reed ME, Lieu TA: Physician Stress During Electronic Health Record Inbox Work: In Situ Measurement With Wearable Sensors. JMIR Med Inform 2021, 9:e24014.
4. Al Rajeh AM, Aldabayan YS, Aldhahir A, Pickett E, Quaderi S, Alqahtani JS, Mandal S, Lipman MC, Hurst JR: Once Daily Versus Overnight and Symptom Versus Physiological Monitoring to Detect Exacerbations of Chronic Obstructive Pulmonary Disease: Pilot Randomized Controlled Trial. JMIR Mhealth Uhealth 2020, 8:e17597.
5. Al-Kaisey AM, Koshy AN, Ha FJ, Spencer R, Toner L, Sajeev JK, Teh AW, Farouque O, Lim HS: Accuracy of wrist-worn heart rate monitors for rate control assessment in atrial fibrillation. Int J Cardiol 2020, 300:161-164.
6. Al-Naami B, Abu Owida H, Abu Mallouh M, Al-Naimat F, Agha M, Al-Hinnawi AR: A New Prototype of Smart Wearable Monitoring System Solution for Alzheimer's Patients. Med Devices (Auckl) 2021, 14:423-433.
7. Alexandrou ME, Loutradis C, Balafa O, Theodorakopoulou M, Tzanis G, Bakaloudi D, Dimitriadis C, Pateinakis P, Gkaliagkousi E, Papagianni A, Sarafidis P: Acomparative study of ambulatory central hemodynamics and arterial stiffness parameters in peritoneal dialysis and hemodialysis patients. Journal of Hypertension 2020, 38:2393-2403.
8. Aljuaid M, Marashly Q, AlDanaf J, Tawhari I, Barakat M, Barakat R, Zobell B, Cho W, Chelu MG, Marrouche NF: Smartphone ECG Monitoring System Helps Lower Emergency Room and Clinic Visits in Post-Atrial Fibrillation Ablation Patients. Clin Med Insights Cardiol 2020, 14:1179546820901508.
9. Amelard R, Hedge ET, Hughson RL: Temporal convolutional networks predict dynamic oxygen uptake response from wearable sensors across exercise intensities. NPJ Digit Med 2021, 4:156.
10. Angelucci A, Kuller D, Aliverti A: A Home Telemedicine System for Continuous Respiratory Monitoring. IEEE J Biomed Health Inform 2021, 25:1247-1256.
11. Antali F, Kulin D, Lucz KI, Szabó B, Szűcs L, Kulin S, Miklós Z: Multimodal Assessment of the Pulse Rate Variability Analysis Module of a Photoplethysmography-Based Telemedicine System. Sensors (Basel) 2021, 21:5544.
12. Atkins N, Fania C, Palatini P: Validation of the blood pressure measurement technology used in the Novacor Diasys 3 plus (DIP-0001-00) upper-arm device for ambulatory blood pressure measurement, according to AAMI/ANSI/ISO 81060-2:2013, ESH-IP 2010 and MEDDEV 2.7/1. Blood Pressure Monitoring 2020:359-367.
13. Atzmon Y, Ben Ishay E, Hallak M, Littman R, Eisenkraft A, Gabbay-Benziv R: Continuous Maternal Hemodynamics Monitoring at Delivery Using a Novel, Noninvasive, Wireless,PPG-Based Sensor. J Clin Med 2020, 10.
14. Avram R, Ramsis M, Cristal AD, Nathan V, Zhu L, Kim J, Kuang J, Gao A, Vittinghoff E, Rohdin-Bibby L, et al: Validation of an algorithm for continuous monitoring of atrial fibrillation using a consumer smartwatch. Heart Rhythm 2021, 18:1482-1490.
15. Baek S, Ha Y, Park HW: Accuracy of Wearable Devices for Measuring Heart Rate During Conventional and Nordic Walking. PM R 2021, 13:379-386.
16. Bartlett VL, Ross JS, Shah ND, Ciaccio L, Akar JG, Noseworthy PA, Dhruva SS: Physical activity, patient-reported symptoms, and clinical events: Insights into postprocedural recovery from personal digital devices. Cardiovasc Digit Health J 2021, 2:212-221.
17. Batalik L, Dosbaba F, Hartman M, Konecny V, Batalikova K, Spinar J: Long-term exercise effects after cardiac telerehabilitation in patients with coronary artery disease: 1-year follow-up results of the randomized study. Eur J Phys Rehabil Med 2021, 57:807-814.
18. Batta D, Korosi B, Gyongyosi H, Nemcsik-Bencze Z, Laszlo A, Tisler A, Cseprekal O, Nemcsik J: Cross-sectional comparison of office and ambulatory pulse wave velocity by two methods, and their changes after lifestyle or medical interventions in hypertension. Journal of Hypertension 2022, 40:470-477.
19. Beers L, van Adrichem LP, Himmelreich JCL, Karregat EPM, de Jong J, Postema PG, de Groot JR, Lucassen WAM, Harskamp RE: Manual QT interval measurement with a smartphone-operated single-lead ECG versus 12-lead ECG: a within-patient diagnostic validation study in primary care. BMJ Open 2021, 11:e055072.

20. Beime B, Bramlage C, Kruger R, Deutsch C, Van Mark G, Bramlage P, Botta B: Validation of the Microlife BP B3 AFIB upper arm blood pressure monitor in adults and adolescents according to the ANSI/AAMI/ISO 81060-2:2019 protocol. Blood Pressure Monitoring 2021:299-304.
21. Benedetti D, Olcese U, Frumento P, Bazzani A, Bruno S, d'Ascanio P, Maestri M, Bonanni E, Faraguna U: Heart rate detection by Fitbit ChargeHR™: A validation study versus portable polysomnography. J Sleep Res 2021, 30:e13346.
22. Berryhill S, Morton CJ, Dean A, Berryhill A, Provencio-Dean N, Patel SI, Estep L, Combs D, Mashaqi S, Gerald LB, et al.: Effect of wearables on sleep in healthy individuals: a randomized crossover trial and validation study. Journal of Clinical Sleep Medicine 2020, 16:775–783.
23. Blok S, Piek MA, Tulevski, II, Somsen GA, Winter MM: The accuracy of heartbeat detection using photoplethysmography technology in cardiac patients. J Electrocardiol 2021, 67:148-157.
24. Bohm M, Schwantke I, Mahfoud F, Lauder L, Wagenpfeil S, Sierra AD, Vinyoles E, Gorostidi M, Segura J, Ruilope LM: Association of clinic and ambulatory heart rate parameters with mortality in hypertension. Journal of Hypertension 2020, 38:2416-2426.
25. Bolin LP, Saul AD, Bethune Scroggs LL, Horne C: A pilot study investigating the relationship between heart rate variability and blood pressure in young adults at risk for cardiovascular disease. Clin Hypertens 2022, 28:2.
26. Bourassa S, Best KL, Racine M, Borisoff J, Leblond J, Routhier F: Use of actigraphy to measure real-world physical activities in manual wheelchair users. J Rehabil Assist Technol Eng 2020, 7:2055668320907814.
27. Browne SH, Bernstein M, Bickler PE: Accuracy of Samsung Smartphone Integrated Pulse Oximetry Meets Full FDA Clearance Standards for Clinical Use. medRxiv 2021.
28. Browne SH, Bernstein M, Pan SC, Gonzalez Garcia J, Easson CA, Huang CC, Vaida F: Smartphone Biosensor With App Meets FDA/ISO Standards for Clinical Pulse Oximetry and Can Be Reliably Used by a Wide Range of Patients. Chest 2021, 159:724-732.
29. Budig M, Keiner M, Stoohs R, Hoffmeister M, Höltke V: Heart Rate and Distance Measurement of Two Multisport Activity Trackers and a Cellphone App in Different Sports: A Cross-Sectional Validation and Comparison Field Study. Sensors (Basel) 2021, 22.
30. Burkhoff D, Bailey G, Gimbel JR: Characterization of cardiac acoustic biomarkers in patients with heart failure. Ann Noninvasive Electrocardiol 2020, 25:e12717.
31. Cao R, Azimi I, Sarhaddi F, Niela-Vilen H, Axelin A, Liljeberg P, Rahmani AM: Accuracy Assessment of Oura Ring Nocturnal Heart Rate and Heart Rate Variability in Comparison With Electrocardiography in Time and Frequency Domains: Comprehensive Analysis. J Med Internet Res 2022, 24:e27487.
32. Capdevila L, Castro-Marrero J, Alegre J, Ramos-Castro J, Escorihuela RM: Analysis of Gender Differences in HRV of Patients with Myalgic Encephalomyelitis/Chronic Fatigue Syndrome Using Mobile-Health Technology. Sensors (Basel) 2021, 21:3746.
33. Carnagarin R, Nolde JM, Lee R, Lugo-Gavidia LM, Ward NC, Lambert GW, Lambert EA, Esler MD, Walton A, Kiuchi MG, Schlaich MP: Renal denervation alters ambulatory blood pressure-derived salt sensitivity index in patients with uncontrolled hypertension. Journal of Hypertension 2022, 40:570-578.
34. Castillo-Escario Y, Kumru H, Ferrer-Lluis I, Vidal J, Jané R: Detection of Sleep-Disordered Breathing in Patients with Spinal Cord Injury Using a Smartphone. Sensors (Basel) 2021, 21:7182.
35. Chalmers T, Hickey BA, Newton P, Lin CT, Sibbritt D, McLachlan CS, Clifton-Bligh R, Morley J, Lal S: Stress Watch: The Use of Heart Rate and Heart Rate Variability to Detect Stress: A Pilot Study Using Smart Watch Wearables. Sensors (Basel) 2021, 22.
36. Chang CH, Hsu YJ, Li F, Tu YT, Jhang WL, Hsu CW, Huang CC, Ho CS: Reliability and validity of the physical activity monitor for assessing energy expenditures in sedentary, regularly exercising, non-endurance athlete, and endurance athlete adults. PeerJ 2020, 8:e9717.
37. Chaudhury S, Yu C, Liu R, Kumar K, Hornby S, Duplessis C, Sklar JM, Epstein JE, Reifman J: Wearables Detect Malaria Early in a Controlled Human-Infection Study. IEEE Trans Biomed Eng 2021, 69:2120–2129.
38. Chen E, Jiang J, Su R, Gao M, Zhu S, Zhou J, Huo Y: A new smart wristband equipped with an artificial intelligence algorithm to detect atrial fibrillation. Heart Rhythm 2020, 17:847-853.
39. Chen YS, Lu WA, Pagaduan JC, Kuo CD: A Novel Smartphone App for the Measurement of Ultra-Short-Term and Short-Term Heart Rate Variability: Validity and Reliability Study. JMIR Mhealth Uhealth 2020, 8:e18761.
40. Chesebro AG, Melgarejo JD, Leendertz R, Igwe KC, Lao PJ, Laing KK, Rizvi B, Budge M, Meier IB, Calmon G, et al: White matter hyperintensities mediate the association of nocturnal blood pressure with cognition. Neurology 2020, 94:E1803-E1810.

41. Chiang PH, Wong M, Dey S: Using Wearables and Machine Learning to Enable Personalized Lifestyle Recommendations to Improve Blood Pressure. IEEE J Transl Eng Health Med 2021, 9:2700513.
42. Cho GW, Almeida SO, Gang ES, Elad Y, Duncan R, Budoff MJ, Karlsberg RP: Performance and Integration of Smartphone Wireless ECG Monitoring into the Enterprise Electronic Health Record: First Clinical Experience. Clin Med Insights Case Rep 2022, 15:11795476211069194.
43. Chorin E, Hochstadt A, Schwartz AL, Matz G, Viskin S, Rosso R: Continuous Heart Rate Monitoring for Automatic Detection of Life-Threatening Arrhythmias With Novel Bio-Sensing Technology. Front Cardiovasc Med 2021, 8:707621.
44. Chow HW, Yang CC: Accuracy of Optical Heart Rate Sensing Technology in Wearable Fitness Trackers for Young and Older Adults: Validation and Comparison Study. JMIR Mhealth Uhealth 2020, 8:e14707.
45. Christen T, Nagale S, Reinitz S, Narayanan S, Roy K, Allocco DJ, Osattin A: Using digital health technology to evaluate the impact of chocolate on blood pressure: Results from the COCOA-BP study. Cardiovasc Digit Health J 2020, 1:89-96.
46. Climstein M, Alder JL, Brooker AM, Cartwright EJ, Kemp-Smith K, Simas V, Furness J: Reliability of the Polar Vantage M Sports Watch when Measuring Heart Rate at Different Treadmill Exercise Intensities. Sports (Basel) 2020, 8:117.
47. Cooper CB, Sirichana W, Arnold MT, Neufeld EV, Taylor M, Wang X, Dolezal BA: Remote Patient Monitoring for the Detection of COPD Exacerbations. Int J Chron Obstruct Pulmon Dis 2020, 15:2005-2013.
48. Cos H, Li D, Williams G, Chininis J, Dai R, Zhang J, Srivastava R, Raper L, Sanford D, Hawkins W, et al: Predicting Outcomes in Patients Undergoing Pancreatectomy Using Wearable Technology and Machine Learning: Prospective Cohort Study. J Med Internet Res 2021, 23:e23595.
49. Costa A, D'Angelo A, Ramusino MC, Perini G, Bosone D, Derosa G, Fogari R: Effects of oral administration of alprazolam and lorazepam as hypnotics on cardiovascular parameters in hypertensive patients. Journal of Clinical Psychopharmacology 2021, 41:191-195.
50. Cuspidi C, Paoletti F, Tadic M, Sala C, Gherbesi E, Dell'Oro R, Grassi G, Mancia G: Nocturnal blood pressure: The dark side of white-coat hypertension. Journal of Hypertension 2020, 38:2404-2408.
51. Dagel T, Afsar B, Sag AA, Derin G, Kesim C, Tas AY, Sahin A, Dincer N, Kanbay M: Noninvasive optical coherence tomography imaging correlates with anatomic and physiologic end-organ changes in healthy normotensives with systemic blood pressure variability. Blood Pressure Monitoring 2020:89-94.
52. Davidson P, Düking P, Zinner C, Sperlich B, Hotho A: Smartwatch-Derived Data and Machine Learning Algorithms Estimate Classes of Ratings of Perceived Exertion in Runners: A Pilot Study. Sensors (Basel) 2020, 20:2637.
53. de la Casa Pérez A, Latorre Román PÁ, Muñoz Jiménez M, Lucena Zurita M, Laredo Aguilera JA, Párraga Montilla JA, Cabrera Linares JC: Is the Xiaomi Mi Band 4 an Accuracy Tool for Measuring Health-Related Parameters in Adults and Older People? An Original Validation Study. Int J Environ Res Public Health 2022, 19:1593.
54. De La Sierra A, Mateu A, Gorostidi M, Vinyoles E, Segura J, Ruilope LM: Antihypertensive therapy and short-termblood pressure variability. Journal of Hypertension 2021, 39:349-355.
55. Degott J, Ghajarzadeh-Wurzner A, Hofmann G, Proen √ ßa M, Bonnier G, Lemkaddem A, Lemay M, Christen U, Knebel JF, Durgnat V, et al: Smartphone based blood pressure measurement: accuracy of the OptiBP mobile application according to the AAMI/ESH/ISO universal validation protocol. Blood Press Monit 2021, 26:441-448.
56. Dehghani Zahedani A, Shariat Torbaghan S, Rahili S, Karlin K, Scilley D, Thakkar R, Saberi M, Hashemi N, Perelman D, Aghaeepour N, et al: Improvement in Glucose Regulation Using a Digital Tracker and Continuous Glucose Monitoring in Healthy Adults and Those with Type 2 Diabetes. Diabetes Ther 2021, 12:1871-1886.
57. Dinesen B, Dam Gade J, Skov Schacksen C, Spindler H, Eie Albertsen A, Dittmann L, Jochumsen M, Svenstrup M √ ∏ller D: The Danish Future Patient Telerehabilitation Program for Patients With Atrial Fibrillation: Design and Pilot Study in Collaboration With Patients and Their Spouses. JMIR Cardio 2021, 5:e27321.
58. Dörr M, Weber S, Birkemeyer R, Leonardi L, Winterhalder C, Raichle CJ, Brasier N, Burkard T, Eckstein J: iPhone App compared with standard blood pressure measurement -The iPARR trial. Am Heart J 2021, 233:102-108.
59. Dunietz GL, Sever O, DeRowe A, Tauman R: Sleep position and breathing in late pregnancy and perinatal outcomes. J Clin Sleep Med 2020, 16:955-959.

60. Edwards C, Costello E, Cassidy N, Vick B, Russell AM: Use of the patientMpower App With Home-Based Spirometry to Monitor the Symptoms and Impact of Fibrotic Lung Conditions: Longitudinal Observational Study. JMIR Mhealth Uhealth 2020, 8:e16158.
61. Elzinga WO, Prins S, Borghans L, Gal P, Vargas GA, Groeneveld GJ, Doll RJ: Detection of Clenbuterol-Induced Changes in Heart Rate Using At-Home Recorded Smartwatch Data: Randomized Controlled Trial. JMIR Form Res 2021, 5:e31890.
62. Fanget M, Bayle M, Labeix P, Roche F, Hupin D: Effects of Cardiac Telerehabilitation During COVID-19 on Cardiorespiratory Capacities in Patients With Coronary Artery Disease. Front Physiol 2022, 13:837482.
63. Fouassier D, Roy X, Blanchard A, Hulot JS: Assessment of signal quality measured with a smart 12-lead ECG acquisition T-shirt. Ann Noninvasive Electrocardiol 2020, 25:e12682.
64. Fu D, Serra NI, Mansion H, Mansion ET, Blain-Moraes S: Assessing the Effects of Nature on Physiological States Using Wearable Technologies. Int J Environ Res Public Health 2022, 19:1231.
65. Gambassi BB, Neves VR, Brito EZA, da Silva Fernandes DS, S √ ° CA, da Rocha Nogueira RM, de Jesus Furtado Almeida F, de Ara √ ∫ jo Cavalcanti PA, Gomes Gon √ ßalves ESDC, Neto DS, et al: A validation study of a smartphone application for heart rate variability assessment in asymptomatic adults. Am J Cardiovasc Dis 2020, 10:219-229.
66. Garcia-Moreno FM, Bermudez-Edo M, Rodríguez-García E, Pérez-Mármol JM, Garrido JL, Rodríguez-Fórtiz MJ: A machine learning approach for semi-automatic assessment of IADL dependence in older adults with wearable sensors. Int J Med Inform 2022, 157:104625.
67. Gazit T, Gutman M, Beatty AL: Assessment of Hypertension Control Among Adults Participating in a Mobile Technology Blood Pressure Self-management Program. JAMA Netw Open 2021, 4:e2127008.
68. Georgianos PI, Vaios V, Zebekakis PE, Liakopoulos V: The relation of clinic and ambulatory bp with the risk of cardiovascular events and all-cause mortality among patients on peritoneal dialysis. Journal of Clinical Medicine 2021, 10:2232.
69. Gielen W, Longoria KA, van Mourik RA: Two cases of COVID-19 monitored by a wearable biosensor-a case report. Mhealth 2021, 7:62.
70. Girerd N, Meune C, Duarte K, Vercamer V, Lopez-Sublet M, Mourad JJ: Evidence of a Blood Pressure Reduction During the COVID-19 Pandemic and Associated Lockdown Period: Insights from e-Health Data. Telemed J E Health 2022, 28:266-270.
71. Giudicessi JR, Schram M, Bos JM, Galloway CD, Shreibati JB, Johnson PW, Carter RE, Disrud LW, Kleiman R, Attia ZI, et al: Artificial Intelligence-Enabled Assessment of the Heart Rate Corrected QT Interval Using a Mobile Electrocardiogram Device. Circulation 2021, 143:1274-1286.
72. Gkikopoulos N, Wenger M, Distler O, Becker M: Self-monitoring of the resting heart rate using a fitness tracker smartwatch application leads to an early diagnosis of large vessel vasculitis. BMJ Case Rep 2022, 15:e245021.
73. Gladstone DJ, Wachter R, Schmalstieg-Bahr K, Quinn FR, Hummers E, Ivers N, Marsden T, Thornton A, Djuric A, Suerbaum J, et al.: Screening for Atrial Fibrillation in the Older Population: A Randomized Clinical Trial. JAMA Cardiology 2021, 6:558–567.
74. Gognieva D, Vishnyakova N, Mitina Y, Chomakhidze P, Mesitskaya D, Kuznetsova N, Khiari M, Ryabykina G, Boytsov S, Syrkin A, et al: Remote Screening for Atrial Fibrillation by a Federal Cardiac Monitoring System in Primary Care Patients in Russia: Results from the Prospective Interventional Multicenter FECAS-AFS Study. Glob Heart 2022, 17:4.
75. Golbus JR, Pescatore NA, Nallamothu BK, Shah N, Kheterpal S: Wearable device signals and home blood pressure data across age, sex, race, ethnicity, and clinical phenotypes in the Michigan Predictive Activity & Clinical Trajectories in Health (MIPACT) study: a prospective, community-based observational study. Lancet Digit Health 2021, 3:e707-e715.
76. Goldstein N, Eisenkraft A, Arguello CJ, Yang GJ, Sand E, Ishay AB, Merin R, Fons M, Littman R, Nachman D, Gepner Y: Exploring Early Pre-Symptomatic Detection of Influenza Using Continuous Monitoring of Advanced Physiological Parameters during a Randomized Controlled Trial. J Clin Med 2021, 10.
77. Gordon AM, Mendes WB: A large-scale study of stress, emotions, and blood pressure in daily life using a digital platform. Proc Natl Acad Sci U S A 2021, 118:e2105573118.
78. Gordon WJ, Henderson D, DeSharone A, Fisher HN, Judge J, Levine DM, MacLean L, Sousa D, Su MY, Boxer R: Remote Patient Monitoring Program for Hospital Discharged COVID-19 Patients. Appl Clin Inform 2020, 11:792-801.
79. Goudman L, Brouns R, Linderoth B, Moens M: Effects of Spinal Cord Stimulation on Heart Rate Variability in Patients With Failed Back Surgery Syndrome: Comparison Between a 2-lead ECG and a Wearable Device. Neuromodulation 2021, 24:512-519.

80. Gräfitsch A, Kirchhoff P, Hoffmann H, Staerkle RF, Soysal SD, Glauser PM: Perioperative Tablet-Based Telemonitoring After Abdominal Wall Hernia Surgery: Pilot Prospective Observational Cohort Study. JMIR Perioper Med 2020, 3:e15672.
81. Gresham G, Placencio-Hickok VR, Lauzon M, Nguyen T, Kim H, Mehta S, Paski S, Pandol SJ, Osipov A, Gong J, et al: Feasibility and efficacy of enteral tube feeding on weight stability, lean body mass, and patient-reported outcomes in pancreatic cancer cachexia. J Cachexia Sarcopenia Muscle 2021, 12:1959-1968.
82. Hahnen C, Freeman CG, Haldar N, Hamati JN, Bard DM, Murali V, Merli GJ, Joseph JI, van Helmond N: Accuracy of Vital Signs Measurements by a Smartwatch and a Portable Health Device: Validation Study. JMIR Mhealth Uhealth 2020, 8:e16811.
83. Haveman ME, van Rossum MC, Vaseur RME, van der Riet C, Schuurmann RCL, Hermens HJ, de Vries JPM, Tabak M: Continuous Monitoring of Vital Signs With Wearable Sensors During Daily Life Activities: Validation Study. JMIR Form Res 2022, 6:e30863.
84. Hermans ANL, Gawalko M, Pluymaekers N, Dinh T, Weijs B, van Mourik MJW, Vorstermans B, den Uijl DW, Opsteyn L, Snippe H, et al: Long-term intermittent versus short continuous heart rhythm monitoring for the detection of atrial fibrillation recurrences after catheter ablation. Int J Cardiol 2021, 329:105-112.
85. Hillmann HAK, Hohmann S, Mueller-Leisse J, Zormpas C, Eiringhaus J, Bauersachs J, Veltmann C, Duncker D: Feasibility and First Results of Heart Failure Monitoring Using the Wearable Cardioverter-Defibrillator in Newly Diagnosed Heart Failure with Reduced Ejection Fraction. Sensors (Basel) 2021, 21.
86. Hochstadt A, Havakuk O, Chorin E, Schwartz AL, Merdler I, Laufer M, Lubman N, Ghantous E, Viskin S, Rosso R: Continuous heart rhythm monitoring using mobile photoplethysmography in ambulatory patients. J Electrocardiol 2020, 60:138-141.
87. Hofstede A, Lomme M, Gosselink S, van Drongelen J: The Cloud DX connected HealthKit Pulsewave in home blood pressure monitoring during pregnancy: A clinical evaluation and user experience study. Pregnancy Hypertens 2022, 28:1-8.
88. Hojjatinia S, Daly ER, Hnat T, Hossain SM, Kumar S, Lagoa CM, Nahum-Shani I, Samiei SA, Spring B, Conroy DE: Dynamic models of stress-smoking responses based on high-frequency sensor data. NPJ Digit Med 2021, 4:162.
89. Holgado D, Troya E, Perales JC, Vadillo MA, Sanabria D: Does mental fatigue impair physical performance? A replication study. Eur J Sport Sci 2021, 21:762-770.
90. Holmes CJ, Fedewa MV, Winchester LJ, MacDonald HV, Wind SA, Esco MR: Validity of Smartphone Heart Rate Variability Pre- and Post-Resistance Exercise. Sensors (Basel) 2020, 20:5738.
91. Holmner Å, Öhberg F, Wiklund U, Bergmann E, Blomberg A, Wadell K: How stable is lung function in patients with stable chronic obstructive pulmonary disease when monitored using a telehealth system? A longitudinal and home-based study. BMC Med Inform Decis Mak 2020, 20:87.
92. Holyoke P, Yogaratnam K, Kalles E: Web-Based Smartphone Algorithm for Calculating Blood Pressure From Photoplethysmography Remotely in a General Adult Population: Validation Study. J Med Internet Res 2021, 23:e19187.
93. Huang CC, Chen YH, Hung CS, Lee JK, Hsu TP, Wu HW, Chuang PY, Chen MF, Ho YL: The Association Between Short-term Exposure to Ambient Air Pollution and Patient-Level Home Blood Pressure Among Patients With Chronic Cardiovascular Diseases in a Web-Based Synchronous Telehealth Care Program: Retrospective Study. JMIR Public Health Surveill 2021, 7:e26605.
94. Huang Q, Crumley T, Walters C, Cluckers L, Heirman I, Railkar R, Bhatia G, Cantor M, Benko C, Izmailova ES, et al.: "In-House" Data on the Outside-A Mobile Health Approach. Clinical Pharmacology and Therapeutics 2020, 107:948–956.
95. Huppertz N, Lip GYH, Lane DA: Validation of the modified Microlife blood pressure monitor in patients with paroxysmal atrial fibrillation. Clin Res Cardiol 2020, 109:802-809.
96. Hutchings OR, Dearing C, Jagers D, Shaw MJ, Raffan F, Jones A, Taggart R, Sinclair T, Anderson T, Ritchie AG: Virtual Health Care for Community Management of Patients With COVID-19 in Australia: Observational Cohort Study. J Med Internet Res 2021, 23:e21064.
97. Huynh P, Shan R, Osuji N, Ding J, Isakadze N, Marvel FA, Sharma G, Martin SS: Heart Rate Measurements in Patients with Obstructive Sleep Apnea and Atrial Fibrillation: Prospective Pilot Study Assessing Apple Watch's Agreement With Telemetry Data. JMIR Cardio 2021, 5:e18050.
98. Ingvaldsen SH, Tronvik E, Brenner E, Winnberg I, Olsen A, Gravdahl GB, Stubberud A: A Biofeedback App for Migraine: Development and Usability Study. JMIR Form Res 2021, 5:e23229.
99. Inui T, Kohno H, Kawasaki Y, Matsuura K, Ueda H, Tamura Y, Watanabe M, Inage Y, Yakita Y, Wakabayashi Y, Matsumiya G: Use of a Smart Watch for Early Detection of Paroxysmal Atrial Fibrillation: Validation Study. JMIR Cardio 2020, 4:e14857.

100. Ioachimescu OC, Dholakia SA, Venkateshiah SB, Fields B, Samarghandi A, Anand N, Eisenstein R, Ciavatta MM, Allam JS, Collop NA: Improving the performance of peripheral arterial tonometry-based testing for the diagnosis of obstructive sleep apnea. J Investig Med 2020, 68:1370-1378.
101. Ionov MV, Zhukova OV, Yudina YS, Avdonina NG, Emelyanov IV, Kurapeev DI, Zvartau NE, Konradi AO: Value-based approach to blood pressure telemonitoring and remote counseling in hypertensive patients. Blood Press 2021, 30:20-30.
102. Iqbal FM, Joshi M, Davies G, Khan S, Ashrafian H, Darzi A: The pilot, proof of concept REMOTE-COVID trial: remote monitoring use in suspected cases of COVID-19 (SARS-CoV 2). BMC Public Health 2021, 21:638.
103. Jachymek M, Jachymek MT, Kiedrowicz RM, Kaźmierczak J, Płońska-Gościniak E, Peregud-Pogorzelska M: Wristbands in Home-Based Rehabilitation-Validation of Heart Rate Measurement. Sensors (Basel) 2021, 22:60.
104. Jen R, Orr JE, Li Y, DeYoung P, Smales E, Malhotra A, Owens RL: Accuracy of WatchPAT for the Diagnosis of Obstructive Sleep Apnea in Patients with Chronic Obstructive Pulmonary Disease. Copd 2020, 17:34-39.
105. Jones L, Tan L, Carey-Jones S, Riddell N, Davies R, Brownsdon A, Kelson M, Williams-Thomas R, Busse M, Davies MM, Morgan MPG: Can wearable technology be used to approximate cardiopulmonary exercise testing metrics? Perioper Med (Lond) 2021, 10:9.
106. Jortveit J, Fensli R: Remote ECG Monitoring by ECG247 Smart Heart Sensor. Int J Telemed Appl 2022, 2022:6812889.
107. Kaile K, Fernandez C, Godavarty A: Development of a Smartphone-Based Optical Device to Measure Hemoglobin Concentration Changes for Remote Monitoring of Wounds. Biosensors (Basel) 2021, 11:165.
108. Kalanadhabhatta M, Rahman T, Ganesan D: Effect of Sleep and Biobehavioral Patterns on Multidimensional Cognitive Performance: Longitudinal, In-the-Wild Study. J Med Internet Res 2021, 23:e23936.
109. Kanady JC, Ruoff L, Straus LD, Varbel J, Metzler T, Richards A, Inslicht SS, O'Donovan A, Hlavin J, Neylan TC: Validation of sleep measurement in a multisensor consumer grade wearable device in healthy young adults. J Clin Sleep Med 2020, 16:917-924.
110. Kańtoch E, Kańtoch A: Cardiovascular and Pre-Frailty Risk Assessment during Shelter-In-Place Measures Based on Multimodal Biomarkers Collected from Smart Telemedical Wearables. J Clin Med 2021, 10:1997.
111. Kario K, Nomura A, Harada N, Okura A, Nakagawa K, Tanigawa T, Hida E: Efficacy of a digital therapeutics system in the management of essential hypertension: the HERB-DH1 pivotal trial. Eur Heart J 2021, 42:4111-4122.
112. Kario K, Nomura A, Kato A, Harada N, Tanigawa T, So R, Suzuki S, Hida E, Satake K: Digital therapeutics for essential hypertension using a smartphone application: A randomized, open-label, multicenter pilot study. J Clin Hypertens (Greenwich) 2021, 23:923-934.
113. Kario K, Shimbo D, Tomitani N, Kanegae H, Schwartz JE, Williams B: The first study comparing a wearable watch-type blood pressure monitor with a conventional ambulatory blood pressure monitor on in-office and out-of-office settings. J Clin Hypertens (Greenwich) 2020, 22:135-141.
114. Khattak AF, Kakakhel SS, Wazir NK, Khattak M, Khattak T, Akbar F: Reliability of Smartphone Applications for the Quantification of Oxygen Saturation. Cureus 2021, 13:e19417.
115. Khushhal A, Nichols S, Carroll S, Abt G, Ingle L: Characterising the application of the "progressive overload" principle of exercise training within cardiac rehabilitation: A United Kingdom-based community programme. PLoS One 2020, 15:e0237197.
116. Kilic A, Baydar O, Elcioglu BC, Camkiran V, Apaydin Z, Can MM, Elcik D: Understanding Vascular Age: Are Clinical scoring systems useful for Early Vascular Aging Syndrome Prediction ? High Blood Pressure and Cardiovascular Prevention 2020, 27:569-577.
117. Kim BJ, Park JM, Park TH, Kim J, Lee J, Lee KJ, Lee J, Chae JE, Thabane L, Lee J, Bae HJ: Remote blood pressure monitoring and behavioral intensification for stroke: A randomized controlled feasibility trial. PLoS One 2020, 15:e0229483.
118. Kim B, Hunt M, Muscedere J, Maslove DM, Lee J: Using Consumer-Grade Physical Activity Trackers to Measure Frailty Transitions in Older Critical Care Survivors: Exploratory Observational Study. JMIR Aging 2021, 4:e19859.
119. Kim B, McKay SM, Lee J: Consumer-Grade Wearable Device for Predicting Frailty in Canadian Home Care Service Clients: Prospective Observational Proof-of-Concept Study. J Med Internet Res 2020, 22:e19732.
120. Kim CS, Kim M, Kim MJ, Jung H: Effects of sleep-inducing juice on sleep quality and heart rate variability in adults with disturbed sleep. Nutrition Research and Practice 2020, 14:606–620.

121. Kim KH, Lee J, Ahn CH, Yu HW, Choi JY, Lee HY, Lee WW, Moon JH: Association between Thyroid Function and Heart Rate Monitored by Wearable Devices in Patients with Hypothyroidism. Endocrinol Metab (Seoul) 2021, 36:1121-1130.
122. Kim K, Kim TW, Kim SH: Analysis of physiological data from long- term physical exercise: A basis for improving the health of rural residents of Korea. Technol Health Care 2020, 28:253-262.
123. Kim Y, Seo J, An SY, Sinn DH, Hwang JH: Efficacy and Safety of an mHealth App and Wearable Device in Physical Performance for Patients With Hepatocellular Carcinoma: Development and Usability Study. JMIR Mhealth Uhealth 2020, 8:e14435.
124. Kinnunen H, Rantanen A, Kenttä T, Koskimäki H: Feasible assessment of recovery and cardiovascular health: accuracy of nocturnal HR and HRV assessed via ring PPG in comparison to medical grade ECG. Physiol Meas 2020, 41:04NT01.
125. Ko YF, Kuo PH, Wang CF, Chen YJ, Chuang PC, Li SZ, Chen BW, Yang FC, Lo YC, Yang Y, et al: Quantification Analysis of Sleep Based on Smartwatch Sensors for Parkinson's Disease. Biosensors (Basel) 2022, 12:74.
126. Koh KT, Law WC, Zaw WM, Foo DHP, Tan CT, Steven A, Samuel D, Fam TL, Chai CH, Wong ZS, et al.: Smartphone electrocardiogram for detecting atrial fibrillation after a cerebral ischaemic event: a multicentre randomized controlled trial. Europace 2021, 23:1016–1023.
127. Kokubo A, Kuwabara M, Nakajima H, Tomitani N, Yamashita S, Shiga T, Kario K: Automatic detection algorithm for establishing standard to identify "surge blood pressure". Med Biol Eng Comput 2020, 58:1393-1404.
128. Kolkenbeck-Ruh A, Soepnel LM, Kim AW, Naidoo S, Smith W, Davies J, Ware LJ: Pulsewave velocity in South Africanwomen and children: Comparison between theMobil-O-Graph and SphygmoCor XCEL devices. Journal of Hypertension 2022, 40:65-75.
129. Krhut J, Wohlfahrt P, Pudich J, Kufova E, Borovicka V, Bilkova K, Sykora R, Mokris J, Cifkova R, Zachoval R, Zvara P: Cardiovascular safety of mirabegron in individuals treated for spinal cord injury- or multiple sclerosis-induced neurogenic detrusor overactivity. International Urology and Nephrology 2021, 53:1089-1095.
130. Kuula L, Pesonen AK: Heart Rate Variability and Firstbeat Method for Detecting Sleep Stages in Healthy Young Adults: Feasibility Study. JMIR Mhealth Uhealth 2021, 9:e24704.
131. Kwon S, Lee SR, Choi EK, Ahn HJ, Song HS, Lee YS, Oh S: Validation of Adhesive Single-Lead ECG Device Compared with Holter Monitoring among Non-Atrial Fibrillation Patients. Sensors (Basel) 2021, 21:3122.
132. Kwon S, Wan N, Burns RD, Brusseau TA, Kim Y, Kumar S, Ertin E, Wetter DW, Lam CY, Wen M, Byun W: The Validity of MotionSense HRV in Estimating Sedentary Behavior and Physical Activity under Free-Living and Simulated Activity Settings. Sensors (Basel) 2021, 21:1411.
133. Lai J, Rahmani A, Yunusova A, Rivera AP, Labbaf S, Hu S, Dutt N, Jain R, Borelli JL: Using Multimodal Assessments to Capture Personalized Contexts of College Student Well-being in 2020: Case Study. JMIR Form Res 2021, 5:e26186.
134. Lambert CT, Patel D, Bumgarner JM, Kanj M, Cantillon D, Saliba W, Hussein A, Baranowski B, Dresing T, Chung MK, et al: Atrial fibrillation future clinic. Novel platform to integrate smart device electrocardiogram into clinical practice. Cardiovasc Digit Health J 2021, 2:92-100.
135. Lan KC, Litscher G, Hung TH: Traditional Chinese Medicine Pulse Diagnosis on a Smartphone Using Skin Impedance at Acupoints: A Feasibility Study. Sensors (Basel) 2020, 20:4618.
136. Larsen LH, Lauritzen MH, Sinkjaer M, Kjaer TW: The Effect of Wearable Tracking Devices on Cardiorespiratory Fitness Among Inactive Adults: Crossover Study. JMIR Cardio 2022, 6:e31501.
137. Lauder L, Scholz SS, Ewen S, Lettner C, Ukena C, Bohm M, Mahfoud F: Accuracy of pulse rate derived from24-h ambulatory blood pressuremonitoring compared with heart rate from 24-h Holter-ECG. Journal of Hypertension 2020, 38:2387-2392.
138. Layton AM, Irwin AM, Mihalik EC, Fleisch E, Keating CL, DiMango EA, Shah L, Arcasoy SM: Telerehabilitation Using Fitness Application in Patients with Severe Cystic Fibrosis Awaiting Lung Transplant: A Pilot Study. Int J Telemed Appl 2021, 2021:6641853.
139. Li J, Wei D, Liu S, Li M, Chen X, Chen L, Wu Y, Zhou W, Ouyang L, Tan C, et al.: Efficiency of an mHealth App and Chest-Wearable Remote Exercise Monitoring Intervention in Patients With Type 2 Diabetes: a Prospective, Multicenter Randomized Controlled Trial. JMIR mHealth and uHealth 2021, 9:e23338.
140. Liu HH, Ezekowitz MD, Columbo M, Khan O, Martin J, Spahr J, Yaron D, Cushinotto L, Kapelusznik L: Testing the feasibility of operationalizing a prospective, randomized trial with remote cardiac safety EKG monitoring during a pandemic. J Interv Card Electrophysiol 2022, 63:345-356.

141. Liu J, Zhao Y, Lai B, Wang H, Tsui KL: Wearable Device Heart Rate and Activity Data in an Unsupervised Approach to Personalized Sleep Monitoring: Algorithm Validation. JMIR Mhealth Uhealth 2020, 8:e18370.
142. Liu W, Ye Y, Gao C, Bai Y, Chu H, Fan W, Sun Z, Wang L, Li X, Yang J: Central versus ambulatory blood pressure for predictingmortality and cardiovascular events in hemodialysis patients: Amulticenter cohort study. Journal of Hypertension 2022, 40:180-188.
143. Liu X, Fan J, Guo Y, Dai H, Xu J, Wang L, Hu P, Lin X, Li C, Zhou D, et al: Wearable Smartwatch Facilitated Remote Health Management for Patients Undergoing Transcatheter Aortic Valve Replacement. J Am Heart Assoc 2022, 11:e023219.
144. Lo EWV, Wei YH, Hwang BF: Association between occupational burnout and heart rate variability: A pilot study in a high-tech company in Taiwan. Medicine (United States) 2020, 99:e18630.
145. Low CA, Li M, Vega J, Durica KC, Ferreira D, Tam V, Hogg M, Zeh Iii H, Doryab A, Dey AK: Digital Biomarkers of Symptom Burden Self-Reported by Perioperative Patients Undergoing Pancreatic Surgery: Prospective Longitudinal Study. JMIR Cancer 2021, 7:e27975.
146. Lumikari TJ, Pirinen J, Putaala J, Sibolt G, Kerola A, Pakarinen S, Lehto M, Nieminen T: Prolonged ECG with a novel recorder utilizing electrode belt and mobile device in patients with recent embolic stroke of undetermined source: A pilot study. Ann Noninvasive Electrocardiol 2020, 25:e12802.
147. Luštrek M, Bohanec M, Cavero Barca C, Ciancarelli MC, Clays E, Dawodu AA, Derboven J, De Smedt D, Dovgan E, Lampe J, et al: A Personal Health System for Self-Management of Congestive Heart Failure (HeartMan): Development, Technical Evaluation, and Proof-of-Concept Randomized Controlled Trial. JMIR Med Inform 2021, 9:e24501.
148. Mach M, Watzal V, Hasan W, Andreas M, Winkler B, Weiss G, Strouhal A, Adlbrecht C, Delle Karth G, Grabenw √ ∂ger M: Fitness-Tracker Assisted Frailty-Assessment Before Transcatheter Aortic Valve Implantation: Proof-of-Concept Study. JMIR Mhealth Uhealth 2020, 8:e19227.
149. Magnusson P, Lyren A, Mattsson G: Diagnostic yield of chest and thumb ECG after cryptogenic stroke, Transient ECG Assessment in Stroke Evaluation (TEASE): an observational trial. BMJ Open 2020, 10:e037573.
150. Maille B, Wilkin M, Million M, Ress √ ©guier N, Franceschi F, Koutbi-Franceschi L, Hourdain J, Martinez E, Zabern M, Gardella C, et al: Smartwatch Electrocardiogram and Artificial Intelligence for Assessing Cardiac-Rhythm Safety of Drug Therapy in the COVID-19 Pandemic. The QT-logs study. Int J Cardiol 2021, 331:333-339.
151. Marcelli E, Cercenelli L, Bortolani B, Marini S, Arfilli L, Capucci A, Plicchi G: A novel non-invasive device for the assessment of central venous pressure in hospital, office and home. Medical Devices: Evidence and Research 2021, 14:141-154.
152. Marcus GM, Modrow MF, Schmid CH, Sigona K, Nah G, Yang J, Chu TC, Joyce S, Gettabecha S, Ogomori K, et al.: Individualized Studies of Triggers of Paroxysmal Atrial Fibrillation: The I-STOP-AFib Randomized Clinical Trial. JAMA Cardiology 2022, 7:167–174.
153. Martini C, Di Maria B, Reverberi C, Tuttolomondo D, Gaibazzi N: Commercially Available Heart Rate Monitor Repurposed for Automatic Arrhythmia Detection with Snapshot Electrocardiographic Capability: A Pilot Validation. Diagnostics (Basel) 2022, 12:712.
154. Mason AE, Kasl P, Hartogensis W, Natale JL, Dilchert S, Dasgupta S, Purawat S, Chowdhary A, Anglo C, Veasna D, et al: Metrics from Wearable Devices as Candidate Predictors of Antibody Response Following Vaccination against COVID-19: Data from the Second TemPredict Study. Vaccines (Basel) 2022, 10.
155. McManus RJ, Little P, Stuart B, Morton K, Raftery J, Kelly J, Bradbury K, Zhang J, Zhu S, Murray E, et al.: Home and Online Management and Evaluation of Blood Pressure (HOME BP) using a digital intervention in poorly controlled hypertension: randomised controlled trial. BMJ (Clinical research ed) 2021, 372:m4858.
156. McNeil J, Fahim M, Stone CR, O'Reilly R, Courneya KS, Friedenreich CM: Adherence to a lower versus higher intensity physical activity intervention in the Breast Cancer & Physical Activity Level (BC-PAL) Trial. J Cancer Surviv 2022, 16:353-365.
157. Mena LJ, Félix VG, Ostos R, González AJ, Martínez-Peláez R, Melgarejo JD, Maestre GE: Mobile Personal Health Care System for Noninvasive, Pervasive, and Continuous Blood Pressure Monitoring: Development and Usability Study. JMIR Mhealth Uhealth 2020, 8:e18012.
158. Meng Y, Speier W, Shufelt C, Joung S, J EVE, Bairey Merz CN, Lopez M, Spiegel B, Arnold CW: A Machine Learning Approach to Classifying Self-Reported Health Status in a Cohort of Patients With Heart Disease Using Activity Tracker Data. IEEE J Biomed Health Inform 2020, 24:878-884.
159. Mhajna M, Schwartz N, Levit-Rosen L, Warsof S, Lipschuetz M, Jakobs M, Rychik J, Sohn C, Yagel S: Wireless, remote solution for home fetal and maternal heart rate monitoring. Am J Obstet Gynecol MFM 2020, 2:100101.

160. Miller DJ, Lastella M, Scanlan AT, Bellenger C, Halson SL, Roach GD, Sargent C: A validation study of the WHOOP strap against polysomnography to assess sleep. J Sports Sci 2020, 38:2631-2636.
161. Miranda Hurtado M, Reyes Vasquez J, Rodriguez-Fernandez M: Comparison of a tonometric with an oscillometric blood pressure monitoring device over 24 hours of ambulatory use. Blood Press Monit 2021, 26:149-155.
162. Mishra V, Pope G, Lord S, Lewia S, Lowens B, Caine K, Sen S, Halter R, Kotz D: Continuous Detection of Physiological Stress with Commodity Hardware. ACM Trans Comput Healthc 2020, 1:8:1–8:30.
163. Mocny-Pachońska K, Doniec R, Trzcionka A, Pacho≈Ñski M, Piaseczna N, Sieci≈Ñski S, Osadcha O, ≈Åanowy P, Tanasiewicz M: Evaluating the stress-response of dental students to the dental school environment. PeerJ 2020, 8:e8981.
164. Modi AM, Kiourkas RD, Li J, Scott JB: Reliability of Smartphone Pulse Oximetry in Subjects at Risk for Hypoxemia. Respir Care 2021, 66:384-390.
165. Moitinho-Silva L, Wegener M, May S, Schrinner F, Akhtar A, Boysen TJ, Schaeffer E, Hansen C, Schmidt T, R√ºhlemann MC, et al.: Short-term physical exercise impacts on the human holobiont obtained by a randomised intervention study. BMC Microbiology 2021, 21:162.
166. Montrivade S, Chattranukulchai P, Siwamogsatham S, Vorasettakarnkij Y, Naeowong W, Boonchayaanant P, Sakulsupsiri A, Ariyachaipanich A, Lertsuwunseri V, Rungpradubvong V, et al: Hypertension Subtypes among Thai Hypertensives: An Analysis of Telehealth-Assisted Instrument in Home Blood Pressure Monitoring Nationwide Pilot Project. Int J Hypertens 2020, 2020:3261408.
167. Moon JH, Kang MK, Choi CE, Min J, Lee HY, Lim S: Validation of a wearable cuff-less wristwatch-type blood pressure monitoring device. Sci Rep 2020, 10:19015.
168. Morgado Areia C, Santos M, Vollam S, Pimentel M, Young L, Roman C, Ede J, Piper P, King E, Gustafson O, et al: A Chest Patch for Continuous Vital Sign Monitoring: Clinical Validation Study During Movement and Controlled Hypoxia. J Med Internet Res 2021, 23:e27547.
169. Moshe I, Terhorst Y, Opoku Asare K, Sander LB, Ferreira D, Baumeister H, Mohr DC, Pulkki-R√•back L: Predicting Symptoms of Depression and Anxiety Using Smartphone and Wearable Data. Front Psychiatry 2021, 12:625247.
170. Motta LP, Silva P, Borguezan BM, Amaral J, Milagres LG, B√≥ia MN, Ferraz MR, Mogami R, Nunes RA, Melo PL: An emergency system for monitoring pulse oximetry, peak expiratory flow, and body temperature of patients with COVID-19 at home: Development and preliminary application. PLoS One 2021, 16:e0247635.
171. Mugabirwe B, Flickinger T, Cox L, Ariho P, Dillingham R, Okello S: Acceptability and feasibility of a mobile health application for blood pressure monitoring in rural Uganda. JAMIA Open 2021, 4:ooaa068.
172. Muggeridge DJ, Hickson K, Davies AV, Giggins OM, Megson IL, Gorely T, Crabtree DR: Measurement of Heart Rate Using the Polar OH1 and Fitbit Charge 3 Wearable Devices in Healthy Adults During Light, Moderate, Vigorous, and Sprint-Based Exercise: Validation Study. JMIR Mhealth Uhealth 2021, 9:e25313.
173. Murase K, Matsumoto T, Tabara Y, Ohler A, Gozal D, Minami T, Kanai O, Takeyama H, Takahashi N, Hamada S, et al: Association of Sleep-disordered Breathing and Blood Pressure with Albuminuria: The Nagahama Study. Ann Am Thorac Soc 2022, 19:451-461.
174. Nabasny A, Rabinowitz A, Wright B, Wang J, Preminger S, Terhorst L, Juengst SB: Neurobehavioral Symptoms and Heart Rate Variability: Feasibility of Remote Collection Using Mobile Health Technology. J Head Trauma Rehabil 2022, 37:178–188.
175. Nissen M, Slim S, J√§ger K, Flaucher M, Huebner H, Danzberger N, Fasching PA, Beckmann MW, Gradl S, Eskofier BM: Heart Rate Measurement Accuracy of Fitbit Charge 4 and Samsung Galaxy Watch Active2: Device Evaluation Study. JMIR Form Res 2022, 6:e33635.
176. Nolde JM, Kiuchi MG, Lugo-Gavidia LM, Ho JK, Chan J, Matthews VB, Herat LY, Carnagarin R, Azzam O, Schlaich MP: Nocturnal hypertension: A common phenotype in a tertiary clinical setting associatedwith increased arterial stiffness and central blood pressure. Journal of Hypertension 2021, 39:250-258.
177. Ocagli H, Lorenzoni G, Lanera C, Schiavo A, D'Angelo L, Liberti AD, Besola L, Cibin G, Martinato M, Azzolina D, et al: Monitoring Patients Reported Outcomes after Valve Replacement Using Wearable Devices: Insights on Feasibility and Capability Study: Feasibility Results. Int J Environ Res Public Health 2021, 18:7171.
178. O'Driscoll R, Turicchi J, Hopkins M, Gibbons C, Larsen SC, Palmeira AL, Heitmann BL, Horgan GW, Finlayson G, Stubbs RJ: The validity of two widely used commercial and research-grade activity monitors, during resting, household and activity behaviours. Health and Technology 2020, 10:637–648.

179. Oh SW, Kim KK, Kim SS, Park SK, Park S: Effect of an Integrative Mobile Health Intervention in Patients With Hypertension and Diabetes: Crossover Study. JMIR Mhealth Uhealth 2022, 10:e27192.
180. Ohtsuka K, Baba R, Yamasawa W, Shirahama R, Hattori Y, Senoura H, Betsuyaku T, Fukunaga K: The Effectiveness of Nasal Airway Stent Therapy for the Treatment of Mild-to-Moderate Obstructive Sleep Apnea Syndrome. Respiration 2021, 100:193-200.
181. Olbers J, Ostergren J, Rosenqvist M, Skuladottir H, Klaveback S, Ljungman P, Witt N: Changes in 24-h ambulatory blood pressure following restoration of sinus rhythmin patientswith atrial fibrillation. Journal of Hypertension 2021, 39:243-249.
182. Omboni S, Posokhov I, Parati G, Arystan A, Tan I, Barkan V, Bulanova N, Derevyanchenko M, Grigoricheva E, Minyukhina I, et al: Variable association of 24-h peripheral and central hemodynamics and stiffness with hypertension-mediated organ damage: the VASOTENS Registry. J Hypertens 2020, 38:701-715.
183. Ong JL, Lau T, Massar SAA, Chong ZT, Ng BKL, Koek D, Zhao W, Yeo BTT, Cheong K, Chee MWL: COVID-19-related mobility reduction: heterogenous effects on sleep and physical activity rhythms. Sleep 2021, 44:1–13.
184. Panula T, Koivisto T, Pänkäälä M, Niiranen T, Kantola I, Kaisti M: An instrument for measuring blood pressure and assessing cardiovascular health from the fingertip. Biosens Bioelectron 2020, 167:112483.
185. Park SC, Saiphoklang N, Jung D, Gomez D, Phillips JE, Dolezal BA, Tashkin DP, Barjaktarevic I, Cooper CB: Use of a Wearable Biosensor to Study Heart Rate Variability in Chronic Obstructive Pulmonary Disease and Its Relationship to Disease Severity. Sensors (Basel) 2022, 22:2264.
186. Pavic M, Klaas V, Theile G, Kraft J, Tr √ ∂ster G, Blum D, Guckenberger M: Mobile Health Technologies for Continuous Monitoring of Cancer Patients in Palliative Care Aiming to Predict Health Status Deterioration: A Feasibility Study. J Palliat Med 2020, 23:678-685.
187. Persell SD, Peprah YA, Lipiszko D, Lee JY, Li JJ, Ciolino JD, Karmali KN, Sato H: Effect of Home Blood Pressure Monitoring via a Smartphone Hypertension Coaching Application or Tracking Application on Adults With Uncontrolled Hypertension: A Randomized Clinical Trial. JAMA Netw Open 2020, 3:e200255.
188. Pinheiro GDL, Cruz AF, Domingues DM, Genta PR, Drager LF, Strollo PJ, Lorenzi-Filho G: Validation of an Overnight Wireless High-Resolution Oximeter plus Cloud-Based Algorithm for the Diagnosis of Obstructive Sleep Apnea. Clinics (Sao Paulo) 2020, 75:e2414.
189. Pipek LZ, Nascimento RFV, Acencio MMP, Teixeira LR: Comparison of SpO(2) and heart rate values on Apple Watch and conventional commercial oximeters devices in patients with lung disease. Sci Rep 2021, 11:18901.
190. Polverino P, Ajčević M, Catalan M, Bertolotti C, Furlanis G, Marsich A, Buoite Stella A, Accardo A, Manganotti P: Comprehensive telemedicine solution for remote monitoring of Parkinson's disease patients with orthostatic hypotension during COVID-19 pandemic. Neurol Sci 2022, 43:3479–3487.
191. Porumb M, Stranges S, Pescapè A, Pecchia L: Precision Medicine and Artificial Intelligence: A Pilot Study on Deep Learning for Hypoglycemic Events Detection based on ECG. Sci Rep 2020, 10:170.
192. Praus T, Li J, Barbarash S, Proenza M, Bondmass MD: Improving care for patients with atrial fibrillation through the use of a personal electrocardiogram. J Am Assoc Nurse Pract 2021, 33:1307-1313.
193. Prigent G, Aminian K, Rodrigues T, Vesin JM, Millet GP, Falbriard M, Meyer F, Paraschiv-Ionescu A: Indirect Estimation of Breathing Rate from Heart Rate Monitoring System during Running. Sensors (Basel) 2021, 21:5651.
194. Puhr-Westerheide D, Kostbade T, Clevert DA: Advantage and use of S-patch cardio solution in competitive motor sports. Clin Hemorheol Microcirc 2020, 74:13-19.
195. Quer G, Gouda P, Galarnyk M, Topol EJ, Steinhubl SR: Inter- and intraindividual variability in daily resting heart rate and its associations with age, sex, sleep, BMI, and time of year: Retrospective, longitudinal cohort study of 92,457 adults. PLoS One 2020, 15:e0227709.
196. Rodrigues E, Lima D, Barbosa P, Gonzaga K, Guerra RO, Pimentel M, Barbosa H, Maciel Å: HRV Monitoring Using Commercial Wearable Devices as a Health Indicator for Older Persons during the Pandemic. Sensors (Basel) 2022, 22:2001.
197. Roe J, Mondschein A, Neale C, Barnes L, Boukhechba M, Lopez S: The Urban Built Environment, Walking and Mental Health Outcomes Among Older Adults: A Pilot Study. Front Public Health 2020, 8:575946.
198. Rykov Y, Thach TQ, Bojic I, Christopoulos G, Car J: Digital Biomarkers for Depression Screening With Wearable Devices: Cross-sectional Study With Machine Learning Modeling. JMIR Mhealth Uhealth 2021, 9:e24872.

199. Rykov Y, Thach TQ, Dunleavy G, Roberts AC, Christopoulos G, Soh CK, Car J: Activity Tracker-Based Metrics as Digital Markers of Cardiometabolic Health in Working Adults: Cross-Sectional Study. JMIR Mhealth Uhealth 2020, 8:e16409.
200. Saarikko J, Niela-Vilen H, Ekholm E, Hamari L, Azimi I, Liljeberg P, Rahmani AM, L √ ∂yttyniemi E, Axelin A: Continuous 7-Month Internet of Things-Based Monitoring of Health Parameters of Pregnant and Postpartum Women: Prospective Observational Feasibility Study. JMIR Form Res 2020, 4:e12417.
201. Saghir N, Aggarwal A, Soneji N, Valencia V, Rodgers G, Kurian T: A comparison of manual electrocardiographic interval and waveform analysis in lead 1 of 12-lead ECG and Apple Watch ECG: A validation study. Cardiovasc Digit Health J 2020, 1:30-36.
202. Sakulsupsiri A, Chattranukulchai P, Siwamogsatham S, Boonchayaanant P, Naeowong W, Ariyachaipanich A, Lertsuwunseri V, Rungpradubvong V, Satitthummanid S, Puwanant S, et al: Home Blood Pressure Control and Drug Prescription Patterns among Thai Hypertensives: A 1-Year Analysis of Telehealth Assisted Instrument in Home Blood Pressure Monitoring Nationwide Pilot Project. Int J Hypertens 2021, 2021:8844727.
203. Salazar MR, Espeche WG, Balbin E, Leiva Sisnieguez CE, Minetto J, Leiva Sisnieguez BC, MacIel PM, Stavile RN, Carbajal HA: Prevalence of isolated nocturnal hypertension according to 2018 European Society of Cardiology and European Society of Hypertension office blood pressure categories. Journal of Hypertension 2020, 38:434-440.
204. Salvi D, Poffley E, Tarassenko L, Orchard E: App-Based Versus Standard Six-Minute Walk Test in Pulmonary Hypertension: Mixed Methods Study. JMIR Mhealth Uhealth 2021, 9:e22748.
205. Saner H, Schütz N, Botros A, Urwyler P, Buluschek P, du Pasquier G, Nef T: Potential of Ambient Sensor Systems for Early Detection of Health Problems in Older Adults. Front Cardiovasc Med 2020, 7:110.
206. Sardana M, Lin H, Zhang Y, Liu C, Trinquart L, Benjamin EJ, Manders ES, Fusco K, Kornej J, Hammond MM, et al: Association of Habitual Physical Activity With Home Blood Pressure in the Electronic Framingham Heart Study (eFHS): Cross-sectional Study. J Med Internet Res 2021, 23:e25591.
207. Sarganas G, Kuhnert R, Gohlisch C, van der Giet M, Neuhauser H: Comparison of two blood pressure oscillometric devices: Datascope Accutorr plus and Mobil-O-Graph PWA and conversion of blood pressure values from one device to the other. Blood Pressure Monitoring 2020:42-49.
208. Sasaki-Otomaru A, Yamasue K, Tochikubo O, Saito K, Inamori M: Association of home blood pressure with sleep and physical and mental activity, assessed via a wristwatch-type pulsimeter with accelerometer in adults. Clin Exp Hypertens 2020, 42:131-138.
209. Sato S, Saito J, Fukuhara A, Uematsu M, Suzuki Y, Rikimaru M, Kawamata T, Umeda T, Koizumi T, Togawa R, et al: Association Between Sleep Characteristics and Asthma Control in Middle-Aged and Older Adults: A Prospective Cohort Study. J Asthma Allergy 2021, 14:325-334.
210. Savvari P, Triantafyllidi H, Skiadas J, Kalogeropoulos P, Menegas D, Manolis A, Papoulidis N, Andrikopoulos G, Tsioufis K: Increased detection of suspected atrial fibrillation in elderly and female hypertensive patients through home blood pressure monitoring: The HOME-AF study. Journal of Hypertension 2020, 38:441-447.
211. Sayer G, Piper G, Vorovich E, Raikhelkar J, Kim GH, Rodgers D, Shimbo D, Uriel N: Continuous Monitoring of Blood Pressure Using a Wrist-Worn Cuffless Device. Am J Hypertens 2022, 35:407–413.
212. Schubert C, Archer G, Zelis JM, Nordmeyer S, Runte K, Hennemuth A, Berger F, Falk V, Tonino PAL, Hose R, et al: Wearable devices can predict the outcome of standardized 6-minute walk tests in heart disease. NPJ Digit Med 2020, 3:92.
213. Schwartz JE, Muntner P, Kronish IM, Burg MM, Pickering TG, Bigger JT, Shimbo D: Reliability of Office, Home, and Ambulatory Blood Pressure Measurements and Correlation With Left Ventricular Mass. Journal of the American College of Cardiology 2020, 76:2911-2922.
214. Selder JL, Proesmans T, Breukel L, Dur O, Gielen W, van Rossum AC, Allaart CP: Assessment of a standalone photoplethysmography (PPG) algorithm for detection of atrial fibrillation on wristband-derived data. Comput Methods Programs Biomed 2020, 197:105753.
215. Sequeira N, D'Souza D, Angaran P, Aves T, Dorian P: Common wearable devices demonstrate variable accuracy in measuring heart rate during supraventricular tachycardia. Heart Rhythm 2020, 17:854-859.
216. Shan R, Ding J, Weng D, Spaulding EM, Wongvibulsin S, Lee MA, Demo R, Marvel FA, Martin SS: Early blood pressure assessment after acute myocardial infarction: Insights using digital health technology. Am J Prev Cardiol 2020, 3:100089.
217. Sher DJ, Radpour S, Shah JL, Pham NL, Jiang S, Vo D, Sumer BD, Day AT: Pilot Study of a Wearable Activity Monitor During Head and Neck Radiotherapy to Predict Clinical Outcomes. JCO Clin Cancer Inform 2022, 6:e2100179.

218. Shu L, Yu Y, Chen W, Hua H, Li Q, Jin J, Xu X: Wearable Emotion Recognition Using Heart Rate Data from a Smart Bracelet. Sensors (Basel) 2020, 20:718.
219. Shufelt CL, Kim A, Joung S, Barsky L, Arnold C, Cheng S, Dhawan S, Fuller G, Speier W, Lopez M, et al: Biometric and Psychometric Remote Monitoring and Cardiovascular Risk Biomarkers in Ischemic Heart Disease. J Am Heart Assoc 2020, 9:e016023.
220. Shui X, Zhang M, Li Z, Hu X, Wang F, Zhang D: A dataset of daily ambulatory psychological and physiological recording for emotion research. Sci Data 2021, 8:161.
221. Shumate T, Link M, Furness J, Kemp-Smith K, Simas V, Climstein M: Validity of the Polar Vantage M watch when measuring heart rate at different exercise intensities. PeerJ 2021, 9:e10893.
222. Sjöberg V, Westergren J, Monnier A, Lo Martire R, Hagstr √ ∂mer M, √ Ñng BO, Vixner L: Wrist-Worn Activity Trackers in Laboratory and Free-Living Settings for Patients With Chronic Pain: Criterion Validity Study. JMIR Mhealth Uhealth 2021, 9:e24806.
223. Slade AD, Cardinal JR, Martin CR, Presson AP, Allen CD, Lowrance WT, Dechet CB, O'Neil BB: Feasibility of wearable activity trackers in cystectomy patients to monitor for postoperative complications. Curr Urol 2021, 15:209-213.
224. So TY, Li MYE, Lau H: Between-subject correlation of heart rate variability predicts movie preferences. PLoS One 2021, 16:e0247625.
225. Sokas D, Paliakaitė B, Rapalis A, Marozas V, Bailón R, Petrėnas A: Detection of Walk Tests in Free-Living Activities Using a Wrist-Worn Device. Front Physiol 2021, 12:706545.
226. Sola J, Vybornova A, Fallet S, Polychronopoulou E, Wurzner-Ghajarzadeh A, Wuerzner G: Validation of the optical Aktiia bracelet in different body positions for the persistent monitoring of blood pressure. Sci Rep 2021, 11:20644.
227. Song C, Yu Y, Lu BC, Yan XL: Validation of the Globalcare GCE603 automated blood pressure monitor for self-measurement according to the European Society of Hypertension International Protocol revision 2010. Blood Pressure Monitoring 2020:291-294.
228. Spaccarotella C, Polimeni A, Mancuso C, Pelaia G, Esposito G, Indolfi C: Assessment of Non-Invasive Measurements of Oxygen Saturation and Heart Rate with an Apple Smartwatch: Comparison with a Standard Pulse Oximeter. J Clin Med 2022, 11:1467.
229. Stark K, Czermak T, Massberg S, Orban M: Watch out for ST-elevation myocardial infarction: a case report of ST-elevation in single-lead electrocardiogram tracing of a smartwatch. Eur Heart J Case Rep 2020, 4:1-4.
230. Stollfuss B, Richter M, Dr √ ∂mann D, Klose H, Schwaiblmair M, Gruenig E, Ewert R, Kirchner MC, Kleinjung F, Irrgang V, Mueller C: Digital Tracking of Physical Activity, Heart Rate, and Inhalation Behavior in Patients With Pulmonary Arterial Hypertension Treated With Inhaled Iloprost: Observational Study (VENTASTEP). J Med Internet Res 2021, 23:e25163.
231. Stone JD, Ulman HK, Tran K, Thompson AG, Halter MD, Ramadan JH, Stephenson M, Finomore VS, Jr., Galster SM, Rezai AR, Hagen JA: Assessing the Accuracy of Popular Commercial Technologies That Measure Resting Heart Rate and Heart Rate Variability. Front Sports Act Living 2021, 3:585870.
232. Sun S, Folarin AA, Ranjan Y, Rashid Z, Conde P, Stewart C, Cummins N, Matcham F, Dalla Costa G, Simblett S, et al: Using Smartphones and Wearable Devices to Monitor Behavioral Changes During COVID-19. J Med Internet Res 2020, 22:e19992.
233. Tabara Y, Matsumoto T, Murase K, Setoh K, Kawaguchi T, Kosugi S, Nakayama T, Hirai T, Wakamura T, Chin K, Matsuda F: Home device-monitored sleep blood pressure reflects large artery stiffness: The Nagahama study. Journal of Hypertension 2020, 38:2459-2464.
234. Takahashi H, Saito K, Hishiki Y: Validation of Omron HEM-7600T, a wearable device for monitoring blood pressure, according to the American National Standards Institute/Association for the Advancement of Medical Instrumentation/International Organization for Standardization 81060-2:2013 protocol in the general population without arrhythmias. Blood Press Monit 2021, 26:156-159.
235. Takami M, Fukuzawa K, Kiuchi K, Takemoto M, Nakamura T, Sakai J, Yatomi A, Nakasone K, Sonoda Y, Yamamoto K, et al: Practical Utility of the Postal Service in Delivering a Self-Fitted, Wearable, Long-Term Electrocardiogram Monitoring Device for Outpatient Care. Circ Rep 2021, 3:294-299.
236. Tan BH, Young A, Bianchi E, Brown L, Tatham AJ: Fluctuation in Blood Pressure and Intraocular Pressure in Normal Tension Glaucoma Using Ambulatory Monitoring. Journal of Glaucoma 2021, 30:304-311.
237. Tayal M, Mukherjee A, Chauhan U, Uniyal M, Garg S, Singh A, Bhadoria AS, Kant R: Evaluation of Remote Monitoring Device for Monitoring Vital Parameters against Reference Standard: A Diagnostic Validation Study for COVID-19 Preparedness. Indian J Community Med 2020, 45:235-239.

238. Teo VH, Teo SH, Burkill SM, Wang Y, Chew EA, Ng DW, Tang WE, Koh GC: Effects of technology-enabled blood pressure monitoring in primary care: A quasi-experimental trial. J Telemed Telecare 2021:1357633X211031780.
239. Toba A, Ishikawa J, Suzuki A, Harada K: Automated office blood pressure measurement by elderly patients in the waiting room. Blood Pressure Monitoring 2021, 26:321–327.
240. Tomitani N, Kanegae H, Suzuki Y, Kuwabara M, Kario K: Stress-Induced Blood Pressure Elevation Self-Measured by a Wearable Watch-Type Device. Am J Hypertens 2021, 34:377-382.
241. Treskes RW, van Winden LAM, van Keulen N, van der Velde ET, Beeres S, Atsma DE, Schalij MJ: Effect of Smartphone-Enabled Health Monitoring Devices vs Regular Follow-up on Blood Pressure Control Among Patients After Myocardial Infarction: A Randomized Clinical Trial. JAMA Netw Open 2020, 3:e202165.
242. Trudel X, Brisson C, Gilbert-Ouimet M, Vezina M, Talbot D, Milot A: Long Working Hours and the Prevalence of Masked and Sustained Hypertension. Hypertension 2020:532-538.
243. Tsai CH, Chen PC, Liu DS, Kuo YY, Hsieh TT, Chiang DL, Lai F, Wu CT: Panic Attack Prediction Using Wearable Devices and Machine Learning: Development and Cohort Study. JMIR Med Inform 2022, 10:e33063.
244. Tseng VW, Costa JDR, Jung MF, Choudhury T: Using Smartphone Sensor Data to Assess Inhibitory Control in the Wild: Longitudinal Study. JMIR Mhealth Uhealth 2020, 8:e21703.
245. Ushigome E, Kitagawa N, Kitae A, Kimura T, Iwai K, Oyabu C, Ushigome H, Yokota I, Hamaguchi M, Asano M, et al: Seasonal variation in home blood pressure and its relationship with room temperature in patients with type 2 diabetes. Diab Vasc Dis Res 2020, 17:1479164119883986.
246. Van Chien D, Thanh Binh N, Dung N, Truong Son P: Applicability of a Novel Wearable Wireless Electrocardiogram Monitoring Device (Spyder) for Arrhythmia Detection in Patients with Suspected Cardiac Arrhythmias. Cardiol Res Pract 2021, 2021:8496351.
247. Varas-Diaz G, Kannan L, Bhatt T: Effect of Mental Fatigue on Postural Sway in Healthy Older Adults and Stroke Populations. Brain Sci 2020, 10:388.
248. Varga P, Rosianu H, Vesa S, Hancu B, Beyer R, Pop C: The impact of continuous positive airway pressure on cardiac arrhythmias in patients with sleep apnea. Journal of Research in Medical Sciences 2020, 25:42.
249. Vischer AS, Rosania J, Socrates T, Blaschke C, Eckstein J, Proust YM, Bonnier G, Proen √ ßa M, Lemay M, Burkard T: Comparability of a Blood-Pressure-Monitoring Smartphone Application with Conventional Measurements-A Pilot Study. Diagnostics (Basel) 2022, 12:749.
250. Vlahoyiannis A, Aphamis G, Eddin DA, Giannaki CD: The effect of evening cycling at different intensities on sleep in healthy young adults with intermediate chronobiological phenotype: A randomized, cross-over trial. J Sports Sci 2021, 39:192-199.
251. Vodička S, Susič AP, Zelko E: Implementation of a Savvy Mobile ECG Sensor for Heart Rhythm Disorder Screening at the Primary Healthcare Level: An Observational Prospective Study. Micromachines (Basel) 2021, 12:55.
252. Vorwerg S, Stamm O, Menant A, Alex S, Müller-Werdan U: Observational study in cardiac rehabilitation groups phase III: a comparison of perceived and measured training intensity during a moderate-intensity workout. Eur J Phys Rehabil Med 2021, 57:414-423.
253. Vybornova A, Polychronopoulou E, Wurzner-Ghajarzadeh A, Fallet S, Sola J, Wuerzner G: Blood pressure from the optical Aktiia Bracelet: a 1-month validation study using an extended ISO81060-2 protocol adapted for a cuffless wrist device. Blood Pressure Monitoring 2021, 26:305–311.
254. Wan C, Chen D, Huang Z, Luo X: A Wearable Head Mounted Display Bio-Signals Pad System for Emotion Recognition. Sensors (Basel) 2021, 22:142.
255. Wang C, Lizardo O, Hachen DS: Using Fitbit data to monitor the heart rate evolution patterns of college students. J Am Coll Health 2022, 70:875-882.
256. Wattanapanyawech J, Thaveeratitham P: The Effect Of Breathing Cycles Program on Heart Rateand Sleep Parameters in Healthy Young Adults. Sleep Medicine Research 2020, 11:108–115.
257. Weng D, Ding J, Sharma A, Yanek L, Xun H, Spaulding EM, Osuji N, Huynh PP, Ogunmoroti O, Lee MA, et al: Heart rate trajectories in patients recovering from acute myocardial infarction: A longitudinal analysis of Apple Watch heart rate recordings. Cardiovasc Digit Health J 2021, 2:270-281.
258. Weng W, Blanchard C, Reed JL, Matheson K, McIntyre C, Gray C, Sapp JL, Gardner M, AbdelWahab A, Yung J, Parkash R: A virtual platform to deliver ambulatory care for patients with atrial fibrillation. Cardiovasc Digit Health J 2021, 2:63-70.
259. Wilson-Anumudu F, Quan R, Cerrada C, Juusola J, Castro Sweet C, Bradner Jasik C, Turken M: Pilot Results of a Digital Hypertension Self-management Program Among Adults With Excess Body Weight: Single-Arm Nonrandomized Trial. JMIR Form Res 2022, 6:e33057.

260. Winter JL, Healey JS, Sheth TN, Velianou JL, Schwalm JD, Smith A, Reza S, Natarajan MK: Remote Ambulatory Cardiac Monitoring Before and After Transcatheter Aortic Valve Replacement. CJC Open 2020, 2:416-419.
261. Wong A-Y, Ling S-K, Louie L-T, Law G-K, So R-H, Lee D-W, Yau F-F, Yung P-H: Impact of the COVID-19 pandemic on sports and exercise. Asia-Pacific Journal of Sports Medicine, Arthroscopy, Rehabilitation and Technology 2020, 22:39–44.
262. Yamagami K, Nomura A, Kometani M, Shimojima M, Sakata K, Usui S, Furukawa K, Takamura M, Okajima M, Watanabe K, Yoneda T: Early Detection of Symptom Exacerbation in Patients With SARS-CoV-2 Infection Using the Fitbit Charge 3 (DEXTERITY): Pilot Evaluation. JMIR Form Res 2021, 5:e30819.
263. Yamakoshi T, Rolfe P, Kamiya A, Yamakoshi KI: Volume elastic modulus with exponential function of transmural pressure as a valid stiffness measure derived by photoplethysmographic volume-oscillometry in human finger and radial arteries: potential for arteriosclerosis screening. Med Biol Eng Comput 2021, 59:1585-1596.
264. Yatabe J, Yatabe MS, Okada R, Ichihara A: Efficacy of Telemedicine in Hypertension Care Through Home Blood Pressure Monitoring and Videoconferencing: Randomized Controlled Trial. JMIR Cardio 2021, 5:e27347.
265. Yen HY, Huang WH: The efficacy of commercial smartwatches with a blood pressure-monitoring feature: A pilot randomized controlled trial. J Nurs Scholarsh 2022, 54:324–331.
266. Zhang Q, Arney D, Goldman JM, Isselbacher EM, Armoundas AA: Design Implementation and Evaluation of a Mobile Continuous Blood Oxygen Saturation Monitoring System. Sensors (Basel) 2020, 20:6581.
267. Zhang S, Xian H, Chen Y, Liao Y, Zhang N, Guo X, Yang M, Wu J: The Auxiliary Diagnostic Value of a Novel Wearable Electrocardiogram-Recording System for Arrhythmia Detection: Diagnostic Trial. Front Med (Lausanne) 2021, 8:685999.
268. Zhang S, Zhong Y, Wang L, Yin X, Li Y, Liu Y, Dai Q, Tong A, Li D, Zhang L, et al: Anxiety, home blood pressure monitoring, and cardiovascular events among older hypertension patients during the COVID-19 pandemic. Hypertens Res 2022, 45:856–865.
269. Zhang S, Zhou X, Chen Y, Wang L, Zhu B, Jiang Y, Bu P, Liu W, Li D, Li Y, et al.: Changes in Home Blood Pressure Monitored Among Elderly Patients With Hypertension During the COVID-19 Outbreak: a Longitudinal Study in China Leveraging a Smartphone-Based Application. Circulation Cardiovascular quality and outcomes 2021, 14:e007098.
270. Zhang W, Lei L, Li Y, Wang JG: Validation of the HL868ED upper-arm blood pressure monitor for clinical use and self-measurement according to the European Society of Hypertension International Protocol revision 2010. Blood Pressure Monitoring 2020, 25:53–57.
271. Zhang Z, Zhang R, Chang CW, Guo Y, Chi YW, Pan T: iWRAP: A Theranostic Wearable Device With Real-Time Vital Monitoring and Auto-Adjustable Compression Level for Venous Thromboembolism. IEEE Trans Biomed Eng 2021, 68:2776-2786.
272. Zhu L, Nathan V, Kuang J, Kim J, Avram R, Olgin JE, Gao J: Atrial Fibrillation Detection and Atrial Fibrillation Burden Estimation via Wearables. IEEE J Biomed Health Inform 2022, 26:2063–2074.
